# Modelling the effects of climate change on the interaction between bacteria and phages with a temperature-dependent lifecycle switch

**DOI:** 10.1038/s41598-025-89307-3

**Published:** 2025-02-21

**Authors:** Andrew Morozov, Areej Ageel, Anna Bates, Edouard Galyov

**Affiliations:** 1 University of Leicester, University Rd, Leicester, LE1 7RH UK; 2 Institute of Ecology and Evolution, Leninsky pr. 33, Moscow, 117071 Russia

**Keywords:** Ecological modelling, Bacteriophages

## Abstract

Ongoing climate change and human activities alter the population dynamics of pathogenic bacteria in natural environments, increasing the risk of disease transmission. Among the key mechanisms of amplification of bacteria in the environment is the alteration of the natural control by their enemies, bacteriophages. Using mathematical modelling, we explore how climate change and implementation of certain agricultural practices affect interactions of bacteria with phage exhibiting condition-dependent lysogeny, where the type of phage infection lifecycle is determined by the ambient temperature. As a case study, we model alteration to the control of the pathogenic bacteria *Burkholderia pseudomallei* by its dominant phage. *B. pseudomallei* causes melioidosis, which is among the deadliest infections in Southeast Asia and across the tropics. We use historical records for UV radiation and temperature in Thailand covering the period 2009–2023 to assess the density of the phage-free pathogen, capable of causing infection. We also predict phage-pathogen dynamics for the period 2024–2044. We apply both non-spatial and spatial models to mimic *B. pseudomallei* population dynamics in the surface water of rice fields and in soil. Our models predict a drastic increase in pathogen density due to less efficient control by the phage which is caused by global warming. We also find that some of the current agricultural practices would enhance the risk of acquisition of melioidosis by altering densities of the pathogen in the environment.

## Introduction

Ongoing climate change, in particular, global warming and climatic hazards, have significantly increased the risk of humans acquiring infectious diseases. This is especially true for the diseases caused by environmental pathogens, for which even small modifications of habitat and/or living conditions may have dramatic consequences^1–5^. Exacerbation of infections by environmental pathogens, due to global warming, can occur via different mechanisms. For example, global warming can accelerate the life cycle of pathogens, promote pathogen-vector interactions, and enhance living conditions for pathogenic microorganisms^3,6,7^. Global climate change can also alter the way in which pathogen density is naturally regulated in an environment^8^. For example, ocean warming was reported to facilitate the occurrence of harmful algal blooms as a result of the weakening of top-down control of phytoplankton by grazers (zooplankton) and/or marine viruses^8,9^.

The effect of an increase in ambient temperature on the risk of pathogen proliferation and their regulation and control by natural predators, parasites, or competitors can be complicated, for example, resulting in alteration of types of interactions between species. A practically important scenario is one whereby the control of pathogenic bacteria by phages (bacterial viruses) involves the phenomenon of condition-dependent lysogeny. Condition-dependent lysogeny occurs when the type of infection cycle (lytic or lysogenic) is determined by the ambient temperature^10^. Therefore, at warm temperatures, phages infect bacterial cells and go through a lytic cycle, resulting in lysis of the infected cells and a release of free phages. However, at colder conditions, phages mostly lysogenise their host: under this scenario, the phage remains inside the bacterial cell without causing its lysis.

The most well-known case study of temperature-dependent lysogeny is the system comprising the highly pathogenic bacterium *Burkholderia pseudomallei* and its dominant phage AMP1^10,11^. *B. pseudomallei* causes melioidosis, a serious human illness in Southeast Asia and across the tropics. Disease acquisition by humans occurs by receiving phage-free bacteria from the environment, whereas arrival of a lysogenised pathogen inside a warm-blooded host will present much less risk. Indeed, in a warm environment, inside the host (with a temperature around $37 \ ^\circ C$), the coexistence between lysogenised *B. pseudomallei* and AMP1-like phages would switch to the lytic infection cycle with an eventual lysis of the pathogen, without harming the host^10^. Overall, *B. pseudomallei* is estimated to cause 90,000 deaths per year^12,13^. Despite remarkable pathogen surveillance efforts, the number of cases of melioidosis is increasing globally, in particular due to the ongoing global increase in the prevalence of diabetes, a major factor facilitating disease acquisition^14^. Until recently, the regulation of the density of *B. pseudomallei* by phages in the environment and the role of phages in the infectivity of the pathogen have been largely overlooked. However, available empirical data suggest that phages can potentially control the *B. pseudomallei* density in water or soil in a similar way that marine viruses regulate phytoplankton blooms^15,16^. It was also determined experimentally that the switch between the lytic and lysogenic infection cycles of the AMP1-like phages dominating the environment and controlling the deadly pathogen *B. pseudomallei* occurs at temperatures close to $35 \ ^\circ C$^10,11^. Therefore, in geographic areas with temperatures oscillating around $35 \ ^\circ C$, a further temperature increase due to global climate change is expected to produce a shift towards phage-free pathogen dominance, causing an expansion of endemic areas of melioidosis. On the other hand, it can also be expected that the high abundance of available bacterial hosts, as the consequence of a greater bacterial growth rate caused by the increase in temperature, would enhance production of new free phages, which, in turn, might reduce bacterial numbers. This and other feedback loops in environmental phage-bacteria systems make it extremely challenging to obtain reliable predictions by using only simple ‘hand-waiving’ reasoning, therefore more quantitative tools will be required.

Mathematical modelling is known to be efficient in providing a key to understanding and quantitative prediction of functioning of highly complex biological systems, having multiple non-linear feedbacks, such as microbial communities in non-stationary environments. Overall, application of mathematical models to bacteria-phage systems has been so far successful, exploring various aspects including biodiversity, bacterial resistance, and phage therapy^17–20^. To the best of our knowledge, however, the effect of global climate change on bacteria-phage interactions in natural environments and pathogen control by phages have not been addressed yet (apart from the recent study of temperature-dependent interactions of phytoplankton and marine viruses^8^). Therefore, this study is intended to partly bridge the above-mentioned gap: we will theoretically explore the consequences of global warming on the control of pathogenic bacteria by phages with temperature-dependent lysogeny. Specifically, our modelling study addresses the following questions below.

Firstly, we apply mathematical modelling to explore daily, seasonal and inter-annual oscillations of the density of *B. pseudomallei* in Thailand (which is one of countries most affected by melioidosis) over the past 15 years of ongoing climate change. In our simulations, we use historic data of the surface temperature and UV radiation for the period spanning 2009 to the end of 2023 on an hourly basis for 8 provinces of Thailand. Secondly, we simulate the natural control of this pathogen by phages in Thailand over the next 20 years, starting from 2024, for different scenarios including an increase in the UV index and various levels of nutrients in the environment (determining the carrying capacity of bacteria). To run model simulations for the period of 2024–2044, we forecast daily and seasonal fluctuations in temperature and UV solar radiation based on the available historic data, using time series analysis methods. Thirdly, we explore possible consequences of misuse of agrochemicals for natural control of pathogens by phages. The need for this comes from a recent experimental observation demonstrating that some agrochemicals commonly used in farming, can cause the severe mortality of phages. For example, implementing a high concentration of iron (II), as used in some fertilizers, would deactivate phages, potentially releasing pathogenic bacteria from their biocontrol and amplifying infection risk^21^. We must stress that apart from empirical tests in a lab, the impact of removing phages to the risk of disease acquisition from a natural environment, on large time and space scales, is not well understood.

Mathematically, we implement the modelling approach, which is similar to the one introduced earlier by Egilmez et al.^11,22^. We consider both a non-spatial model (based on ordinary differential equations, ODEs) and a 1-D spatial model (using partial differential equations, PDEs) to describe *B. pseudomallei*-phage interactions in the surface water of rice fields and in the soil, respectively. Importantly, however, we impose several key modifications to the original model by Egilmez et al^11,22^. For example, we consider a more biologically realistic scenario, where phages are unable to kill bacteria at hot temperatures ($>40 \ ^\circ C$), which was disregarded in previous studies.

Overall, our theoretical findings re-enforce the previous message in the literature that global warming and increasing anthropogenic pressure (intensive agriculture) both enhance the risk of infection by environmental pathogens^1,3–5^, in particular, by the deadly bacteria *B. pseudomallei*.

## Methods

### Modelling approach

In this study, we implemented a modified version of the mathematical model from the works of Egilmez and collaborators^11,22^. The state variables of the model are the densities of: (i) phage-free bacteria (*S*) susceptible to infection by the phage; (ii) bacteria infected by the phage in its lysogenic state $I_1$ (containing phage DNA inside); (iii) bacteria infected by the phage in a lytic state $I_2$, and (iv) the concentration of free phages (*P*). The corresponding model equations are provided in Supplementary Material SM1. We consider both the non-spatial and the spatial versions of the model.

The modelling approach implements the following main assumptions^11,22^. At low temperatures, infection of susceptible bacteria *S* by free phages *P* results in lysogeny: this corresponds to transition from *S* to $I_1$. Lysogenic bacteria grow in the same way as phage-free bacteria, which is described by a logistic function with the same carrying capacity. At warmer temperatures, infection of *S* by the phage results in cell lysis and death: in this case new phages are released into the environment. An increase in the temperature leads to a shift from the normal lysogenic cycle of $I_1$ to the lytic cycle. In the model, phages and bacteria die (or become deactivated) due to exposure to ultraviolet solar radiation and other reasons^23^. Importantly, the key parameters of the model, such as bacterial growth rate, infection constants, transition rate between the lytic and lysogenic state, and the natural mortality, depend on the temperature (the corresponding mathematical functions are provided in Supplementary Material SM1).

A major modification of the model by Egilmez and collaborators made in this study, is incorporating the observation that phages become unable to infect bacteria at hot temperatures: such temperature is defined here as being higher than the temperature known to trigger the transition from the lysogenic to the lytic cycle. The inability of phages to infect bacteria at hot temperatures was reported in previous empirical studies^24^ as well as our own lab experiment (detailed in the following subsection). For the coefficients $K_1 (T)$ and $K_2 (T)$ of the model, which characterise the inflow of the lysogenic and lytic bacteria due to infection by the phage, this signifies that their values should be close to zero after exceeding some critical temperature $T_{cr}$. In the updated model, the coefficients $K_1 (T)$ and $K_2 (T)$ can be parameterised as

1 $$\begin{aligned} K_1(T) = \frac{T_{cr}^m}{T_{cr}^m + T^m}\frac{K_S T_1^n}{T_1^n + T^n}, \,\, \,\,\,\, \,\, K_2(T) = \frac{T_{cr}^m}{T_{cr}^m + T^m}\frac{K_S T^n}{T_1^n + T^n}. \end{aligned}$$

In each of the above expressions, the first multiplier describes the effects of hot temperatures ($T>T_{cr}$) on $K_1 (T)$ and $K_2 (T)$. The coefficient *m* describes how fast the switch to a non-infection regime occurs when the critical temperature $T_{cr}$ is achieved. The second multiplier, which remains from the original model used by Egilmez and collaborators, describes the switch between the lysogenic and lytic infections occurring around the temperature $T_1$ ($T_1<T_{cr}$). In both above expressions, $K_S$ is the maximal phage adsorption constant, the temperature $T_1=35 \ ^\circ C$ corresponds to the switch between the lytic and lysogenic cycles^11,22^. The estimates of the parameters $T_{cr}$ and *m* are discussed in the next section from lab experiments.

We intentionally do not incorporate into the model a mathematical term, parameterising the impact of hot temperatures $T> T_{cr}$ on the lysis of already infected bacteria $I_2$. The rationale is that we assume that the rate of temperature variation is slower that of the phage infection cycle. Also, we assume that at hot temperatures, free phages can still attach to bacterial cells, however this will not result in further infection of the cell via any of the cycles (lytic or lysogenic).

In the non-spatial model, describing bacteria-phage interactions in the surface water of rice fields, the influence of solar radiation on phage mortality (expressed by the standard UV index *u*) is estimated by the following expression from Egilmez and collaborators^11^

2 $$\begin{aligned} \mu (u)= {\left\{ \begin{array}{ll} \mu _c + Y_0\exp (ku),& \text {daytime,} \\ \mu _c, & \text {at night,} \end{array}\right. } \end{aligned}$$

where $\mu _c = 0.1 day^{-1}$ is the background (light-independent) mortality. Unlike the original model by Egilmez and collaborators, we differently estimate the parameters $Y_0$ and *k* by taking into account the cloudiness factor from historic data. From the study of^25^, it follows that the survival of bacteria and phages can be estimated to be 5% of the initial population after 0.5 day of exposure to sun radiation in summer and 50% in winter. This gives the values of $\mu _{max}=6.0 \,day^{-1}$ and $\mu _{min}=1.4 \,day^{-1}$ for the estimates for the mortality rates in summer and in winter, respectively. From the insolation data in^25^, one can estimate the difference between the average UV index in summer and winter to be approximately 3 folds. For the considered geographic area in^25^, the UV index can be estimated (‘www.worldweatheronline.com’) to be approximately $u_1=3$ in winter, and, correspondingly, $u_2=3\times 3=9$ in summer (measured in non-dimensional units). Note that an increase in the UV index by a factor of 3 results in the corresponding increase in the mortality by approximately a factor of 4.3, and that agrees with our assumption that the dependence of the mortality on the UV index is a concave upwards function. We can further estimate the values of the coefficients $Y_0$ and *k* from the system of the two following equations: $\mu _{min} \approx Y_0\exp (k u_1)$ and $\mu _{max} \approx Y_0\exp (k u_2)$, where we neglect the background mortality in experiments in^25^. The background mortality parameter $\mu _c$ is difficult to estimate, since it depends on various factors, as adsorption to particles other than bacterial cells, consumption by fagellates or/and amoebas^23^. Following the previous work by Egilmez and collaborators^11^, we consider the default value of $\mu _c=0.1 day^{-1}$.

The influence of solar radiation on bacterial mortality is described in the model by the term $m(u)=Y_0\exp (ku)$, as in the paper by Egilmez and collaborators^11^. There exist experimental confirmation indicating close values for inactivation rates for phages and bacteria (e.g., see the survival experiments using fecal coliforms and somatic coliphages shown in Fig. 2 from^25^). Note that some experimental data show differences between mortality rates of phage and bacteria in experimental settings with light shading; however, such differences become much less pronounced under full sunlight conditions, where inactivation rates are maximal^26^. Therefore, under the current uncertainty and the lack of direct experimental comparison of inactivation rates of AMP1-like phage and *B. pseudomallei*, we make an assumption here that the bacterial mortality due to UV radiation is the same as that of the phage.

In our simulations, we use both non-spatial and spatial models. For the spatial model, which mimics bacteria-phage interactions across the soil depth, we do not include the influence of the UV solar radiation on mortality rates. This is because solar radiation does not penetrate the soil, so we can consider that $\mu (u)=\mu _0$, where $\mu _0$ is a constant value taken from^22^. The vertical distribution of the temperature across soil is modelled via a standard heat equation (see SM1.2 for detail) The other parameters for both non-spatial and the spatial models are taken from^11,22^ and are also listed in SM1.3 (Table  S1). The initial conditions for the non-spatial and spatial models are provided in supplementary material SM1.1 and SM1.2, respectively. Note that we ran model simulations firstly from the beginning ($t=0$) over the period of 3 years, corresponding to the meteorological conditions of year 2009 for the province under consideration, before proceeding to further years of the same province. This was done to avoid possible influence of transient dynamics and initial conditions.

To incorporate the temporal variation of temperature and UV solar radiation in the model, we use the historic time series for the period 2009–2023 and our forecasting for the years 2024–2044 (see the section ‘Study area, temperature and UV solar radiation data’ in “Methods”).

### Empirical investigation of susceptibility of *B. pseudomallei* to phage infection at high temperatures

Environmental temperatures in Thailand and other tropical areas can sometimes attain high values (e.g. $T > 37\ ^\circ C$), for which it was previously reported that many phages lose their ability to infect the bacterial host^24^. Therefore, to better model and predict bacteria-phage interaction in the considered system, it is important to understand the effect of high temperatures on infection of *B. pseudomallei* by AMP1. To investigate this, we performed a phage spot test experiment in the laboratory. We used *B. thailandensis* E264 as a proxy of *B. pseudomallei*. Note that *B. thailandensis* is a recognised model organism for experimental work with *B. pseudomallei*, including studies of bacteria-phage interactions^10,27^. The information on the supplier of the bacterial strain and the phage is provided in supplementary material SM2.

Serial dilutions of the stock sample of the phage were spotted onto 5 identical plates with *B. thailandensis* E264 bacterial lawns, then one plate was incubated at $37 \ ^\circ C$, and the others at higher temperatures, each with $1 \ ^\circ C$ step increment. Technically, 300 $\upmu l$ of an overnight culture of *B. thailandensis* E264 was mixed with 8 *ml* of melted 0.4% (w/v) LB agar supplemented with 1 *mM* CaCl_2_ and distributed evenly over 1.5% LB agar plates and left to solidify. 10-fold serial dilutions were made of the phage lysate using SM buffer. 10 $\mu l$ droplets of each dilution to 10-5 were spotted onto the pre-prepared bacterial lawns in triplicate. The five plates were incubated at temperatures ranging from $37 \ ^\circ C$ to $41 \ ^\circ C$ overnight. Pfu/ *ml* for each temperature was calculated the next day and EOP was determined.

The results of this experiment are presented in the following section ‘Infection of bacteria by the phage at high temperatures ($T \ge 37 ^\circ C$)’. In particular, the critical temperature $T_{cr}$ was estimated to be $T_{cr}\approx 40 \ ^\circ C$. As for the switch between the lysogenic and lytic infection cycles at $T_0\approx 35 \ ^\circ C$ (see the study by Egilmez et al^11^), we parameterise the cease of infection by phage at high temperatures by the sigmoid function $F(T,T_{cr})=\frac{T_{cr}^m}{T_{cr}^m + T^m}$, where we set $T_{cr}= 40 \ ^\circ C$ and assume that $n=m=55$. This is justified by the narrow range of temperatures corresponding to the switch (see the Results for more details).

### Study area, temperature and UV solar radiation data

In this study, we model dynamics of bacteria-phage interactions in eight provinces of Thailand: Sa Kaeo, Nakhon Phanom, Bangkok, Mukdahan, Ubon Ratchathani, Si Sa Ket, Buri Ram, and Roi Et. Six of these provinces (Nakhon Phanom, Mukdahan, Ubon Ratchathani, Si Sa Ket, Buri Ram, and Roi Et) are located in the Northeast region of Thailand, the other two (Sa Kaeo and Bangkok) are geographically placed in the Central region of the country. Both Northeast and Central regions have three seasons each year: the rainy season from May to October (generally characterised by high cloudiness), the cool season from October to February, and the hot season from February to May. A large proportion of Thailand is agricultural land, the majority of which being located in the Northeast region. One of the main types of crops grown in Thailand is rice, which is generally planted from May to August and December to January, and harvested from November to December and from May to June^28^. The majority of rice production (approximately 40% of national production) occurs in the Northeast region, while approximately 25% of national production comes from the central region^29^. Most of this production occurs during the wet season, which accounts for about 80% of the annual production on average. It was also reported that infection by the pathogenic bacteria *B. pseudomallei* often occurs through agricultural activities, in particular, when cultivating rice fields^30^.

We implement the hourly datasets of the ambient temperature and ultraviolet (UV) index covering the period from 2009 to 2023 for the considered eight provinces in Thailand obtained from the website ‘www.worldweatheronline.com’. Note that following the previous study by Egilmez and colleagues^22^, we re-scaled the available temperature data, by setting the highest surface temperature to be higher than the air temperature by an empirical factor $\eta =1.15$.

Using the historic records for the period from 2009–2023, we forecasted the dynamics of the maximal and the minimal daily temperature and the daily UV index for further 20 years starting from year 2024. To achieve this goal, we apply the SARIMA model, which is well-known in time series analysis in environmental sciences^31^. Technically, we implement the auto.arima function from the R package to perform our forecast. The selection of the optimal number of the parameters in the model was determined by considering the lowest score in the Akaike Information Criterion (AIC). The fitting of parameters was done via the backcasting procedure. The diagnostic check was performed on the residuals of these selected models, via the Box–Jenkins approach, by assuring that the residuals are small enough and are randomly distributed according to the normal law^32^.

In our forecast, we assume the existence of a trend for a global raise of the temperature and UV index. The existence of such trends has been extensively reported in the literature on climate modeling. For the trend in the raise of temperature, here we assume the generally accepted prediction that the expected temperature raise would be $1.5 \ ^\circ C$ over 20 years^33,34^. On the other hand, existing predictions of the global trend in the increase of the UV index are much less uniform, with various scenarios being proposed. The main uncertainty in the prediction of the UV index across the globe is related to distinct possible scenarios of the dynamics of the ozone layer in the atmosphere^35,36^. Importantly, the fate of the ozone layer would strongly depend on the control of emissions due to industrial activity, which can hardly (or even not at all) be accurately predicted. To cope with the above-mentioned uncertainty, here we consider several scenarios of the strength of the global raise of the UV index. In particular, we considered the scenarios with a linear trend, where the increase in the UV index over the following 20 years would be 0.25, 3, 6, 6.5 and 8 dimensionless units.

Finally, we should say that following the study by Eglimez and colleagues^11^, we assume that the UV index during daytime is approximately constant and equal to the forecasted value, at night it was set to the minimal value one. We also account for the variation of the length of day time across the year based on the sunset-sunrise time report. We should also say that within the prediction period, daily temperature variation for each day in the model was calculated using the minimal and the maximal temperatures and the sine function.

## Results

### Infection of bacteria by the phage at high temperatures ($T \ge 37 ^\circ C$)

**Fig. 1:**
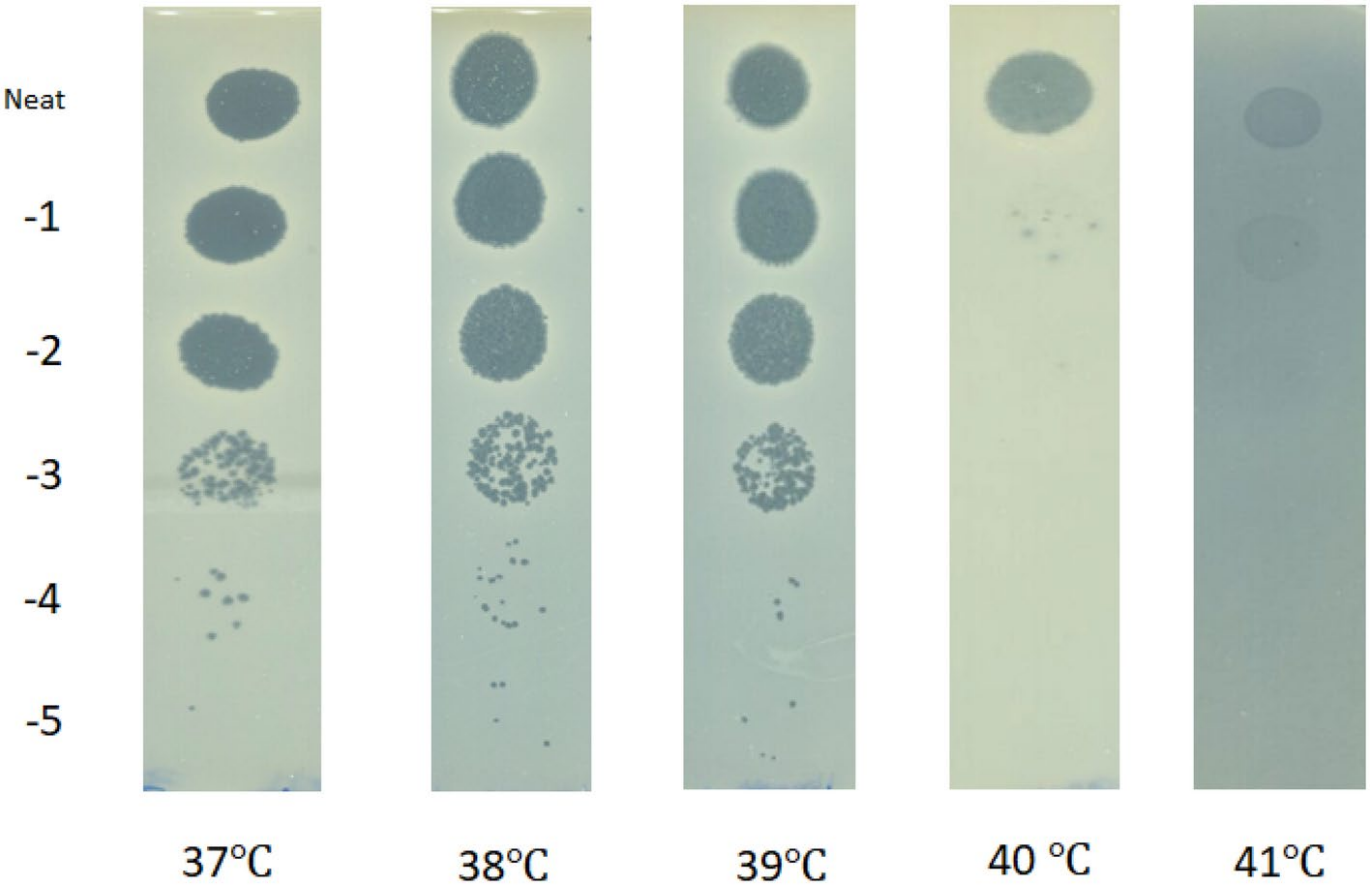
Plaquing efficiency of AMP1 phage on bacterial lawns incubated for 24 hours at various temperatures. Each row represents a different 10-fold dilution.

Our experimental results of infection of *B. thailandensis* E264 by the phage AMP1 at high temperatures ($T \ge 37 ^\circ C$) are presented in Fig. 1, and also in Table  S2 in Supplementary Material SM2. They reveal that AMP1 plaqued with essentially the same efficiency at the temperature range from $37 \ ^\circ C$ to $39 \ ^\circ C$; however, efficiency of plaquing sharply decreased at higher temperatures, dropping by more than 3 orders of magnitude at $40 \ ^\circ C$. No phage plaques were observed at $41 \ ^\circ C$, and the only sign of bacterial lysis, probably caused by the lysis from without mechanism, was noticeable when a neat phage stock sample was used. It is important to note that *B. thailandensis* was able to grow and form bacterial lawns on all plates. Thus, these results suggest that AMP1 cannot exhibit lytic behaviour at temperatures exceeding $40 \ ^\circ C$. This gives us an estimate for the critical temperature $T_{cr}$ (where infection stops) in Eq. (1) to be $T_{cr}\approx 40 \ ^\circ C$.

From the experiment, it also follows that the switch from infection to a non-infection regime occurs suddenly within a narrow temperature interval of approximately $1.5 \ ^\circ C$, which gives justification to model the cease of infection by phage at high temperatures by the sigmoid function $F(T,T_{cr})=\frac{T_{cr}^m}{T_{cr}^m + T^m}$, with $m=55$, simularly to the way of modelling the switch between the lysogenic and lytic infection cycles at the temperature $T_0\approx 35 \ ^\circ C$ (see the study by Egilmez et al.^11^).

### The temperature and UV index variation in Thailand

**Fig. 2:**
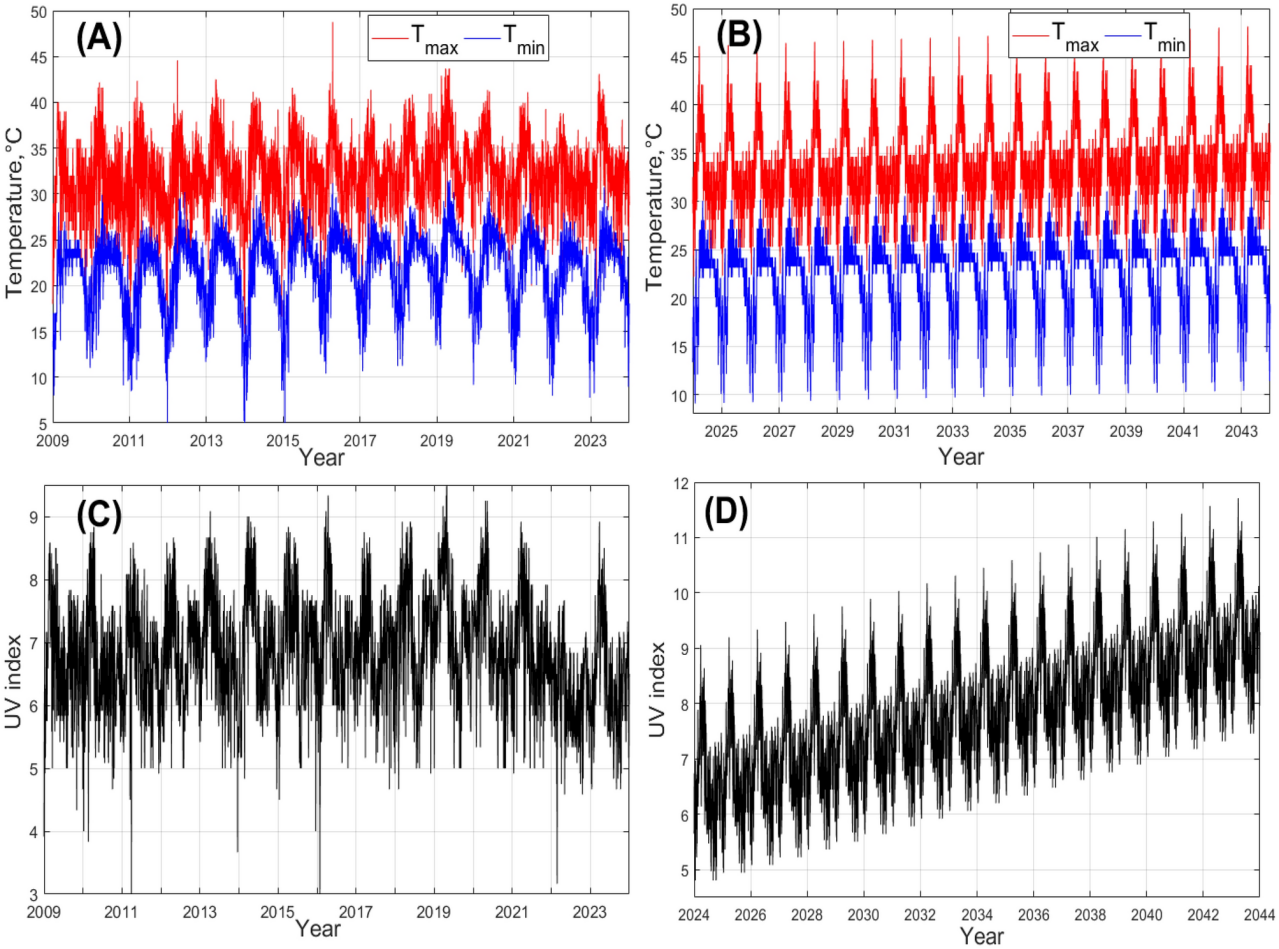
Observed and forecasted temperatures and UV index for the Nakhon Phanom province, Thailand. (**A**) Daily maximum and minimum temperatures from historical data for the period from 2009 to 2023; (**B**) Daily maximum and minimum temperatures for the period 2024 to 2044 forecasted using the best fitted SARIMA model. We assume a linear trend in the temperature raise to be $1.5 \ ^\circ C$ over 20 years. (**C**) Daily average UV index from historical data for the period 2009 to 2023; (**D**) Daily average UV index for the period 2024 to 2044 forecasted using the best fitted SARIMA model. We assume the existence of a linear trend in the raise of UV index to be 3 units over 20 years.

Our analysis of the available meteorological data demonstrates a raise of both the mean temperature and mean UV index between 2009 to 2020 in the eight considered provinces in Thailand (see Fig. S1 from Supplementary Material SM3). This corresponds to the reported global land and ocean surface average temperature anomaly since 2000^37^. This global anomaly ceased in years 2021 and 2022, with a significant drop in both the temperature and solar radiation.

Further, our investigation of the seasonal variation of temperature and the UV index shows that both quantities exhibit clear seasonal cycles, as shown in Fig. 2 (see panels A and C), constructed for the Nakhon Phanom province, as an illustrative example. One can also see that the temperature and the UV index also show pronounced variations on a daily basis. Figues also show that the peak of the temperature and UV index usually occurs in April and May. We should stress that the other considered provinces in Thailand also demonstrate similar patterns of variability in the temperature and UV index.

Finally, Fig. 2 (panels B and D) provides an example of a forecasted pattern of the temperature and UV index variation for the period 2024–2044. Note that in the shown forecast, we assume a linear trend in the raise of the UV index to be 3 units over 20 years. The provided forecast is for the Nakhon Phanom province; for the other considered provinces of Thailand we obtained similar results.

### Modelling bacteria-phage dynamics during the period of 2009–2023

**Fig. 3:**
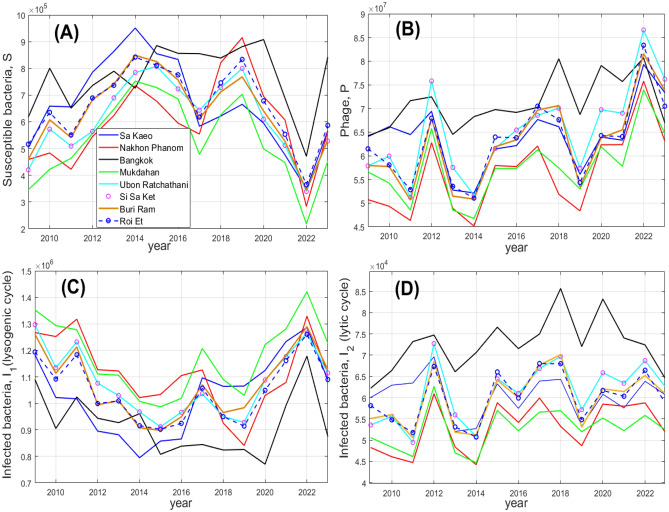
Simulated dynamics of bacterial-phage interactions for years 2009–2023 for the considered eight provinces of Thailand (based on a non-spatial model). In each panel, the annually average values of species densities are plotted for different years. The names of provinces are indicated in the figure label. The unit of the densities of bacteria is cell/ml and phages is pfu/ml.

Using the mathematical model and the historic time series for environmental conditions (the temperature and the UV index), we performed numerical simulations of the bacterial and phage dynamics in Thailand for the period 2009 to 2023. Figure 3 shows the annual average values for the four model components $S,P,I_1,I_2$ for the eight considered provinces of Thailand. Our simulations reveal pronounced inter-annual variability of the bacterial and phage densities over the considered period of 15 years. For most provinces, high values of phage-free bacteria *S* are found to occur for years 2013–2016 and also for 2018–2019; the lowest value of *S* is observed for year 2022. The mentioned inter-annual variability in *S* can be explained by the corresponding variation in the temperature, the total annual number of hot hours (with the temperature $T > 35 \ ^\circ C$) ‘HH’ and the UV index, which can be seen from the calculated Pearson’s correlation coefficients. The corresponding correlation matrix is provided in Supplementary Material SM4 (Fig.  S2 and Table S3). Our estimates of pairwise correlation coefficients between the environmental components (*T*, UV, HH) and the bacterial density *S* are: $\rho _{T,S}=0.71 \pm 0.08$; $\rho _{UV,S}=0.90 \pm 0.03$ and $\rho _{HH,S}=0.83 \pm 0.04$. In particular, the largest suppression of the bacterial density *S* in year 2022 can be explained by the drop in both the UV index and the temperature, signifying a more intensive control of bacteria by phages due to their reduced mortality, lower growth rates of the pathogen as well as the fact that at cooler temperatures (i.e. less amount of hot hours), infection by the phage of a bacterial cell results in lysogeny rather than in lysis. We should also stress that the inter-annual variation of $S,P,I_1,I_2$ shown in Fig. 3 has a pronounced synchronisation across the considered Thailand provinces, which is due the synchronicity of the changes in the environmental factors over considered geographic region (cf. the curves in Fig. S1 from SM3).

**Fig. 4:**
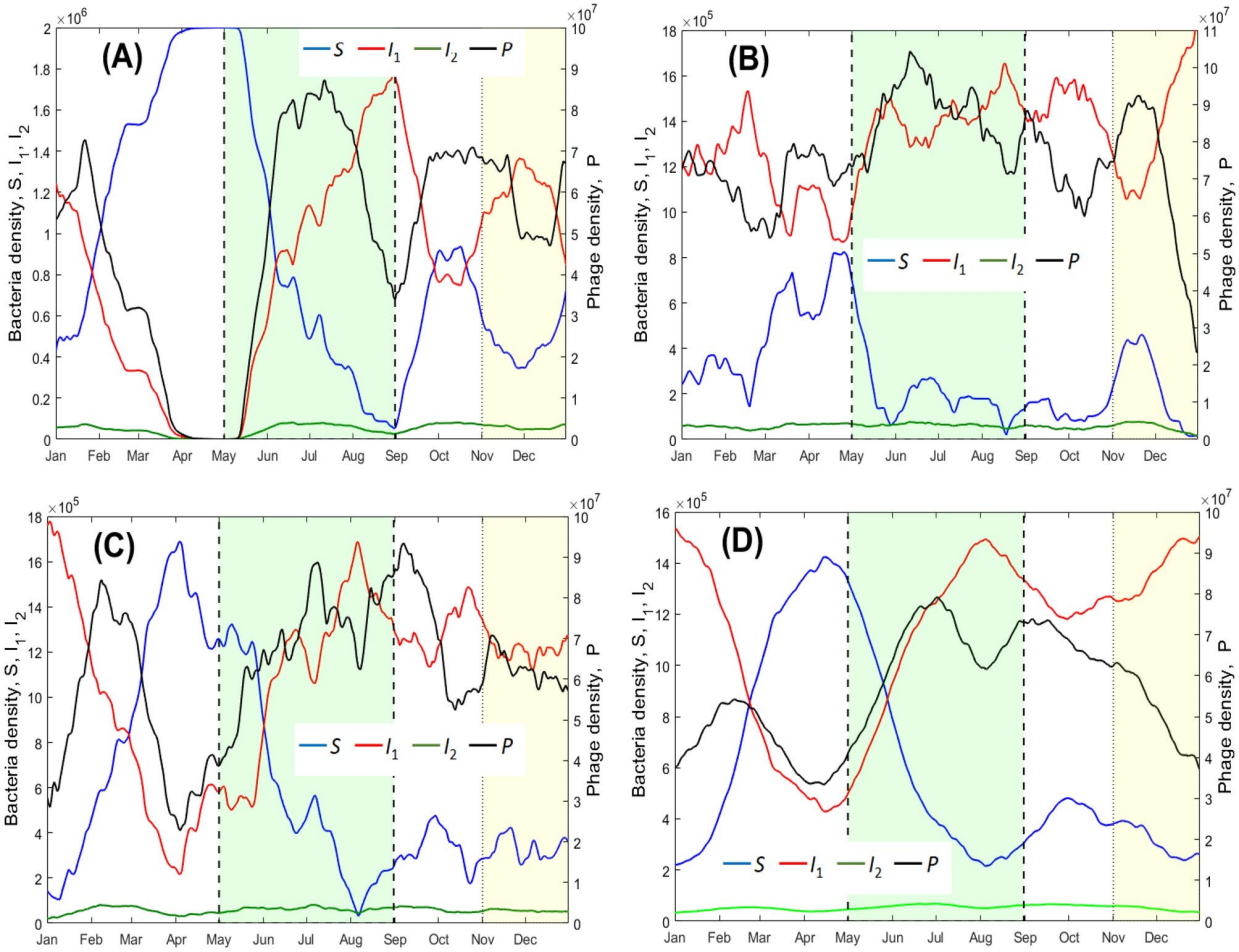
Modelled seasonal variation of the bacteria-phage system for the Nakhon Phanom province (a non-spatial model). Panels (**A**–**C**) provide the dynamics corresponding to years 2021, 2022 and 2019, respectively. These represent examples of typical years with a high, low and a moderate level of *S*, respectively. Panel (**D**) shows the dynamics averaged over the total period for 2009–2023. The green and yellow rectangles indicate typical seasons of agricultural work in rice fields: planting and harvesting, respectively. In each panel, the curves are plotted based on the moving average window technique with the window size of 30 days. The unit of the densities of bacteria is in cell/ml and phages is pfu/ml.

Typical patterns of the annual (seasonal) variation of bacterial-phage interactions are presented in Fig. 4, which is constructed for the temperature and UV data corresponding to the Nakhon Phanom province, as an example. In this figure, panels (A)–(C) provide dynamics for three selected years (years 2019, 2021, and 2022), whereas panel (D) shows the variation of species densities averaged over the total considered 15 year period. Figure 4 schematically shows a typical seasonal schedule of agricultural works in rice fields. In particular, the period of rice planting is denoted by the green rectangle, whereas the yellow rectangle represents the harvesting period. One can see that the model predicts a pronounced seasonal variation of the density *S* of susceptible bacteria as well as that of free phages *P*. High proliferation of *S* is mostly caused by a drop in the number of free phages *P*, which, in turn, is due to hot temperatures, where no infection of bacteria is possible ($T>T_{cr}$), and due to high phage mortality related to increased UV radiation. The annual variation of lysogenic bacteria $I_1$ exhibits the inverse pattern as compared to that of susceptible bacteria *S*. This is explained by the competition between $I_1$ and *S* for the resources, which is described in the by the common carrying capacity *C*. The density $I_2$ of bacteria in a lytic stage is almost constant throughout the year, since the daily temperature variation allows for both lytic and lysogenic infection types each day, which results in a quasi-constant (on average) supply of $I_2$. Major peaks of *S*, which present the most danger for humans, usually occur during the April-May period, which overlaps with the start of the agricultural works. Comparison of panels (A)–(C) demonstrates that the intensity of peaks of *S* largely varies from year to year, i.e. there exist particularly dangerous years (e.g. see panel (A)), where the density of the phage-free pathogen remain around the highest possible value, the carrying capacity *C*, for several weeks.

**Fig. 5:**
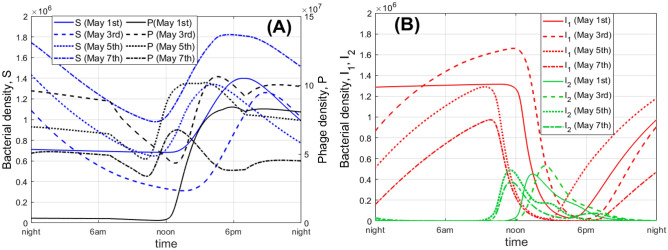
Simulated daily variation of bacterial and phage densities at the beginning of May 2021 (May 1st, 3rd, 5th, and 7th) in the Nakhon Phanom province (a non-spatial model). Panel (**A**) shows the dynamics of *S* and *P*, whereas panel (**B**) shows the dynamics of the infected bacteria $I_1$ and $I_2$. The unit of the densities of bacteria is cell/ml and phages is pfu/ml.

Our simulation also predicts high amplitude daily oscillations of species density. An illustrative example is shown in Fig. 5, constructed for the Nakhon Phanom province, and corresponding to the beginning of May, which is the start of the rice planting season. We present daily variation of $S, I_1, I_2, P$ for May 1st, 3rd, 5th and 7th of year 2021. The model suggests that in the afternoon bacterial infections by phages are mostly lytic. On the contrary, in the morning, late evening and at night, phage infections mostly result in bacteria lysogenisation. Importantly, the susceptible phage-free bacteria *S* attains its peak in the evening (from 5 p.m. to 7 p.m.), and has its minimal density around noon. This makes the evening hours the most dangerous for field works. From the figure, one can also see a high variation of bacterial abundance on a day-to-day basis. This can be explained by the day-to-day variation of the ambient temperature and UV index. Similar patterns of daily variation of $S, I_1, I_2, P$ are predicted by the model for the other provinces and years (the corresponding graphs are not shown here for brevity).

### Forecasting bacteria-phage dynamics for the period 2024–2044

**Fig. 6:**
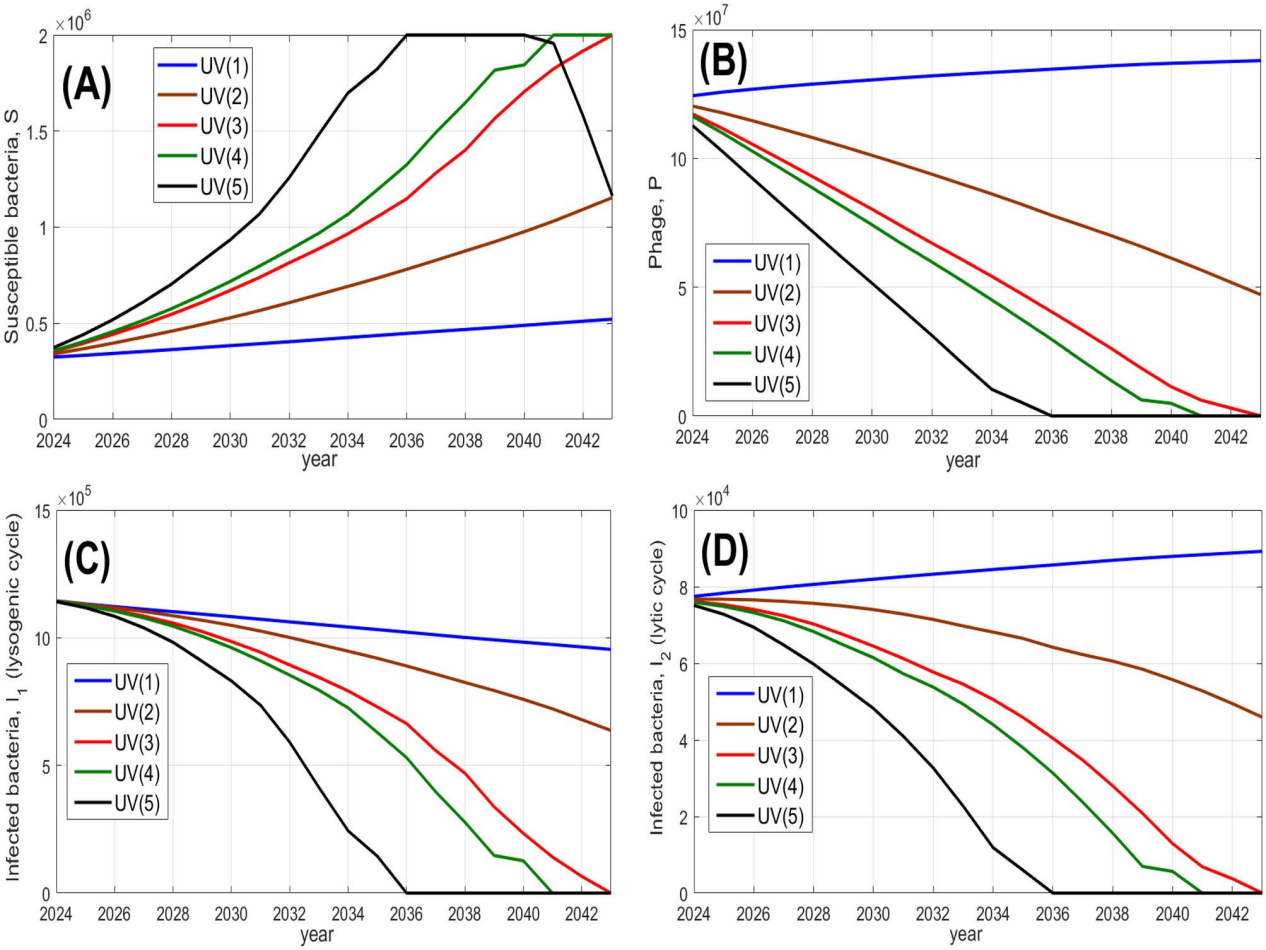
Predicted outcomes of bacterial-phage interactions for years 2024–2044 for the Nakhon Phanom province (based on a non-spatial model). In each panel, the average annual values of species densities ($S,P, I_1, I_2$) are plotted for different years. The predictions are obtained for 5 different scenarios of the global trend in the UV index (denoted by UV(*i*), $i=1,2,3,4,5$), corresponding to an increase of the UV index over 20 years by 0.25, 3, 6, 6.5 and 8 dimensionless units, respectively. The unit of the densities of bacteria is cell/ml and for phages is pfu/ml.

Figure 6 shows simulated model predictions of the bacterial and phage annual average numbers over the next 20 years, starting from year 2024. These predictions use our forecast of the temperature and UV index. We show the results obtained for the Nakhon Phanom province, as an example: our simulations for the other provinces provide similar patterns. The predictions are obtained for 5 scenarios of future trends in the UV index. In Fig. 6, we symbolically denote different trends in the UV index by UV(*i*), $i=1,2,3,4,5$. This describes a range of alteration in the intensity of solar radiation from a light to a strong increase. For all considered scenarios, the temperature trend is kept to $1.5 \ ^\circ C$ over 20 years.

One can conclude from Fig. 6 that for a light increase in UV radiation (scenario UV(1)), both the susceptible bacteria and phage densities show small growth. For a moderate increase in the UV index (scenario UV(2)), the density of phage is predicted to decrease, whereas that of bacteria shows pronounced growth. A stronger positive trend in solar radiation (scenarios UV(3), UV(4)) results in a substantial drop of *P* to the point where the phage goes extinct by the end of the considered time period. The density of susceptible bacteria eventually reaches its carrying capacity, i.e. the largest possible value, and the density of infected bacteria (both lysogenic and lytic) drops to zero. This is related to a pronounced increase in the mortality (deactivation) rate of phages by a very strong UV radiation to the point where an increase of susceptible bacterial host numbers would not compensate the loss due to phage mortality. Finally, for a very strong UV index (scenario UV(5)), the density of susceptible bacteria starts decreasing towards the end of the period of our forecast. This is related to the fact that too strong a level of UV becomes harmful for bacteria. Therefore, at the high levels of the UV index, pathogenic bacteria are regulated by solar radiation rather than by the phage.

Interestingly, the global raise in the temperature by $1.5 \ ^\circ C$ over 20 years has much less impact on the bacteria-phage interactions, than the increase in the UV index. This can be seen, for example, from Fig. 6 for the scenario UV(1): the resultant increase in the density of *S* over the whole period due to the temperature raise for a fairly constant UV level is only about 20%. We also briefly investigated the dependence of our prediction on other model parameters. We found that an increase in the carrying capacity *C*, for example due to extensive use of fertilizers, would amplify the density of susceptible bacteria, resulting in increase of risk of disease acquisition. For high values of carrying capacity, an increase in the temperature and UV index results in the occurrence of outbreaks of high density of *S* throughout the year towards the end of the forecast period. The corresponding graphs can be found in Supplementary Material SM5. We also found that an increase in the burst size would delay the extinction of phages (we do not show the corresponding graphs for brevity).

### Modelling effects of implementation of phage-killing agrochemicals.

**Fig. 7:**
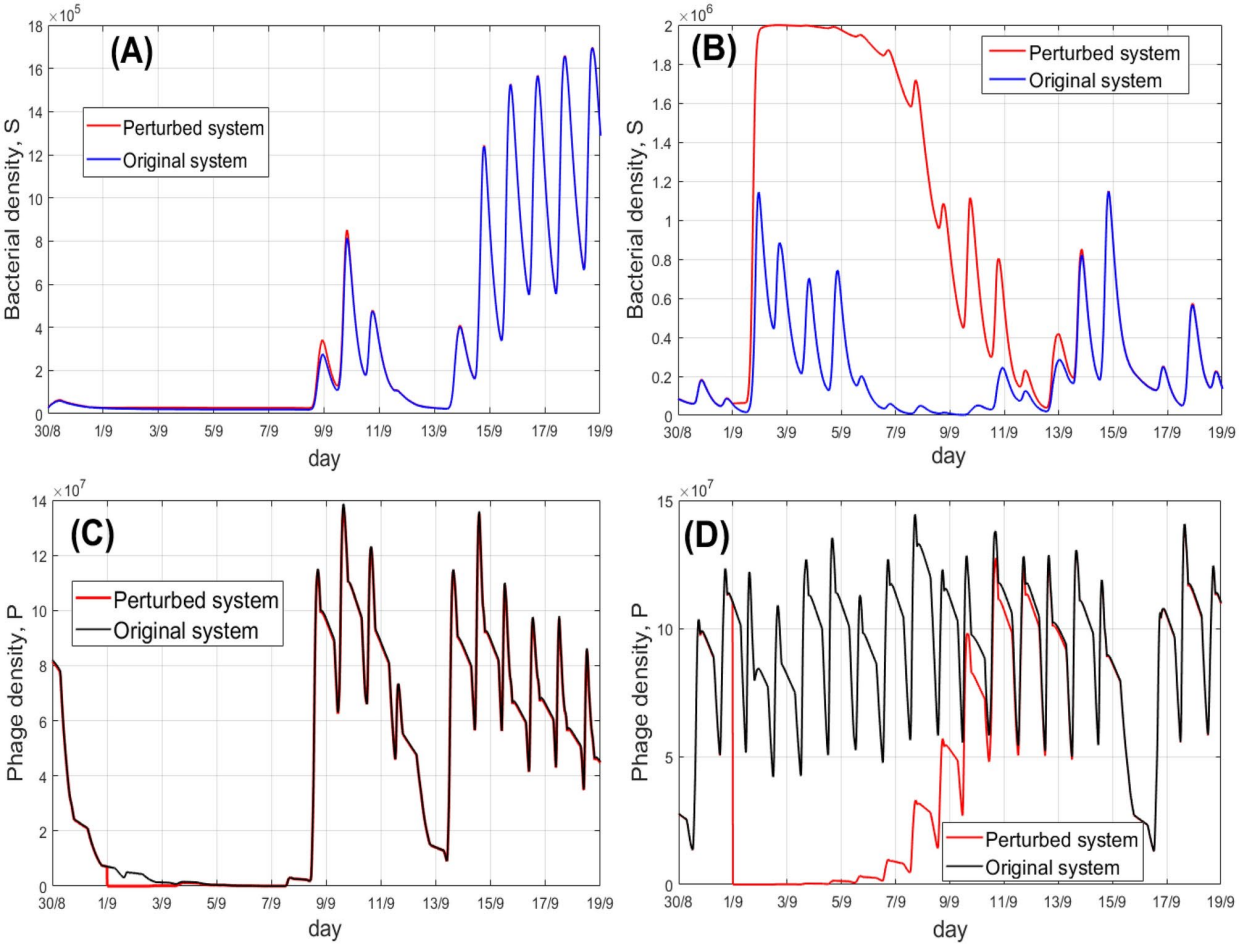
Effects of the implementation of phage-killing agrochemicals on the bacteria-phage dynamics (using the non-spatial model). Free phages are removed from the system on September 1st. In each panel, we show the dynamics of the original (no phage killing) and the perturbed system (with phage killing) in the Nakhon Phanom province. Panels (**A**) and (**C**) are constructed for the year 2019, whereas panels (**B**) and (**D**) are obtained for the year 2021. The unit of the densities of bacteria is cell/ml and phages is pfu/ml.

Implementation of certain agrochemicals, in particular those with a high concentration of iron (II), can cause a severe mortality of phages^21^. In this section, we explore how implementation of phage-killing agrochemicals would affect the risk of infection by the pathogen under realistic fluctuation of weather conditions. We should stress that in this section, we model the effects of phage killing in the surface water of the rice field. We also briefly consider the effects of phage killing on bacteria-phage dynamics in soil.

In our model, we set the free phage density *P* to be zero on the day of release of chemicals. As the date of release, we select September 1st each year, and consider the period from 2009 to 2023. We compare the levels of *S* and *P* in the case of phage removal with the original system, where no phage-killing agrochemicals are applied. Examples of dynamics of *S* and *P*, in the case of removal of free phages, are shown in Fig. 7, constructed for the Nakhon Phanom province. In the figure, the left and right columns, correspond to years 2019 and 2021, respectively. The figure demonstrates two different possible outcomes of killing free phages. Namely, phage removal may have only a small impact on the system, as shown in panels (A) and (C): the species densities in the original and the perturbed systems are close to each other; the level of susceptible bacteria is generally low. Another scenario is demonstrated in panels (B) and (D) of Fig. 7, where removal of free phages results in a pronounced increase in the amount of phage-free bacteria. The level of *S* attains its maximal possible value, the carrying capacity. Importantly, for both above mentioned scenarios, the system demonstrates strong resilience: the initial perturbation of the system due to phage killing eventually becomes negligibly small, so the system returns to its original (i.e. non-perturbed dynamics) course of time. This also indicates the external forcing by the temperature and UV radiation mostly shapes in the system dynamics.

The recovery time of the system after phage removal can be quantitatively estimated by measuring the relative distance $\Delta (t)$ between trajectories of the perturbed (i.e. with phage-killing) and the original systems in the model, which we defined as

3 $$\begin{aligned} \Delta (t)=\sqrt{4\Big ( \frac{S_k(t)-S(t)}{S_k(t)+S(t)}\Big )^2+4\Big ( \frac{I_{1k}(t)-I_1(t)}{I_{1k}(t)+I_1(t)}\Big )^2+4\Big ( \frac{I_{2k}(t)-I_2(t)}{I_{2k}(t)+I_2(t)}\Big )^2+4\Big ( \frac{P_k(t)-P(t)}{P_k(t)+P(t)}\Big )^2}, \end{aligned}$$

where $S_k(t), I_{1k}(t),I_{2k}(t),P_k(t)$ are the species densities in the perturbed system, whereas $S(t), I_1(t), I_2(t),P(t)$ are the densities in the original system, i.e. the one without phage removal. The end of the recovery period can be defined as $\Delta <\Delta _{cr}$, where $\Delta _{cr} \ll 1$ is some fixed critical level. Note that along with the introduced metric (3), usage of other similar metrics is possible.

We estimate the impact of the phage removal on the risk of disease acquisition via the following integral quantity *G*, which we refer to as the integral impact. The integral impact *G* describes how the exposure of humans to the phage-free bacteria changes after killing free phages, as compared to the original system:

4 $$\begin{aligned} G=\int _{t_k}^{t_e} (S_k(t)-S(t))dt, \end{aligned}$$

where $t_k$ is the time of implementation of the agrochemical (i.e. the date of phage removal), $t_e$ is the time at which the system recovers after the perturbation, which is determined by the condition $\Delta (t_e)=\Delta _{cr}$. The introduced integral impact takes into account both the magnitude of the increase in bacterial density after removal of phage and the duration of exposure, i.e. the length of the recovery period.

**Fig. 8:**
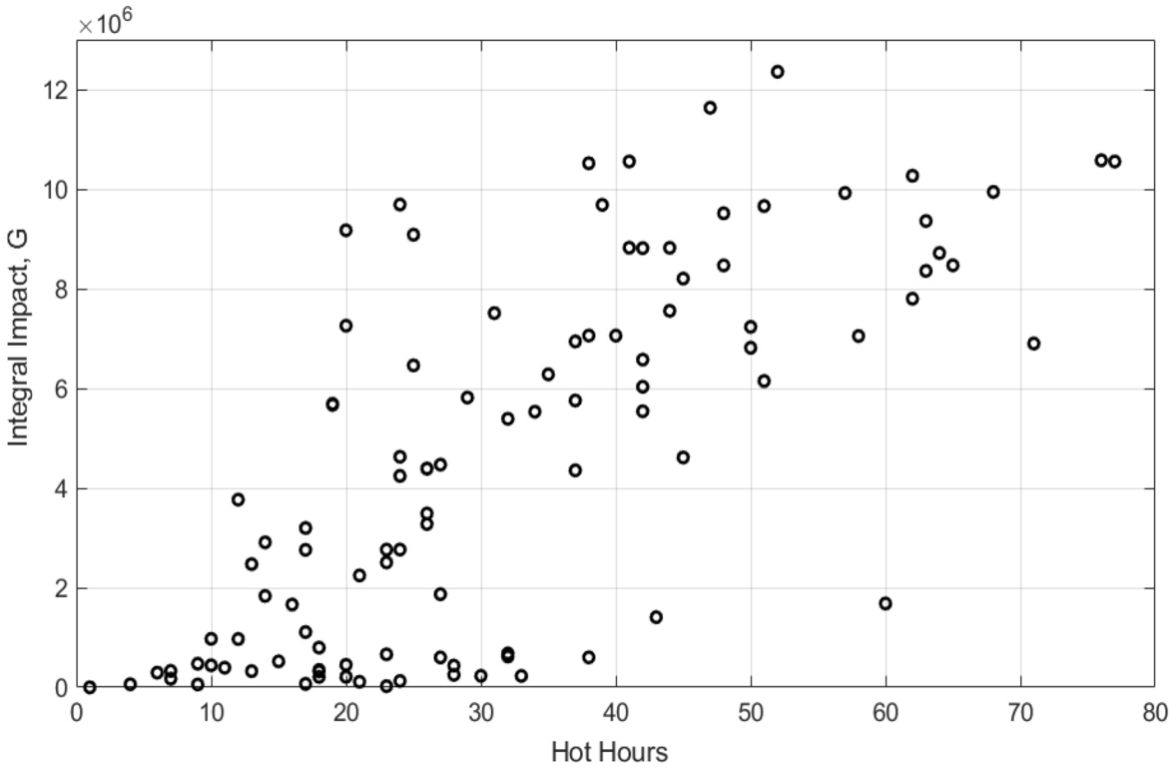
The integral impact *G*, characterising the effect of the killing of free phages by agrochemicals to the risk of disease acquisition, is plotted against the total number of hot hours (defined as hours with $T > 35 \ ^\circ C$) over the period of the system’s recovery. The non-spatial model is used. Simulations are combined for the eight considered provinces of Thailand. For each province, free phages are assumed to be deactivated (killed) on September 1st each year, within the period 2009–2023. The period of recovery of the system after phage killing, has been estimated using the condition $\Delta (t)>\Delta _{cr}$. The mathematical expressions for *G* and $\Delta (t)$ are given by Eqs. (4) and (3), respectively. We assume $\Delta _{cr}=0.25$. The unit of *G* is cells/ml $\cdot$ day.

Our simulation reveals that *G* exhibits a pronounced inter-annual variation, for example it is much smaller for the scenario shown in Fig. 7A,C as compared to the situation presented in Fig. 7B,D. We find that the magnitude of *G* is mainly determined by the temperature, in particular, by the number of hot hours, where $T > 35 \ ^\circ C$. Figure 8 plots the values of *G* for different years (2009–2023) and the eight considered Thailand provinces against the number of hot hours during the period of recovery of the system. One can see that for cool seasons (small numbers of hot hours), the effect of killing phages does not increase the exposure to the bacteria, thus implementation of phage-killing agrochemicals at cooler times does not present extra risk of disease acquisition. This is due to the fact that most bacteria exist as lysogens, therefore the absence of free phages will not largely alter the state of the system. On the contrary, for a supercritical number of hot hours, a large proportion of bacteria exist in a phage-free form *S*. Therefore, setting $P = 0$ will release them from the control by phage, resulting in a rapid amplification of bacteria *S*, attaining the carrying capacity level. This signifies that the application of phage-killing agrochemicals at temperatures $T > 35 \ ^\circ C$ would be potentially risky for agricultural workers.

### Role of vertical spatial heterogeneity

In the previous sections, we modelled bacteria-phage dynamics in the surface water of a rice field, using a non-spatial system, assuming a homogeneous environment. Other scenarios of *B. pseudomallei*-phage regulation are possible, including interaction between microorganisms in soil under a gradient of the temperature, and this, obviously, requires assessing the spatial aspect of the problem. In the current section, we consider *B. pseudomallei*-phage interactions in soil affected by climate change as well as effects of agricultural practices on the risk of disease acquisition. Note that an important consequence of the presence of a vertical gradient of the temperature is the coexistence of predominately lysogenic and lytic types of infection at different soil layers for each moment of time. Another deviation is that bacteria-phage interactions in soil are not affected by the UV solar radiation, so the major environmental factor, externally forcing the system, is the surface temperature. Finally, we must stress that exhaustive analysis of the spatial model should be done in a separate study, therefore here we only provide a short summary of the main findings.

**Fig. 9:**
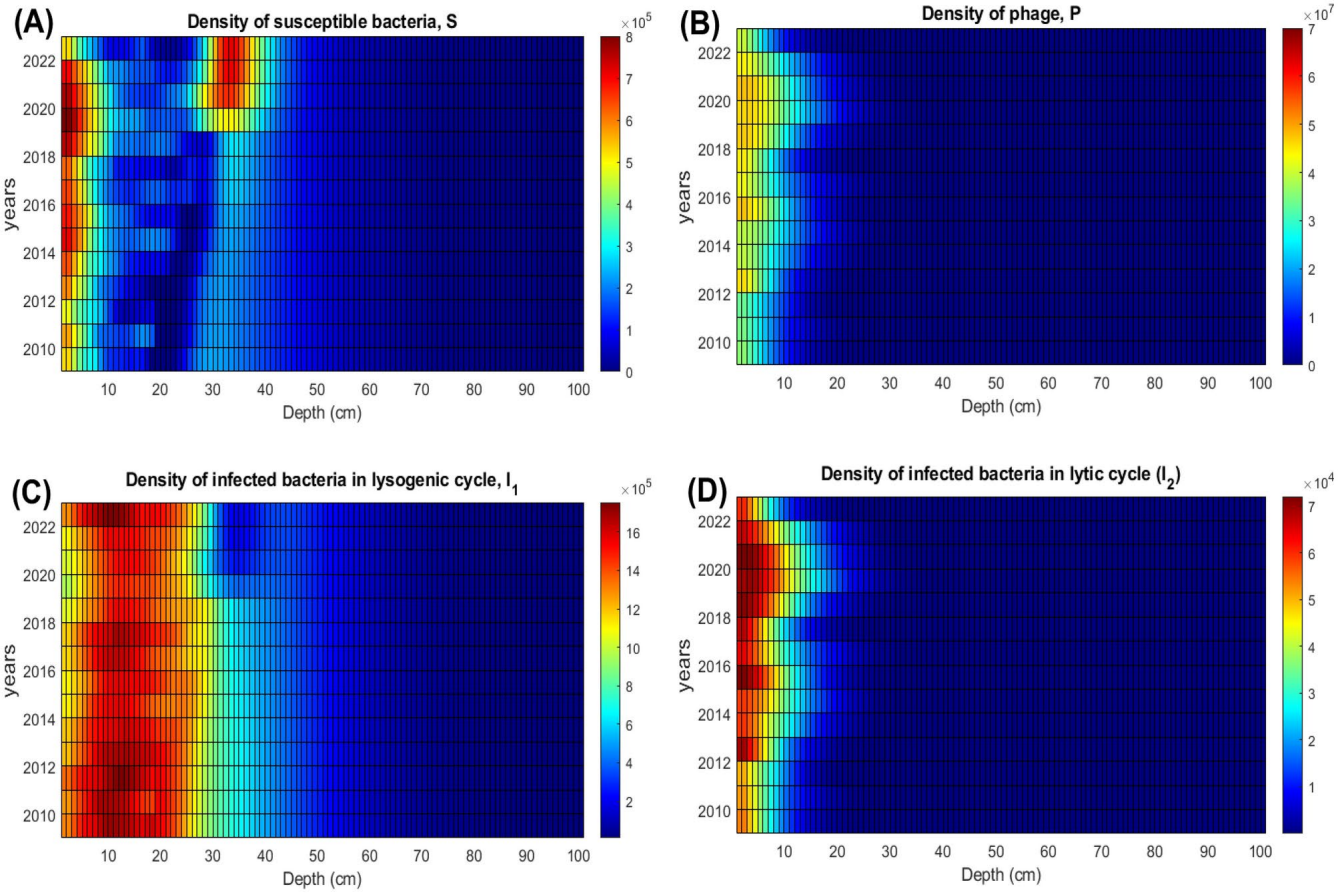
Simulated annual average densities of susceptible bacteria *S*, bacteria infected with phage in the lysogenic cycle $I_1$, bacteria infected with phage in lytic cycle $I_2$ and the phage *P* in soil at different depths, as predicted by the spatially explicit model for the Nakhon Phanom province for the period 2009–2023 using historic temperature data. The unit of the densities of bacteria is cell/ml and for phages is pfu/ml.

The outcome of bacteria-phage interaction in soil for the period 2009 to 2023, using historic hourly temperature data, is presented in Fig. 9, constructed for the Nakhon Phanom province. For each year, at each depth, we show the annual average densities of $S, P, I_1, I_2$. The spatial model predicts the occurrence of two major peaks of the density of susceptible bacteria *S*: one peak arises near the surface, the second one is located at a depth of approximately 30 cm. The near surface peak exhibits pronounced daily and annual fluctuation, whereas the deeper peak changes on a slower timescale. Similar patterns of vertical profiles of $S, P, I_1, I_2$, was found in the work of Egilmez and colleagues^22^, who used a simplified version of the model. The emergence of the deep (the second) peak of *S* occurs since lysogenised bacteria $I_1$ experience extra mortality due to lysis during warm periods. In quasi-absence of free phages *P* at the considered depths (30–40 cm), this gives advantage to the phage-free bacteria *S* in their competition for resources with $I_1$. Note that the above-mentioned extra mortality of $I_1$ due to lysis is generally very small, and it occurs only during relatively short warm periods, where extra heat penetrates the considered depths. This explains the slowness of the emergence of the second peak of *S* predicted by the model.

Examples of the daily variation of vertical distribution of bacterial and phage densities from our simulation are provided in Supplementary Material SM6. Figure 9 demonstrates that the raise of the surface temperature over the period 2009–2023 results in an overall increase of the abundance of susceptible bacteria in soil. The figure also demonstrates that the development of the deep peak of susceptible bacteria is a slow process, which occurs over a period of 12 years. For the other considered provinces of Thailand, the amplitude and the time of formation of the deep peak of *S* largely varies from one province to another (we do not show the corresponding figures for brevity).

**Fig. 10:**
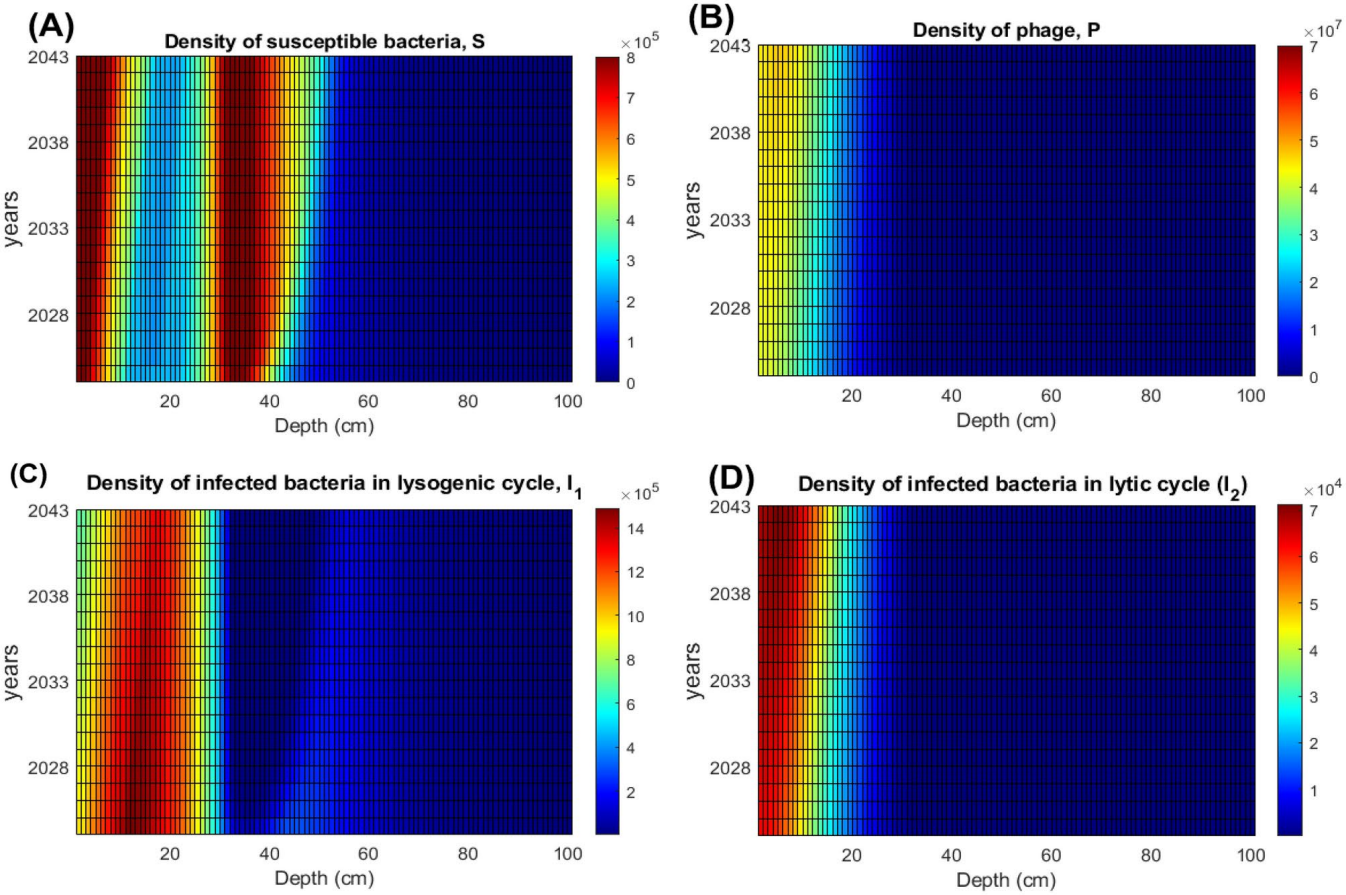
Predicted annual average vertical distributions for the densities of susceptible bacteria *S*, bacteria infected with phage in the lysogenic cycle $I_1$, bacteria infected with phage in the lytic cycle $I_2$ and the phage *P* in soil in the space-explicit model for years 2024–2044. The unit of the densities of bacteria is cell/ml and for phages is pfu/ml.

Using our surface temperature forecast from 2024 to 2044, we predicted the dynamics of bacteria and phage density for this indicated period. The corresponding results are shown in Fig. 10 for the Nakhon Phanom. This figure demonstrates that an increase in the surface temperature caused by climate change results in an intensification of both peaks of the density of susceptible bacteria *S*. The annual average of free phage density in the upper layer of soil follows an increase in the amount of susceptible bacteria. Note that unlike the bacteria-phage dynamics in the surface water, the spatial model does not predict eventual extinction of phage. On the contrary, the total amount of phage towards year 2044 is predicted to increase by approximately a factor of 2. This occurs due to the gradual increase in the amount of susceptible host for the phage because increase in UV radiation would have no effect on either bacterial or phage mortality. The amount of infected bacteria in the lysogenic cycle slightly decreases with time, whereas the numbers of infected bacteria in the lytic cycle show a slight increase. Our simulation of bacterial and phage dynamics in other provinces shows similar patterns of the forecast (we do not show here the corresponding graphs for brevity).

Finally, we modelled the influence of agricultural practices on the risk of disease acquisition in the system with space. Namely, we explored effects of two following agricultural activities: (i) over-turning and mixing the top soil layer due to plowing or shoveling, and (ii) killing the free phages as a result of usage of agrochemicals. In the model, for soil mixing, we assume that each year on May 1st the upper soil layer (from the surface to a depth of 40 cm) becomes mixed. Therefore, on the day of mixing, the densities $S, P, I_1, I_2$ stay uniform in the mixed layer, with the values corresponding to the spatially averaged values in the considered layer before mixing. We found that soil mixing may have a significant impact on bacterial densities at depths between 20cm and 40 cm. On the other hand, the density of susceptible bacteria near the surface recovers quickly and reaches similar levels to those without mixing. We also found that the density of bacteria decreases at depths of 20 cm to 40 cm due to soil mixing. Soil mixing hinders the development of the deep maximum of susceptible bacteria in soil. Examples of dynamics of the system with and without mixing for the Nakhon Phanom are provided in Supplementary Material (SM7).

To model effects of agrochemicals, we eliminated free phages *P* in the vertical layer from the surface up to a depth of 5 cm on September 1st each year. Simulation reveals similar outcomes of phage killing as in the homogeneous model, see the previous section. In particular, the application of phage-killing agrochemicals strongly depends on the ambient temperature. In warmer years, it takes approximately two weeks for the phages to regain their regulation of bacteria, while in colder years, it takes about three weeks. On the other hand, the increase of the density of susceptible bacteria due to phage removal in warmer years can be much higher than in colder years. Therefore, application of agrochemicalsis not recommended during periods of high temperatures ($T > 35 \ ^\circ C$). Examples of dynamics of the system after removal of free phages in the top 5cm of soil are provided in Supplementary Material (SM7).

## Discussion and conclusions

It has been widely recognised that global climate change has profound effects on acquisition of pathogenic diseases by humans from natural environments^4,5^. Surprisingly enough, only very little is known about the potential role of a global raise in temperature and/or UV solar radiation as well as increasing human activities on the regulation and control of environmental pathogenic bacteria by their viruses (phages). To partly fill the gap, we theoretically explore consequences of global warming on the regulation of bacteria by phages with temperature-dependent lysogeny. As a particular case study, we consider regulation of the density of the pathogenic bacteria *Burkholderia pseudomallei* by AMP1-like phages with a temperature-dependent life cycle switch. *B. pseudomallei* causes melioidosis, a disease estimated to be among most fatal in Southeast Asian countries, such as Thailand^13,38^.

We should stress that our study implies a number of key assumptions/simplifications. A major assumption, required for interpretation of the modelling results, is that the risk of disease acquisition by humans is determined by the availability of phage-free pathogen’s in the environment, i.e. by the level of bacterial density *S* in water or in soil. Note that using *S* as a proxy to estimate the number of cases of infection is only correct under the condition that the frequency of visiting the sites with pathogens by humans (e.g. agricultural workers, tourists, etc) remains the same throughout the year^39^. Another key assumption is that in the considered provinces of Thailand, the bacterial numbers are mainly controlled by AMP1-like phages, and not by other biological factors (e.g. competition of *B. pseudomallei* with other bacteria). This also implies that phages are expected to be equally efficient in infecting bacteria and replicating inside the host in all considered environments. Finally, we assumed that the carrying capacity for bacterial growth is temperature-independent and that the value of *C* is the same for each considered province across Thailand. Based on the above assumptions, the following predictions are obtained.

An increase in the average annual temperature should result in a corresponding increase in the amount of phage-free susceptible bacteria, presenting the main risk of melioidosis acquisition by humans. With an increase of the number of hot hours (with $T > 35 \ ^\circ C$) each year, the percentage of the pathogen population in a lysogenic state (which is assumed to be safe for humans) decreases, whereas the percentage of phage-free bacteria increases. The raise in the UV index level, which was found to be positively correlated with temperature (see Fig. S2 from Supplementary Material SM4), has a strong negative effect on phage by increasing its mortality. This impedes the natural regulation of bacteria by the phage. Our model predicts that for a strong global trend in the increase of the UV level (by more than 5.5 dimensionless units over 20 years), the phage would go extinct towards the end of the period 2024–2044 in the surface water layers of agricultural fields. Therefore, the pathogenic bacteria will reach its carrying capacity and will be out of natural control by the phage. This scenario will be particularly dangerous for agricultural workers, who will be exposed to the deadly bacteria within the whole agricultural season. Importantly, even for a scenario, where the UV index increase does not fully eliminate the phage, we still expect an increase in pathogen density over next 20 years due to less efficient control of *B. pseudomallei* by phages. This would eventually signify a spread of current zones of endemicity of melioidosis in Thailand and, possibly, in the region of Southeast Asia, due to global warming.

Our analysis of the historic data for the period 2009–2023 shows that an increase in temperature and/or the UV level in particular years can be substantially higher as compared to the mean increase predicted by a long-term trend. This indicates a major potential risk of global climate change for the disease acquisition in the *B. pseudomallei*-phage system. Indeed, in the future, there can be few years with high anomalies of the temperature and UV index, where the phage density would drop to a very low level, whereas the density of phage-free pathogen would reach its highest possible value, the carrying capacity. Illustrative examples can be seen in Fig. 4. For example, the model shows that in May 2021, the phage density should have dropped to very low values, whereas the density of susceptible bacteria should have attained its highest level of carrying capacity. This signifies a high risk for agricultural workers in the start of the rice planting season in the mentioned year. On the other hand, in the following year, May 2022, the density of *S* should have exhibited smaller values during the same period, due to a substantial drop in UV radiation (mostly because of cloudiness) as well lower temperatures. Models of climate change predict amplification of emergence of short-term anomalies of environmental conditions, i.e. they predict not only a future increase in the mean temperature/UV index values, but also augmentation of their variance^40,41^. Thus, for *B. pseudomallei*-phage interaction, we should expect an even more pessimistic scenario as the one predicted by the model using only the long-term trends in temperature or UV index.

Modeling bacteria-phage interaction across soil gives somewhat more optimistic prediction, as compared to interaction in the surface water of rice fields. Although the generic pattern of the spatial system is a raise in the total amount of phage-free bacteria within next 20 years (2024–2044), the pathogenic bacteria would still remain under control by the phage. The main reasons are: (i) lower value temperature across the soil, as compared to those at the surface, and (ii) the fact that the soil protects phages from UV solar radiation, preventing them from extinction. On the other hand, the model predicts an amplification of the deep peak susceptible bacteria at depths of around 30 cm, making agricultural work riskier in future recent years. Therefore, global warming is predicted to cause gradual replacement of harmless lysogenic bacteria by deadly phage-free bacteria across depths. Interestingly, few previous studies on the incidence rate of melioidosis in Thailand indicated that the incidence rate of the disease was higher in the Northeastern provinces as compared to the central region^42–44^. Our modelling results for the period 2009–2023 show that the deep peak of the density of susceptible bacteria is higher in the Northeastern provinces also, which appears to provide a match between the prediction of the model (in terms of the amount of phage-free bacteria) and the incidence rate of melioidosis.

Our model simulation predicts a clear seasonal pattern of variation in bacteria-phage dynamics, with the highest densities of phage-free bacteria *S* to occurring in the time frame mid-March to mid-May. The period of high abundance of *S* overlaps with the timing of rice planting, requiring more safety measures to be taken. The model predicts high daily oscillations of bacterial and phage densities, making certain times of the day particularly dangerous. For instance, the peak of the density of phage-free bacteria is expected to happen between 5 p.m. to 7 p.m., whereas the lowest densities are attained around noon. Hence, our model-informed recommendation for agricultural workers would be to avoid working during the above-mentioned evening hours of higher infection risk, or to use protective gloves, clothes and footwear when working in the field.

In this study, we used the framework of modelling bacteria-phage interaction as previous theoretical studies by Egilmez and colleagues^11,22^. An important amendment of our model was to incorporate the fact that phages do not infect bacteria at hot temperatures, which modifies corresponding infection terms in the equations. This amendment was supported by our experimental study (see “Methods” for details). Importantly, in this current study we implement historical data on hourly changes in the temperature and UV index, whereas previous works computed the 4-year average (2013–2016) of the mean monthly temperatures of the air and considered the UV index to be the same throughout the day. These amendments to the initial model by Egilmez and colleagues^11,22^ showed somewhat different predictions for annual and daily variation of species densities. For example, our amended model based on historic data showed a high level of the phage-free bacteria density at the start of the planting period (April) with a further drop of *S* in summer. This is due to a more accurate description of the seasonality in Thailand, in particular, seasonal variation of the temperature and UV index. Moreover, the current study reveals a pronounced inter-annual variation of bacterial and phage densities (due to the corresponding variation of the temperature and UV index from year to year), which was previously somewhat overlooked. This emphasizes the importance of using more accurate data for modelling environmental microbial communities. On the other hand, both our model and the study of Egilmez and colleagues^11,22^ share some common features in their prediction of the seasonal dynamics of bacteria-phage interaction, in particular, high peaks of bacterial density *S* during the hot seasons.

Some common agricultural practices can affect natural control of pathogens by phages. Here we model effects of implementation agrochemicals, which cause mortality of phages. The model shows that removal of free phages either in surface water or in soil can result in a quick proliferation of the susceptible bacteria, and the bacterial numbers would be only regulated by the carrying capacity. Therefore, implementation of phage-killing agrochemicals would increase the risk of disease acquisition by agricultural workers. However, the impact of removing free phages is strongly determined by the weather conditions on the days following the application of agrochemicals. If the temperature (measured in numbers of hot hours) is supercritical (see Fig. 8), removal of free phages leads to amplification of susceptible bacteria in the system. In the case, where the temperature on average is low, a sudden phage killing would not largely alter the system state since most of bacteria, initially being in a lysogenic form, would remain in the same (lysogenic) form. Thus, implementation of phage-killing agrochemicals under this condition would be relatively safe. Therefore, a practical recommendation is to avoid implementation of phage-killing agrochemicals under inappropriate weather conditions. We also suggest with a global trend in the increase of the temperature, the negative impact of phage removal will amplify. On the other hand, the spatially explicit model predicts that other agricultural activities, as making the upper layer of soil more homogeneous (by plowing or shoveling), may have positive effects on reduction of the risk of disease infection. For example, soil mixing would slow the development of a deep maximum of susceptible bacteria, which would otherwise represent an additional risk of infection.

Among possible future direction of this research we would like to mention the following. It would be important to explicitly include to the model the availability of humans at endemic sites to assess the risk of diseases acquisition. The rate of contact of humans with environmental pathogens such as *B. pseudomallei*, is suggested to increase due to global intensification of agricultural and other activities. On the other hand, there is an ongoing alteration in the ability of humans to catch infection via contact with a pathogen^45^. For example, the predisposing conditions to melioidosis, such as type 2 diabetes, become increasingly common^46^: the current data predicts strong trends of the prevalence of diabetics, which would signify a higher number of cases in the future even if the total amount of bacterial density remains at the current level. A recent study made good progress in developing future projections of the burden of melodious by taking into account global trends in diabetes as well as predicted age distribution^47^. However, the mentioned study considered a simplified situation by fully disregarding fluctuations in the environmental component of the problem by assuming the bacterial density to be constant, in particular, the authors explain the seasonal variation in melioidosis cases by only seasonal variation of human activity. Here we argue that we need an synergistic approach, which would combine explicit modelling of bacterial/phage density and the availability of humans at endemic sites, as well as the trends in predisposition of humans to be infected by environmental pathogens. Another important next step of the current research would be considering more complicated scenarios of the global climate change, for example, by taking into account a more frequent occurrence of in anomalies of high/low temperature (e.g. heat/cold waves) and the UV index in future years^40^.

## Supplementary Information

Supplementary Information.

## Data Availability

The datasets generated during and/or analysed during the current study are available from the corresponding author on reasonable request.

## References

[1] Altizer, S., Ostfeld, R. S., Johnson, P. T., Kutz, S. & Harvell, C. D. Climate change and infectious diseases: from evidence to a predictive framework. *Science* **341**, 514–519 (2013). 23908230 10.1126/science.1239401

[2] Epstein, P. The ecology of climate change and infectious diseases: comment. *Ecology* **91**, 925–928 (2010). 20426350 10.1890/09-0761.1

[3] Burge, C. A. et al. Climate change influences on marine infectious diseases: implications for management and society. *Ann. Rev. Mar. Sci.* **6**, 249–277 (2014). 23808894 10.1146/annurev-marine-010213-135029

[4] Mora, C. et al. Over half of known human pathogenic diseases can be aggravated by climate change. *Nat. Clim. Chang.* **12**, 869–875 (2022). 35968032 10.1038/s41558-022-01426-1 PMC9362357

[5] Baker, R. E. et al. Infectious disease in an era of global change. *Nat. Rev. Microbiol.* **20**, 193–205 (2022). 34646006 10.1038/s41579-021-00639-z PMC8513385

[6] Chowdhury, F. R., Nur, Z., Hassan, N., von Seidlein, L. & Dunachie, S. Pandemics, pathogenicity and changing molecular epidemiology of cholera in the era of global warming. *Ann. Clin. Microbiol. Antimicrob.* **16**, 1–6 (2017). 28270154 10.1186/s12941-017-0185-1 PMC5341193

[7] El-Sayed, A. & Kamel, M. Climatic changes and their role in emergence and re-emergence of diseases. *Environ. Sci. Pollut. Res.* **27**, 22336–22352 (2020). 10.1007/s11356-020-08896-w PMC7187803 32347486

[8] Demory, D. et al. A thermal trade-off between viral production and degradation drives virus-phytoplankton population dynamics. *Ecol. Lett.* **24**, 1133–1144 (2021). 33877734 10.1111/ele.13722

[9] Ruszkiewicz, J. A. et al. Brain diseases in changing climate. *Environ. Res.* **177**, 108637 (2019). 31416010 10.1016/j.envres.2019.108637 PMC6717544

[10] Shan, J. et al. Temperature dependent bacteriophages of a tropical bacterial pathogen. *Front. Microbiol.* **5**, 599 (2014). 25452746 10.3389/fmicb.2014.00599 PMC4231975

[11] Egilmez, H. I. et al. Temperature-dependent virus lifecycle choices may reveal and predict facets of the biology of opportunistic pathogenic bacteria. *Sci. Rep.* **8**, 9642 (2018). 29941954 10.1038/s41598-018-27716-3 PMC6018541

[12] Limmathurotsakul, D. et al. Systematic review and consensus guidelines for environmental sampling of *Burkholderia pseudomallei*. *PLoS Negl. Trop. Dis.* **7**, e2105 (2013). 23556010 10.1371/journal.pntd.0002105 PMC3605150

[13] Limmathurotsakul, D. et al. Activities of daily living associated with acquisition of melioidosis in northeast thailand: a matched case-control study. *PLoS Negl. Trop. Dis.* **7**, e2072 (2013). 23437412 10.1371/journal.pntd.0002072 PMC3578767

[14] Lin, X. et al. Global, regional, and national burden and trend of diabetes in 195 countries and territories: an analysis from 1990 to 2025. *Sci. Rep.* **10**, 14790 (2020). 32901098 10.1038/s41598-020-71908-9 PMC7478957

[15] Gatedee, J. et al. Isolation and characterization of a novel podovirus which infects *Burkholderia pseudomallei*. *Virol. J.* **8**, 366 (2011). 21791081 10.1186/1743-422X-8-366 PMC3169511

[16] Withatanung, P. et al. Analyses of the distribution patterns of *Burkholderia pseudomallei* and associated phages in soil samples in thailand suggest that phage presence reduces the frequency of bacterial isolation. *PLoS Negl. Trop. Dis.* **10**, e0005005 (2016). 27668750 10.1371/journal.pntd.0005005 PMC5036839

[17] Jensen, M. A., Faruque, S. M., Mekalanos, J. J. & Levin, B. R. Modeling the role of bacteriophage in the control of cholera outbreaks. *Proc. Natl. Acad. Sci.* **103**, 4652–4657 (2006). 16537404 10.1073/pnas.0600166103 PMC1450226

[18] Cairns, B. J., Timms, A. R., Jansen, V. A., Connerton, I. F. & Payne, R. J. Quantitative models of in vitro bacteriophage-host dynamics and their application to phage therapy. *PLoS Pathog.* **5**, e1000253 (2009). 19119417 10.1371/journal.ppat.1000253 PMC2603284

[19] Weitz, J. S. et al. A multitrophic model to quantify the effects of marine viruses on microbial food webs and ecosystem processes. *ISME J.* **9**, 1352–1364 (2015). 25635642 10.1038/ismej.2014.220 PMC4438322

[20] Sandhu, S. K., Bayliss, C. D. & Morozov, A. Y. How does feedback from phage infections influence the evolution of phase variation in campylobacter?. *PLoS Comput. Biol.* **17**, e1009067 (2021). 34125841 10.1371/journal.pcbi.1009067 PMC8224891

[21] Letarov, A. et al. Effect of chemical factors on natural biocontrol of the melioidosis agent by amp1-like bacteriophages in agricultural ecosystems. *Microbiology* **91**, 192–198 (2022).

[22] Egilmez, H. I., Morozov, A. Y. & Galyov, E. E. Modelling the spatiotemporal complexity of interactions between pathogenic bacteria and a phage with a temperature-dependent life cycle switch. *Sci. Rep.* **11**, 4382 (2021). 33623124 10.1038/s41598-021-83773-1 PMC7902855

[23] Suttle, C. A. & Chen, F. Mechanisms and rates of decay of marine viruses in seawater. *Appl. Environ. Microbiol.* **58**, 3721–3729 (1992). 16348812 10.1128/aem.58.11.3721-3729.1992 PMC183166

[24] Seeley, N. & Primrose, S. The effect of temperature on the ecology of aquatic bacteriophages. *J. Gen. Virol.* **46**, 87–95 (1980).

[25] Sinton, L. W., Hall, C. H., Lynch, P. A. & Davies-Colley, R. J. Sunlight inactivation of fecal indicator bacteria and bacteriophages from waste stabilization pond effluent in fresh and saline waters. *Appl. Environ. Microbiol.* **68**, 1122–1131 (2002). 11872459 10.1128/AEM.68.3.1122-1131.2002 PMC123754

[26] Wu, J. et al. Decay of coliphages in sewage-contaminated freshwater: uncertainty and seasonal effects. *Environ. Sci. Technol.* **50**, 11593–11601 (2016). 27709921 10.1021/acs.est.6b03916

[27] Haraga, A., West, T. E., Brittnacher, M. J., Skerrett, S. J. & Miller, S. I. Burkholderia thailandensis as a model system for the study of the virulence-associated type iii secretion system of burkholderia pseudomallei. *Infect. Immun.* **76**, 5402–5411 (2008). 18779342 10.1128/IAI.00626-08 PMC2573339

[28] Som-ard, J., Immitzer, M., Vuolo, F., Ninsawat, S. & Atzberger, C. Mapping of crop types in 1989, 1999, 2009 and 2019 to assess major land cover trends of the udon thani province, thailand. *Comput. Electron. Agric.* **198**, 107083 (2022).

[29] Thailand production (2023). https://ipad.fas.usda.gov/countrysummary/ (accessed 01 Jan 2022).

[30] Chuah, C. J., Tan, E. K., Sermswan, R. W. & Ziegler, A. D. Hydrological connectivity and *Burkholderia pseudomallei* prevalence in wetland environments: investigating rice-farming community’s risk of exposure to melioidosis in north-east thailand. *Environ. Monit. Assess.* **189**, 1–14 (2017). 10.1007/s10661-017-5988-1 28536911

[31] Dabral, P. & Murry, M. Z. Modelling and forecasting of rainfall time series using sarima. *Environ. Process.* **4**, 399–419 (2017).

[32] Box, G. E., Jenkins, G. M., Reinsel, G. C. & Ljung, G. M. *Time Series Analysis: Forecasting and Control* (Wiley, 2015).

[33] King, A. D., Karoly, D. J. & Henley, B. J. Australian climate extremes at 1.5 c and 2 c of global warming. *Nat. Clim. Chang.* **7**, 412–416 (2017).

[34] Henley, B. J. & King, A. D. Trajectories toward the 1.5 c paris target: modulation by the interdecadal pacific oscillation. *Geophys. Res. Lett.* **44**, 4256–4262 (2017).

[35] Newman, P. et al. What would have happened to the ozone layer if chlorofluorocarbons (cfcs) had not been regulated?. *Atmos. Chem. Phys.* **9**, 2113–2128 (2009).

[36] Egorova, T., Rozanov, E., Gröbner, J., Hauser, M. & Schmutz, W. Montreal protocol benefits simulated with ccm socol. *Atmos. Chem. Phys.* **13**, 3811–3823 (2013).

[37] Lindsey, R. & Dahlman, L. Climate change: Global temperature. *Climate. Gov* **16** (2020).

[38] Cheng, A. C. & Currie, B. J. Melioidosis: epidemiology, pathophysiology, and management. *Clin. Microbiol. Rev.* **18**, 383–416 (2005). 15831829 10.1128/CMR.18.2.383-416.2005 PMC1082802

[39] Wongbutdee, J., Jittimanee, J., Daendee, S., Thongsang, P. & Saengnill, W. Exploring the relationship between melioidosis morbidity rate and local environmental indicators using remotely sensed data. *Int. J. Environ. Res. Public Health* **21**, 614 (2024). 38791828 10.3390/ijerph21050614 PMC11121278

[40] Stouffer, R. J. & Wetherald, R. Changes of variability in response to increasing greenhouse gases. part i: Temperature. *J. Clim.* **20**, 5455–5467 (2007).

[41] Bathiany, S., Dakos, V., Scheffer, M. & Lenton, T. M. Climate models predict increasing temperature variability in poor countries. *Sci. Adv.* **4**, eaar5809 (2018). 29732409 10.1126/sciadv.aar5809 PMC5931768

[42] Suputtamongkol, Y. et al. The epidemiology of melioidosis in ubon ratchatani, northeast Thailand. *Int. J. Epidemiol.* **23**, 1082–1090 (1994). 7860160 10.1093/ije/23.5.1082

[43] Bhengsri, S. et al. Incidence of bacteremic melioidosis in eastern and northeastern Thailand. *Am. J. Trop. Med. Hyg.* **85**, 117 (2011). 21734135 10.4269/ajtmh.2011.11-0070 PMC3122354

[44] Hinjoy, S. et al. Melioidosis in Thailand: present and future. *Trop. Med. Infect. Dis.* **3**, 38 (2018). 29725623 10.3390/tropicalmed3020038 PMC5928800

[45] Møgelmose, S., Neels, K. & Hens, N. Incorporating human dynamic populations in models of infectious disease transmission: a systematic review. *BMC Infect. Dis.* **22**, 862 (2022). 36401210 10.1186/s12879-022-07842-0 PMC9673416

[46] Krug, E. G. Trends in diabetes: sounding the alarm. *Lancet* **387**, 1485–1486 (2016). 27061675 10.1016/S0140-6736(16)30163-5

[47] Mahikul, W. et al. Modelling population dynamics and seasonal movement to assess and predict the burden of melioidosis. *PLoS Negl. Trop. Dis.* **13**, e0007380 (2019). 31071094 10.1371/journal.pntd.0007380 PMC6529009

