# Supplementary material for: Modelling the effects of climate change on the interaction between bacteria and phages with a temperature-dependent lifecycle switch
Source: Sci Rep. 2025 Feb 21;15:6428. doi: 10.1038/s41598-025-89307-3 (PMC11845662; doi:10.1038/s41598-025-89307-3)
Supplement: Supplementary file 1 — Supplementary Information. [file 41598_2025_89307_MOESM1_ESM.pdf]

Supplementary Material for the article: Modelling the Effects of Climate Change on the Interaction between Bacteria and Phages with a Temperature-Dependent Lifecycle Switch.

Andrew Morozov[1,2], Areej Ageel[1], Anna Bates[1], and Edouard Galyov[1]
[1]University of Leicester, Leicester LE1 7RH, UK
[2] Institute of Ecology and Evolution, Russian Academy of Sciences, Leninsky pr. 33, Moscow 117071, Russia
*To whom correspondence should be addressed; E-mail: am379@leicester.ac.uk

Contents

1 Supplementary Material 1. Model equations and parameters. 2
1.1 Non-spatial model 2
1.2 Spatial model 3
1.3 Model parameters 4

2 Supplementary Material 2. Empirical investigation of infection of *B. pseudomallei* by the phage at high temperatures. 5

3 Supplementary Material 3. Observed annual average temperatures and UV index in Thailand. 5

4 Supplementary Material 4. Pairwise correlations between the external factors and the bacterial densities 5

5 Supplementary Material 5. Modelling effects of the carrying capacity on the forecast of bacteria-phage dynamics for the period 2024-2044 6

6 Supplementary Material 6. Modelling vertical distribution of bacteria and phage in soil. 8

7 Supplementary Material 7. Modelling effects of agricultural practices on bacteria-phage interactions in soil. 8
7.1 Modelling effects of soil mixing. 8
7.2 Modelling effects of agrochemicals on phage killing. 9

References 31

1 Supplementary Material 1. Model equations and parameters.

1.1 Non-spatial model

We implement a modification to the model by Eglimez et al. (Egilmez et al., 2018). The model consists of a system of four coupled differential equations for the densities of phage-free bacteria (S) susceptible to infection by the phage, bacteria infected by the phage in its lysogenic (I_1) and lytic (I_2) states, and free phages (P). The total density of the bacterial populations N is given by the sum $N = S + I_1 + I_2$.

$$
\begin{aligned}
\frac{dS(t)}{dt} &= \Big(\alpha(T(t)) - m(u(t))\Big)S(t)\Big[1 - \frac{N(t)}{C}\Big] - K_S S(t)P(t),\\
\frac{dI_1(t)}{dt} &= \Big(\overline{\alpha}(T(t)) - m(u(t))\Big)I_1(t)\Big[1 - \frac{N(t)}{C}\Big] + K_1(T(t))S(t)P(t) - \lambda_1(T(t))I_1(t),\\
\frac{dI_2(t)}{dt} &= K_2(T)S(t)P(t) + \lambda_1(T(t))I_1(t) - \lambda_2 I_2(t) - m(u(t))I_2(t),\\
\frac{dP(t)}{dt} &= -KN(t)P(t) - \mu(u(t))P(t) + b\lambda_2 I_2(t).
\end{aligned}
\tag{S1}
$$

In the above equations, $T(t)$ is the temperature at time t, $u(t)$ is ultraviolet index (UV index) of the intensity of solar radiation. The growth of susceptible bacteria is described by the logistic growth function, where α is the maximal per capita growth rate; C is the carrying capacity of the environment. The growth of lysogenised bacteria I_1 is described by the logistic function as well with the same carrying capacity, but with a maximal growth rate $\overline{\alpha}(T)$. Infection of bacteria by phages at low temperatures results in lysogeny; this is modelled via the term $K_s S(t)P(t)$. Transition from the lysogenic to the lytic cycle of infection occurs at warm temperatures, and it is described by the term $\lambda_1(T)I_1(t)$. Infection by the phage via the lytic cycle is modelled by $K_2(T)S(t)P(t)$. The mortality rate of infected bacteria due to lysis is described by the term $\lambda_2(T)I_2$. In this case, the lysis results in the release of b new phages. The loss of phage due to binding to bacteria is modelled by $KN(t)P(t)$. The time-dependent parameters $\mu(u(t))$ and $m(u(t))$ are the natural mortality (deactivation) rates of phages and bacteria, respectively; these terms depend on the UV index $u(t)$.

The maximal bacterial growth rates $\alpha(T)$ and $\overline{\alpha}(T)$ are parameterised via

$$\alpha(T) = \exp(-\frac{(T - T_0)^2}{2\sigma^2})\alpha_{\max}, \tag{S2}$$

$$\overline{\alpha}(T) = \alpha_{\max}\exp\big(-\frac{(T - T_0)^2}{2\sigma^2}\big)\frac{T_1^n}{T_1^n + T^n}, \tag{S3}$$

Here $T_0 = 38.2$ °C is the optimal temperature for the bacterial growth; $T_1 = 34.8$ °C is the temperature corresponding to the switch between the lytic and the lysogenic cycles; $\alpha_{\max} = 23$ day^{-1} is the maximal possible growth, $\sigma = 9.1$ °C describes the decay of growth with temperature T (Chen, Chen, Kao, & Chen, 2003; Egilmez et al., 2018).

The temperature dependence of the adsorption constants $K_1(T)$, $K_2(T)$ are parameterised as:

$$
\begin{aligned}
K_1(T) &= \frac{T_1^n}{T_1^n + T^n}K_S\frac{T_{cr}^m}{T_{cr}^m + T^m},\\
K_2(T) &= \frac{T^n}{T_1^n + T^n}K_S\frac{T_{cr}^m}{T_{cr}^m + T^m},
\end{aligned}
\tag{S4}
$$

where K_S is the maximal phage adsorption constant ($K_S = \epsilon K$ where $\epsilon = 0.3$ is the adsorption efficiency). T_{cr} is the critical temperature, where the phage becomes unable to infect bacteria. The critical temperature $T_1 = 35$ °C corresponds to the switch between the lytic and the lysogenic cycles (Egilmez et al., 2018; Egilmez, Morozov, & Galyov, 2021). The above parameterisations are discussed in the main text of the paper (see 'Modelling approach' in the Methods section for details)

The temperature dependence of the transition rate from lysogenic to lytic cycle $\lambda_1(T)$ is parameterised as (see (Egilmez et al., 2018) for detail)

$$\lambda_1(T) = \frac{T^n}{T_1^n + T^n}\lambda_{1\max},$$

where $\lambda_{1_{\max}}$ is the maximal transition rate.

The realistic values of model parameters are listed in Table 1 of Section SM1.3. The temperature values $T(t)$ are taken from historic records and are described in the main text. Numerical simulations of ODEs were performed using the Runge-Kutta method of order 4. The initial conditions for the species densities were taken as $S(t=0) = 0.5 \times 10^5$cell/ml; $I_1(t=0) = 1.5 \times 10^6$cell/ml; $I_2(t=0) = 0.5 \times 10^6$cell/ml; $P(t=0) = 2.5 \times 10^8$ pfu/ml. Note that we ran simulations from $t = 0$ over the period of first 3 years, corresponding to the meteorological conditions (the temperature and UV index) of the first considered year (2009) for the province under consideration, before proceeding to further years of the same province. This was done to avoid possible influence of transient dynamics.

1.2 Spatial model

The equations for bacteria-phage interaction in soil are summarised in the following reaction-diffusion model

$$\begin{aligned}
\frac{\partial S(t,h)}{\partial t} &= D_b\frac{\partial^2 S(t,h)}{\partial h^2} + \alpha(T(t,h)S(t,h)\Big[1 - \frac{N(t,h)}{C(h)}\Big] - K_S S(t,h)P(t,h),\\
\frac{\partial I_1(t,h)}{\partial t} &= D_b\frac{\partial^2 I_1(t,h)}{\partial h^2} + \overline{\alpha}(T(t,h))I_1(t,h)\Big[1 - \frac{N(t,h)}{C(h)}\Big] + K_1(T(t,h))S(t,h)P(t,h) - \lambda_1(T(t,h))I_1(t,h),\\
\frac{\partial I_2(t,h)}{\partial t} &= D_b\frac{\partial^2 I_2(t,h)}{\partial h^2} + K_2(T(t,h))S(t,h)P(t,h) + \lambda_1(T(t,h))I_1(t,h) - \lambda_2 I_2(t,h),\\
\frac{\partial P(t,h)}{\partial t} &= D_P\frac{\partial^2 P(t,h)}{\partial h^2} - KN(t,h)P(t,h) - \mu_0 P(t,h) + b\lambda_2 I_2(t,h).
\end{aligned} \tag{S5}$$

The variation of the temperature $T(t,h)$ across the soil is described by the standard heat equation

$$\frac{\partial T(t,h)}{\partial t} = D_h\frac{\partial^2 T(t,h)}{\partial h^2}, \tag{S6}$$

where D_h is the heat transfer coefficient.

For the above model, we assume the zero-flux boundary condition for all biotic components (bacteria and phage) at $h = 0$ and $h = H$. For the temperature, we consider Dirichlet boundary conditions such that $T(t,0) = T_s(t)$ and $T(t,H) = T_H$, where $T_s(t)$ is the surface temperature and T_H is a constant temperature in deeper soil layers.

The carrying capacity C of the bacteria varies with the depth of the soil according the following curve(Egilmez et al., 2021):

$$C(h) = (C_{\mathrm{surf}} - C_0)\exp(-Bh^2) + C_0, \tag{S7}$$

where C_{surf} is the maximal number, the coefficient B determines the rate of decrease of the bacterial abundance with depth, C_0 describes background bacterial at large depths (note that bacteria can survive even at large depths, for example, at $h = 100$ cm). In the spatial model, the mortality rate of phage μ_0 in the soil does not depend on the UV index.

In our numerical simulations, we use both explicit and implicit numerical schemes. We take a 0.1 cm spatial step size to get a proper resolution. We separately compute the heat equation to define $T(t)$ with a smaller time resolution and then apply the temperature obtained to model bacteria-phage interactions for a larger time resolution (for example, $\Delta t \cong 7 \times 10^{-5}$ day). We compute the average densities of the species (both in terms of spatial and temporal averaging) using a numerical right Riemann sum. The accuracy of our numerical simulation was verified by reducing both time and space steps and comparing the results obtained.

Table S1: Parameters used in the mathematical models (non-spatial and spatial) along with their units and ranges.

Symbol	Meaning	Unit	Range	Default Value
D_b	Bacteria diffusion coefficient in soil (spatial model)	$cm^2\ day^{-1}$	$10^{-12} - 10^{-3}$	10^{-7}
D_P	Phages diffusion coefficient in soil (spatial model)	$cm^2\ day^{-1}$	$10^{-12} - 10^{-3}$	10^{-5}
D_h	Heat diffusion coefficient in soil (spatial model)	$cm^2\ day^{-1}$	–	67
$\alpha_{\max}$	Maximum growth rate of bacteria	day^{-1}	$19 - 27$	23
C	Bacteria carrying capacity in the surface water (non-spatial model)	*cell*/ml	$1 \times 10^6 - 5 \times 10^7$	2×10^6
C_{surf}	Bacteria carrying capacity near the surface (spatial model)	*cell*/ml	$1 \times 10^6 - 1 \times 10^7$	1×10^6
C_0	Bacteria carrying capacity at large depths (spatial model)	*cell*/ml	$1 \times 10^4 - 1 \times 10^7$	1×10^6
$\sqrt{B}$	Inverse characteristic length of $C(h)$ (spatial model)	cm^{-2}	–	7.5×10^{-4}
K	Phage adsorption rate	$ml^{-1}day^{-1}$	$5 \times 10^{-8} - 5 \times 10^{-7}$	1×10^{-7}
K_S	Effective per bacteria contact rate	$ml^{-1}day^{-1}$	–	$\epsilon \times 10^{-7}$
ϵ	Adsorption efficiency	–	–	0.3
$\lambda_{1\max}$	Maximum lysogenic process rate	day^{-1}	$19.1 - 27.2$	23
λ_2	Constant lysis rate	day^{-1}	–	20
b	Phage burst size	–	$50 - 200$	105
T_0	Optimum temperature for growth and lysis	°C	$35.6 - 50.6$	38.2
T_1	Optimum transition temperature	°C	$34.81 - 34.84$	34.8
T_{cr}	Critical temperature, where phages stop infecting bacteria	°C	$39 - 40$	40
σ	Standard deviation of growth rate	°C	$6.7 - 17.4$	9.1
μ_c	Background mortality of phages (non-spatial model)	day^{-1}	-	0.1
Y_0, k	Parameters in $\mu(u)$ and $m(u)$ (non-spatial model)	day^{-1} for Y_0	-	$k = 0.24$; $Y_0 = 0.67$
μ_0	Mortality rate of phages (spatial model)	day^{-1}	$0.1 - 15$	3
n, m	Transition width parameters	–	$53.7 - 56.3$	55

The initial conditions for the species densities for the spatial model were taken as: $S(t = 0, h) = 0.6C(h)$; $I_1(t = 0, h) = 0.4C(h)$; $I_2(t = 0, h) = 1 \times 10^4$ cell/ml; $P(t = 0, h) = 1 \times 10^8$ pfu/ml. Note that we ran simulations from $t = 0$ over the period of first 3 years, corresponding to the meteorological conditions (the temperature and UV index) of the first considered year (2009) for the province under consideration, before proceeding to further years of the same province. This was done to avoid possible influence of transient dynamics.

1.3 Model parameters

The parameter values for both spatial and non-spatial models are provided in Table 1. The choice of parameters for the given system of *B. pseudomallei*-phage interaction is discussed in the previous papers (Egilmez et al., 2018, 2021). The estimate for the critical temperature T_{cr} (where the phage is unable to infect bacteria) is based on our empirical data, which are provided in SM2.

Temperature (° C)	Rep 1	Rep 2	Rep 3	Average
37	1.30E+07	1.00E+07	1.00E+07	1.10E+07
38	2.00E+07	2.00E+07	2.00E+07	2.00E+07
39	1.00E+07	4.00E+07	9.00E+07	4.67E+07
40	3.00E+03	9.00E+03	9.00E+03	7.00E+03
41	Neat	Neat	Neat	-

Table S2: Effects of temperature on phage infection. For all temperatures, the values of Pfu/ ml are shown after incubation over 24h.

2 Supplementary Material 2. Empirical investigation of infection of *B. pseudomallei* by the phage at high temperatures.

To understand the effect of high temperatures on the infection of *B. pseudomallei* by AMP1 phage, we performed a phage spot test experiment in a laboratory. We used *B. thailandensis* as a proxy of *B. pseudomallei. B. thailandensis* E264 was a provided by Prof Don Woods, Department of Microbiology and Infectious Diseases, Faculty of Medicine, University of Calgary Health Sciences Centre, Canada (see the related publication for details (Brett, Deshazer, & Woods, 1997)). The phage AMP1 was previously isolated and characterised by Dr Ed Galyov, at the University of Leicester, UK (see the related publications for details (Gatedee et al., 2011; Shan et al., 2014)).

Five plates were incubated at temperatures ranging from 37 $°C$ to 41 $°C$ overnight. Pfu/ ml for each temperature was calculated the next day and EOP determined. Technical details of the conducted experiment are provided on the main text of the paper (see Methods).

The results of bacteria plating with phages (after 24h) at different temperatures and at different dilutions are presented in Table S2 and Figure 1 in the main text. Overall, our experiment suggests that AMP1 cannot exhibit lytic behaviour at temperatures exceeding 40 $°C$.

Overall, our experiment suggests that AMP1 cannot exhibit lytic behaviour at temperatures exceeding 40 $°C$.

3 Supplementary Material 3. Observed annual average temperatures and UV index in Thailand.

In this section, we present the annual average temperatures and UV index based on meteorological observation in the eight considered provinces of Thailand for the period from 2009-2024, see Figure S1. The names of the considered provinces are: Sa Kaeo, Nakhon Phanom, Bangkok, Mukdahan, Ubon Ratchathani, Si Sa Ket, Buri Ram, and Roi Et. Details on data collection and the geographic position of provinces are provided in the main text (Section 'Methods').

4 Supplementary Material 4. Pairwise correlations between the external factors and the bacterial densities

In this section, we calculate Pearson's pairwise correlations between the annual average temperature, UV index, total annual number of hot hours 'HH' within the year where the temperature $T > 35$ $°C$ (all three mentioned factors are taken from meteorological data), and the annual average densities of susceptible bacteria S, predicted by the non-spatial mathematical model. The results are presented in Figure S2. The corresponding p-values of the estimates of the correlation coefficients are provided below in Table S3.

Overall, Figure S2 demonstrates a high (and statistically significant) level of the pairwise positive correlations between the considered three environmental components and the predicted bacterial density S.

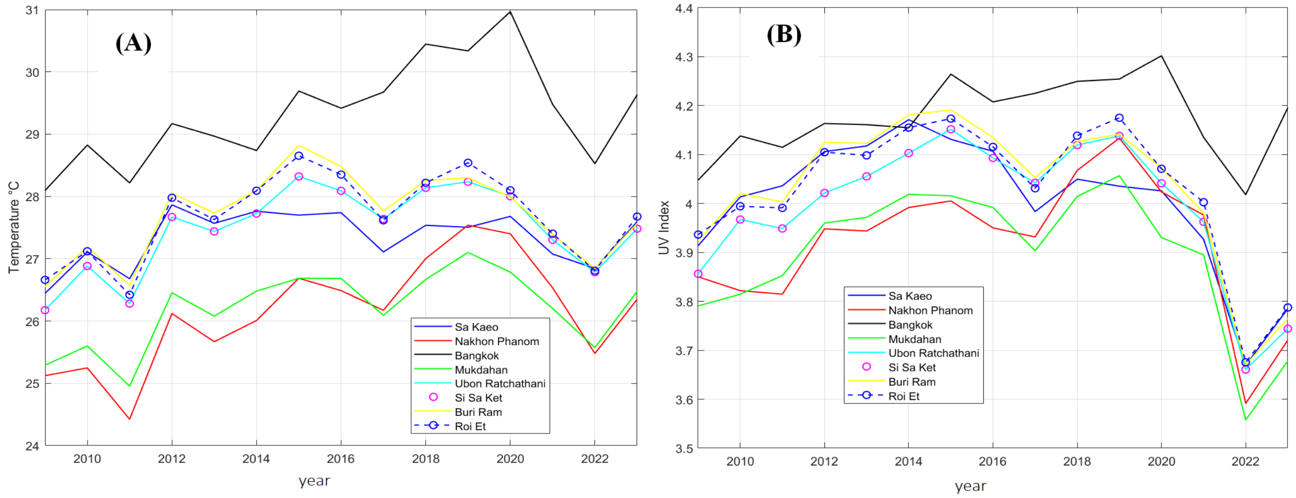

Figure S1: (A) The annual average temperatures and (B) the annual average UV index in the eight selected provinces in Thailand. The names of provinces are indicated in the figure label.

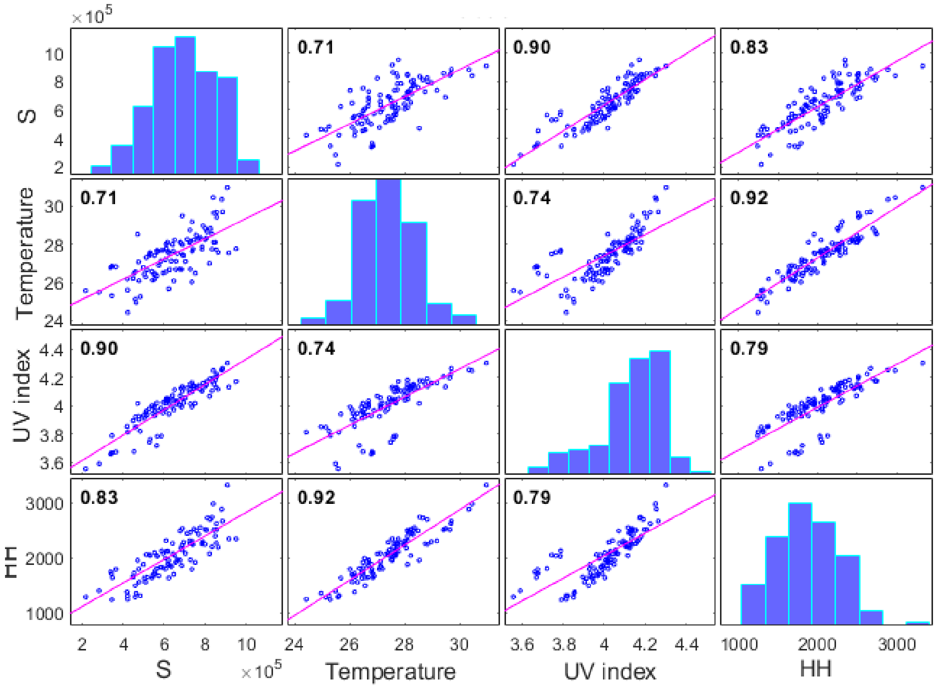

Figure S2: Pairwise correlations between the values of the annual average densities of susceptible bacteria S (predicted by the non-spatial model), temperature, UV index, and total annual number of hot hours within the year where the temperature $T > 35\ ^{\circ}C$ (denoted by 'HH'). All eight considered provinces in Thailand are included in the simulation; the time period covers 2009-2023. The values of bacterial densities are obtained using the non-spatial model. The correlation coefficients are listed in the figure. The unit of the densities of bacteria is cell/ml and for phages is pfu/ml.

5 Supplementary Material 5. Modelling effects of the carrying capacity on the forecast of bacteria-phage dynamics for the period 2024-2044

In this section, we briefly explore effects of variation of the carrying capacity C of the bacteria on the outcome of *B. pseudomallei*-phage interaction over the period of forecast of 2024-2024. We considered higher values

	Temperature	UV index	Hot Hours (HH)	Bacterial density, S
**Temperature**	1	1.0E-19	5.0E-44	3.4E-31
**UV index**	1.0E-19	1	2.5E-22	2.0E-50
**Hot Hours (HH)**	5.0E-44	2.5E-22	1	1.5E-26
**Bacterial density, S**	3.4E-31	2.0E-50	1.5E-26	1

Table S3: Calculated p-values of the estimates of the Pearson's pairwise correlation coefficients shown in Figure S2.

of C, which would signify an eventual enrichment of agricultural soil due to extensive use of fertilises. In this study, we considered four different values of the carrying capacity given by $C = 5 \times 10^6$, $C = 1 \times 10^7$, $C = 2 \times 10^7$, and $C = 5 \times 10^7$ (measured in cell/ml). We keep other model parameters to be the same as in the main text. We forecast *B. pseudomallei*-phage interaction in the the Nakhon Phanom province, based on a non-spatial model. The results of our simulation are shown in Figure S3, S4 and S5.

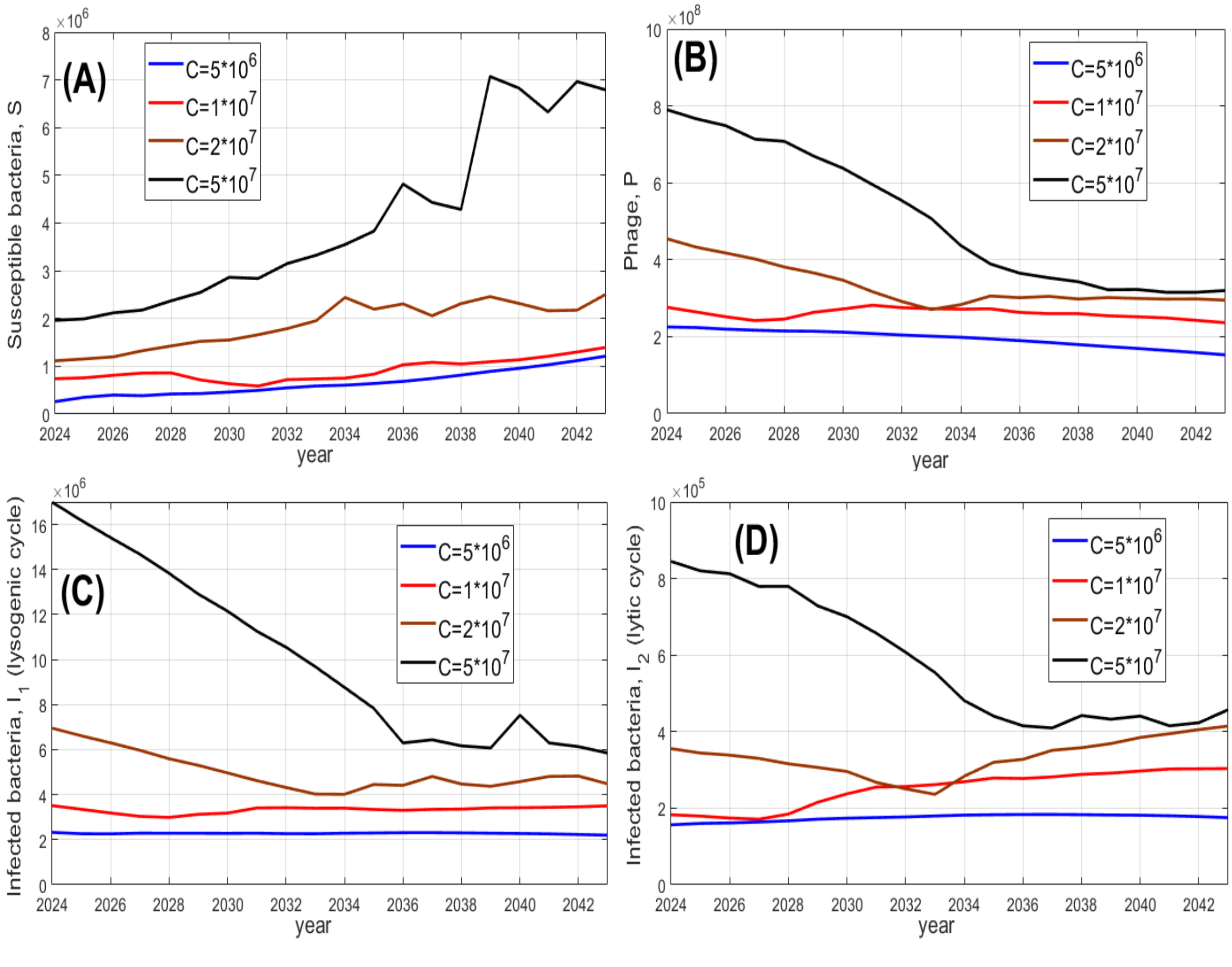

Figure S3: Predicted outcomes of bacterial-phage interactions for years 2024-2044 for the Nakhon Phanom province (based on the non-spatial model). In each panel, the annually average values of species densities (S, P, I_1, I_2) are plotted for different years. The predictions are obtained for 4 different values of the carrying capacity C (measured in cell/ml). The unit of the densities of bacteria is cell/ml and for phages is pfu/ml. The other model parameters are the same as in Figure 7 of the main text.

Figure S3 plots annual densities of bacteria and the phage for the considered period of forecast. For simplicity, we considered only a single scenario of UV index trend, which is a gradual increase by 3 units over next 20 years (this corresponds to UV(2) in Figure 7 of the main text). One can see from the figure that higher values of C (high level of enrichment of soil) correspond to higher levels of bacteria and phage in the system, as compared, for example, to the default value of $C = 2 \times 10^6$ cell/ml, considered in the main text. The resultant global change of the climate is predicted to cause an increase in the density of susceptible bacteria S and a decline of the density of phage. These changes are particularly amplified at higher values of carrying capacity (e.g. $C = 5 \times 10^7$ cell/ml).

We also explored alteration in the seasonal and monthly dynamics of bacteria-phage interactions, caused by the global change. Two illustrative examples are shown in Figures S4 and S5 which plots average daily densities of S and P, respectively, across each year over the considered period of forecast. The graphs are constructed for $C = 5 \times 10^6$ and $C = 5 \times 10^7$ cell/ml. One can see that an increase in the temperature and the UV index is predicted to result in the occurrence of more outbreaks of bacteria density across the year, which are explained by the fact of weakening of control of the pathogen by the phage. The phage density is predicted to exhibit a decline due to an increase of mortality (due to a higher UV level) and higher temperatures (due to inability of the phage to infect bacteria at hot temperatures). Comparing two different values of the carrying capacity C indicates that for high levels of enrichment (see panel (B), $C = 5 \times 10^7$), a regular pattern of outbreaks of susceptible bacteria (bacterial 'bloom') near the start period becomes altered, and by the end of the period there are irregular frequent outbreaks of bacteria densities across the year. The mentioned transition between patterns is less pronounced for lower values of carrying capacity (see panel (A) $C = 5 \times 10^6$).

6 Supplementary Material 6. Modelling vertical distribution of bacteria and phage in soil.

Here we show how patterns of daily variation of the vertical distribution of the density of susceptible bacteria S and that of free phage P across the soil change from day to day over a one month period (in the Nakhon Phanom province). We also present daily temperature variation at different depths, as predicted by the model. The results are obtained using the spatial model from section 1.2 of the supplementary material. Figures S6-S11 show profiles of densities and temperatures corresponding to May (years 2017, 2021), whereas Figures S12-S17 show the corresponding graphs for September (years 2017, 2021). For May, we show the distribution of bacteria/phage up to 100cm, whereas for September, we we limit ourselves by the upper 20cm. This is technically required for the further comparison of vertical profiles of bacteria and free phage after application of agricultural practices (see next Section), such as soil mixing, and phage removal.

7 Supplementary Material 7. Modelling effects of agricultural practices on bacteria-phage interactions in soil.

7.1 Modelling effects of soil mixing.

Agricultural activities, such as plowing, shoveling, and digging, lead to mixing of the soil. Here we model how mixing of soil would affect bacteria-phage interactions. We assume that mixing of soil occurs on the 1st of May each year.

In the model, we mimic soil mixing by making the densities of all species to be homogeneous within the top 40cm of soil (the resultant density after mixing is the spatial average density within the same depth just prior mixing). Some examples of simulations with soil mixing are provided in Figures S18-S21, which are constructed for the Nakhon Phanom province in Thailand for years 2017 and 2021.

We found that the density of susceptible bacteria and that of free phage near the surface (e.g. top 15-20 cm) recovers quickly and reaches similar levels to those without mixing (this was done by visual comparing figures from the previous section for the same month and the year). The period of relaxation to the non-mixed system is approximately one week (up the the depth of 20cm). On the other hand, we also found that soil mixing has a significant impact on bacteria presence at depths between 20cm and 40cm. In particular,

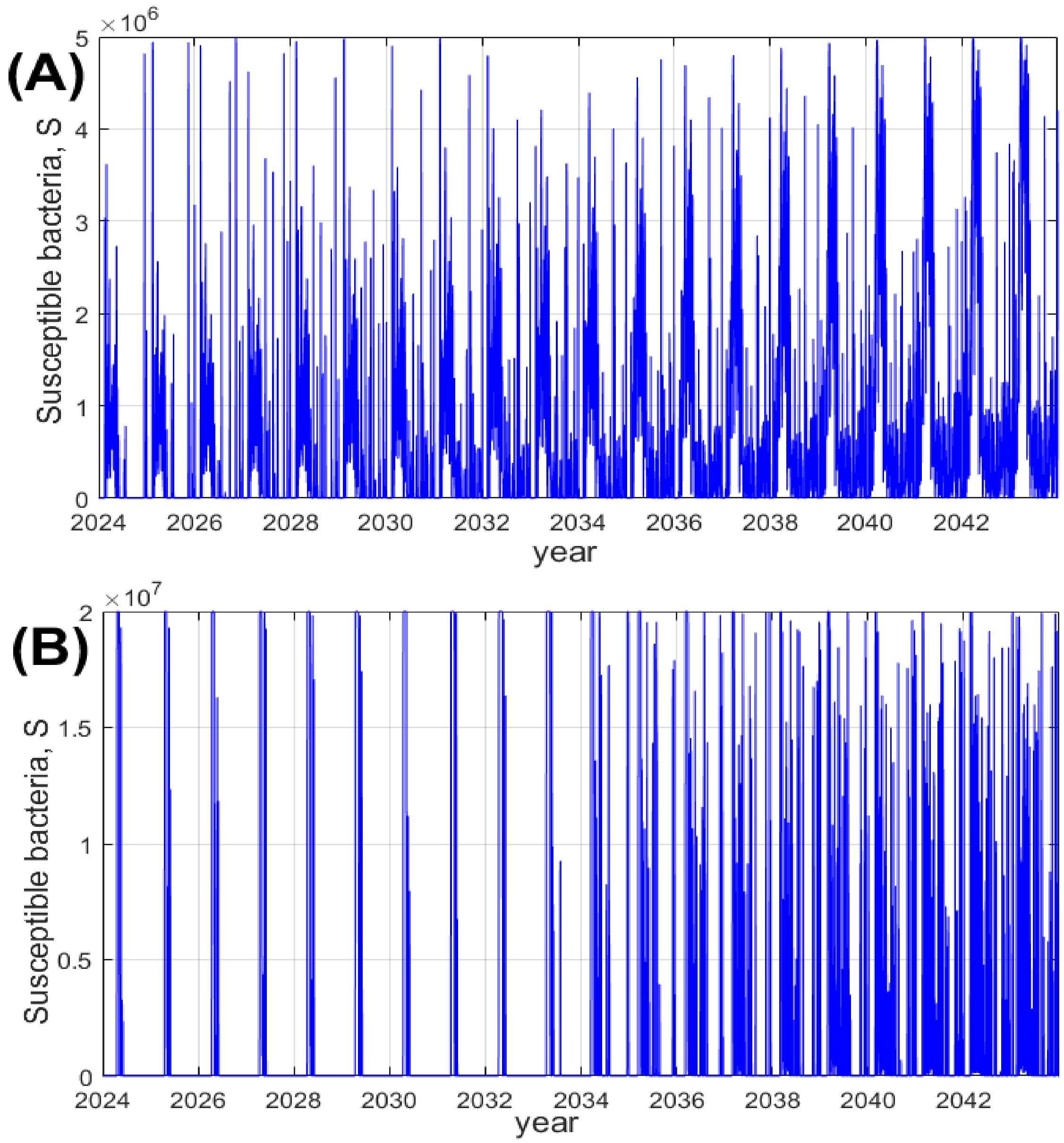

Figure S4: Predicted dynamics of the bacterial density for years 2024-2044 for the Nakhon Phanom province (based on the non-spatial model). The graphs show daily average values of S for each year within the period of forecast. Panels (A) and (B) correspond to the carrying capacities of $C = 5 \times 10^6$ and $C = 5 \times 10^7$ (measured in cell/ml), respectively. The other model parameters are the same as in Figure 7 of the main text. Bacterial density is measured in cell/ml.

mixing of soil would eliminate the peak of the density of susceptible bacteria, which occurs in the system without mixing at depths of approximately 30cm (see Figure 10 in the main text).

7.2 Modelling effects of agrochemicals on phage killing.

We also modelled the negative influence of agrochemicals on the efficiency of the control of pathogenic bacteria by the phage (some agrochemical kills free phages (Letarov et al., 2022)). To model this phenomenon, we eliminated the free phage in the top layer of the soil (from the surface up to 5cm) in the spatial model on September 1st. The outcomes of the phage removal are shown in Figures S22-S25, constructed for two

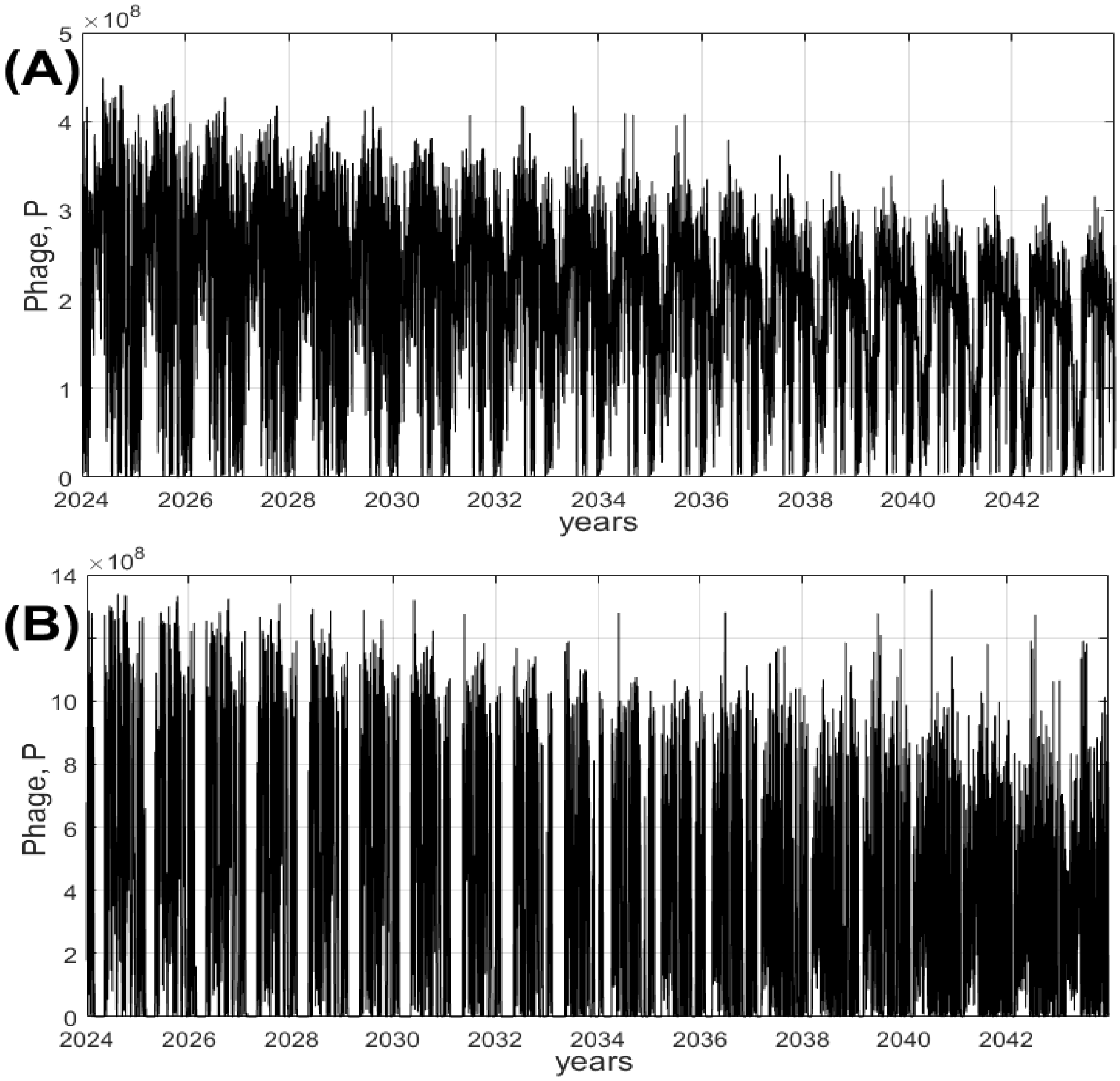

Figure S5: Predicted dynamics of the free phage density for years 2024-2044 for the Nakhon Phanom province (based on the non-spatial model). The graphs show daily average values of P for each year within the period of forecast. Panels (A) and (B) correspond to the two different values carrying capacities bacteria: $C = 5 \times 10^6$ and $C = 5 \times 10^7$ (measured in cell/ml), respectively. The other model parameters are the same as in Figure 7 of the main text. Phage density is measured in pfu/ml.

different years (2017, 2021). One can see that the effect of phage removal can vary from year to year. For example, the impact of phage killing in year 2017 is observed within 2 weeks, whereas in year 2021, the relaxation time is approximately 3 weeks. This can be found by a visual comparison of Figures S22-S25 with the corresponding figures in the absence of phage killing (see Figures S12-S15). We must stress that the difference in the time of relaxation of the system to the original state after phage killing in the top 5cm largely depends on the temperature, which can be seen by comparison of the temperature dynamics which can be seen from Figures S16-S17.

Importantly, in both shown examples in Figures S22-S25, removal of phages produced a pronounced outbreak of the density of the susceptible bacteria S, which signifies a higher risk of infection by the pathogen.

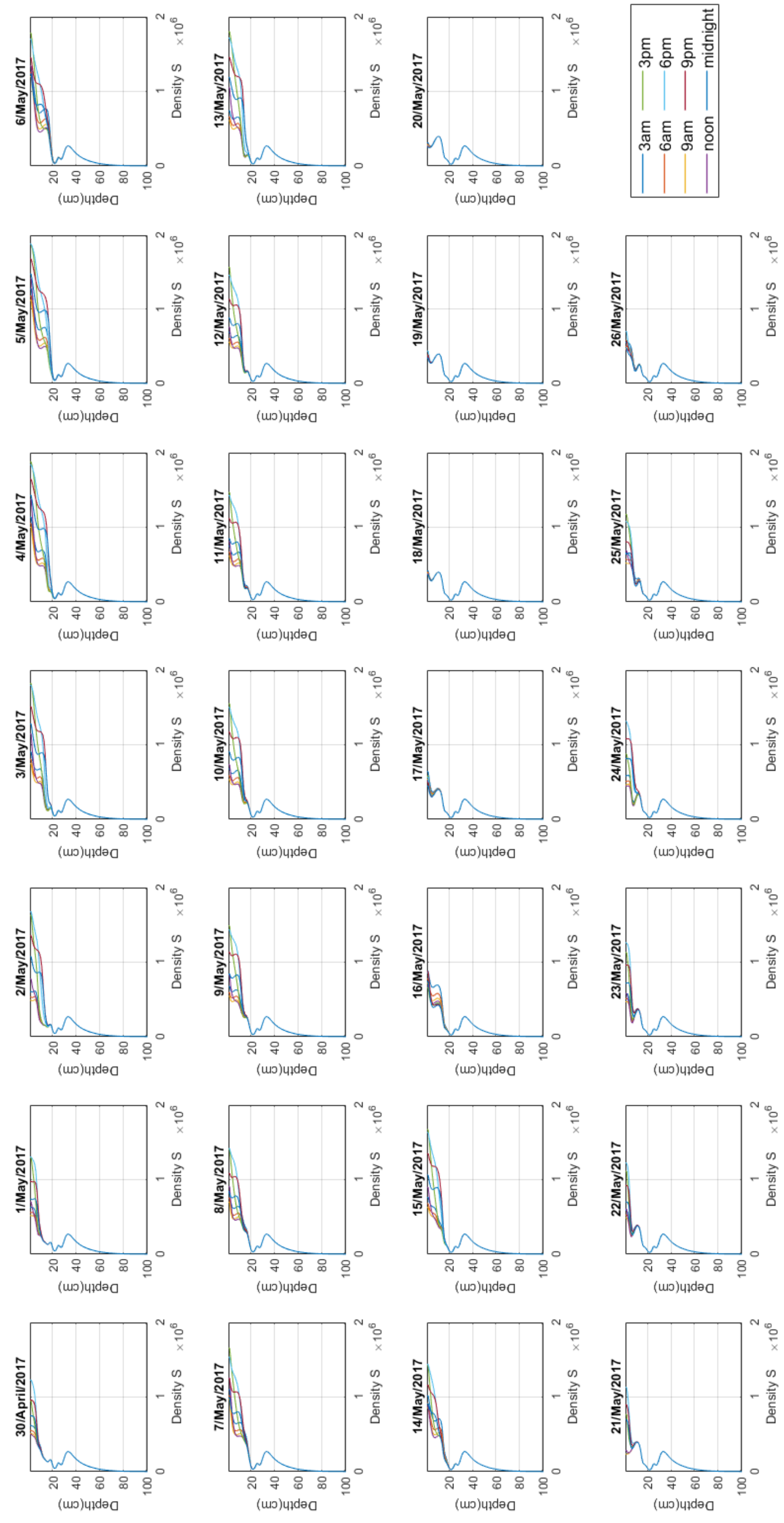

Figure S6: Dynamics of vertical distribution of susceptible bacteria S in soil (up to 100cm) predicted by the spatial model in May 2017 in the Nakhon Phanom province in Thailand. Bacterial density is measured in cell/ml.

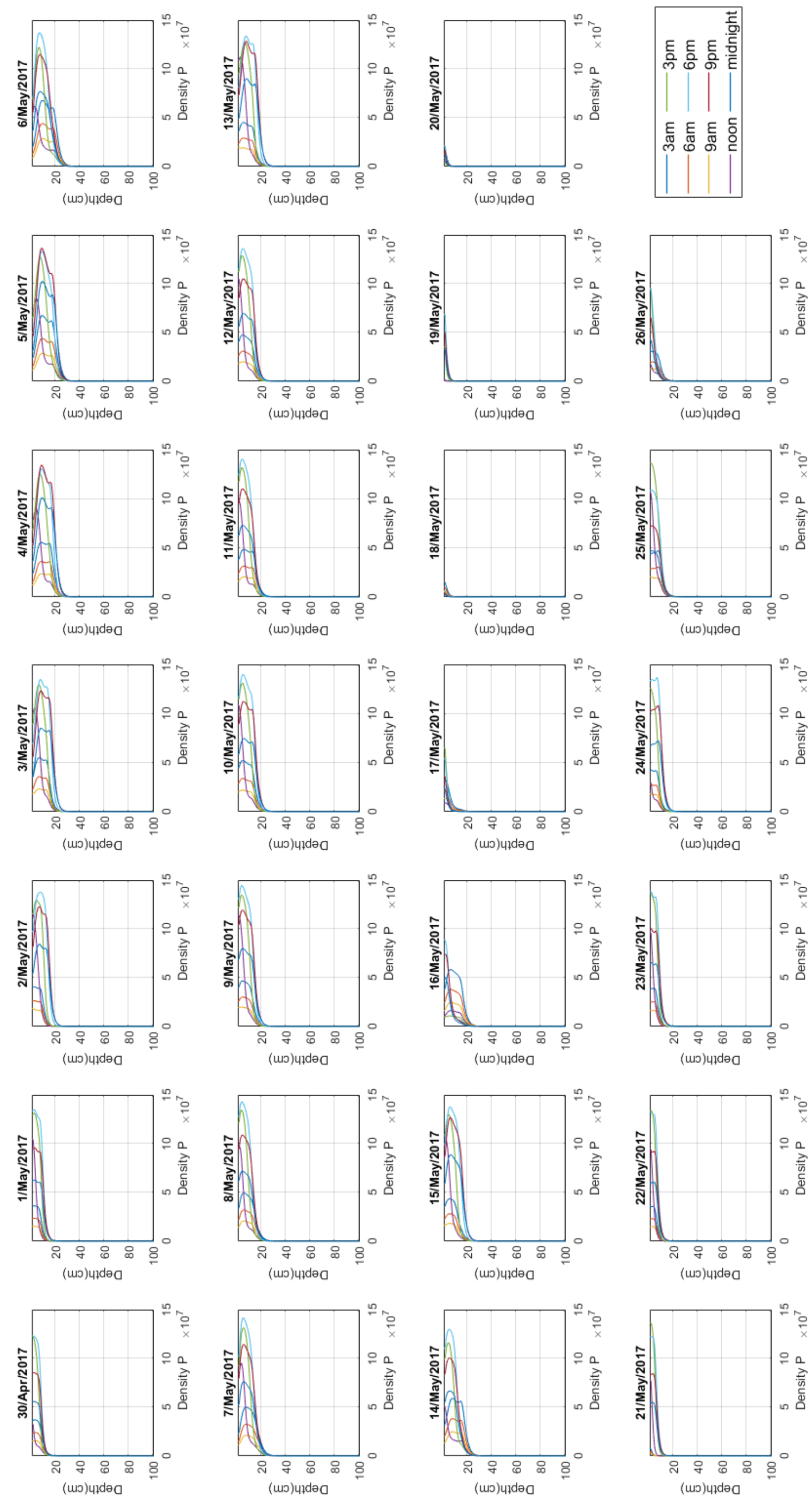

Figure S7: Dynamics of vertical distribution of free phage P in soil (up to 100cm) predicted by the spatial model in May 2017 in the Nakhon Phanom province in Thailand. Phage density is measured in pfu/ml.

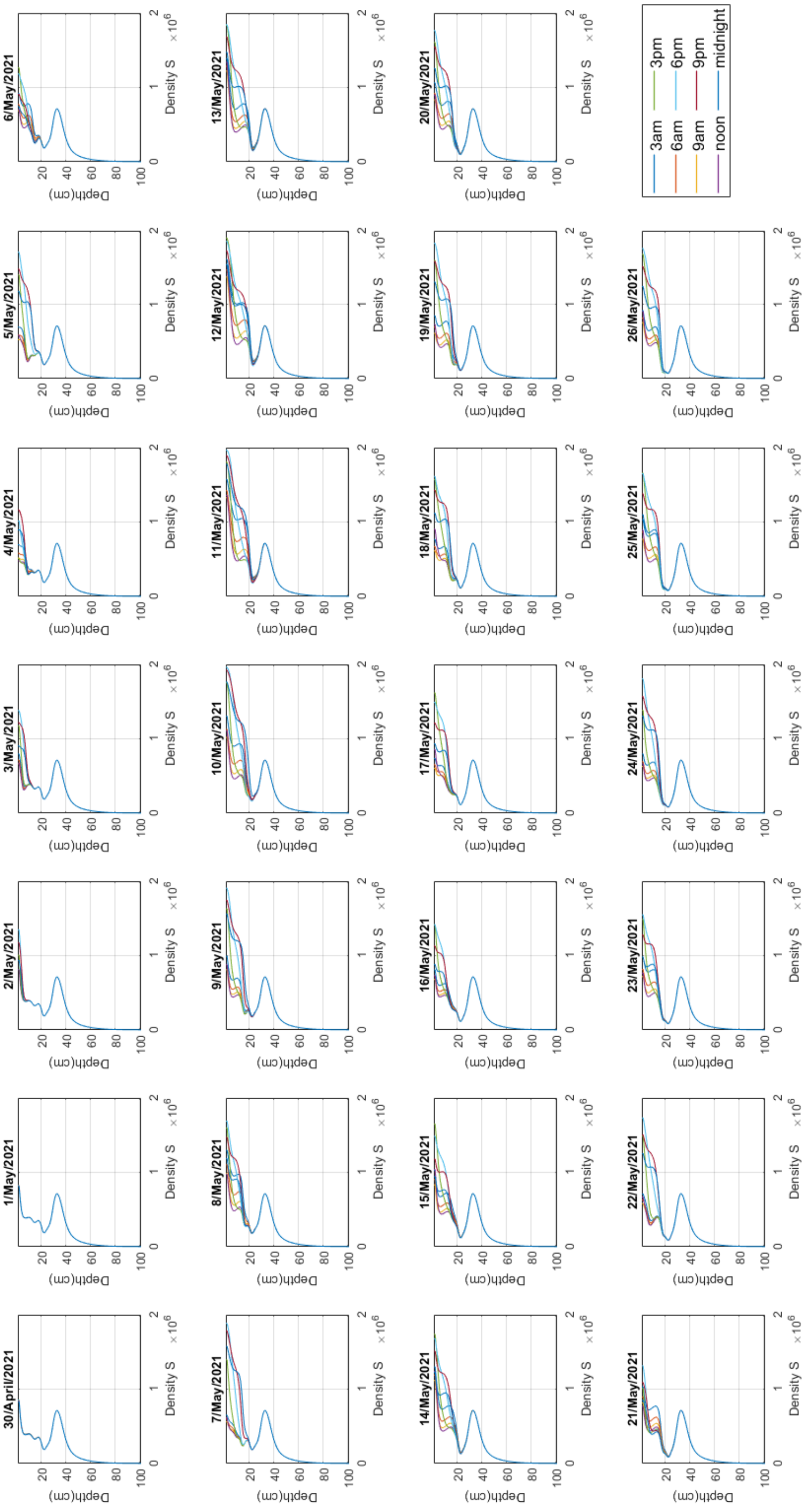

Figure S8: Dynamics of vertical distribution of susceptible bacteria S in soil (up to 100cm) predicted by the spatial model in May 2021 in the Nakhon Phanom province in Thailand. Bacterial density is measured in cell/ml.

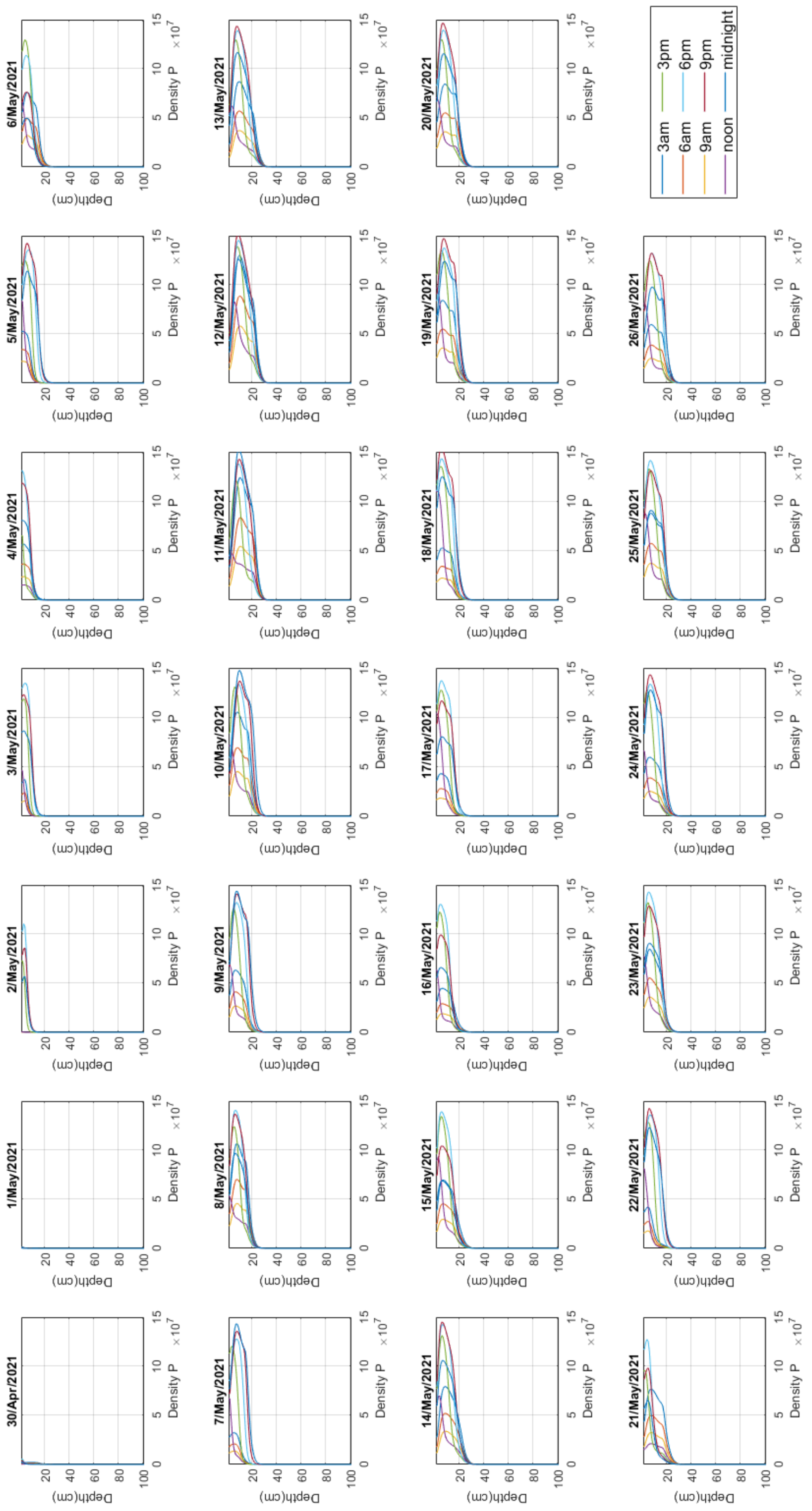

Figure S9: Dynamics of vertical distribution of free phage P in soil (up to 100cm) predicted by the spatial model in May 2021 in the Nakhon Phanom province in Thailand. Phage density is measured in pfu/ml.

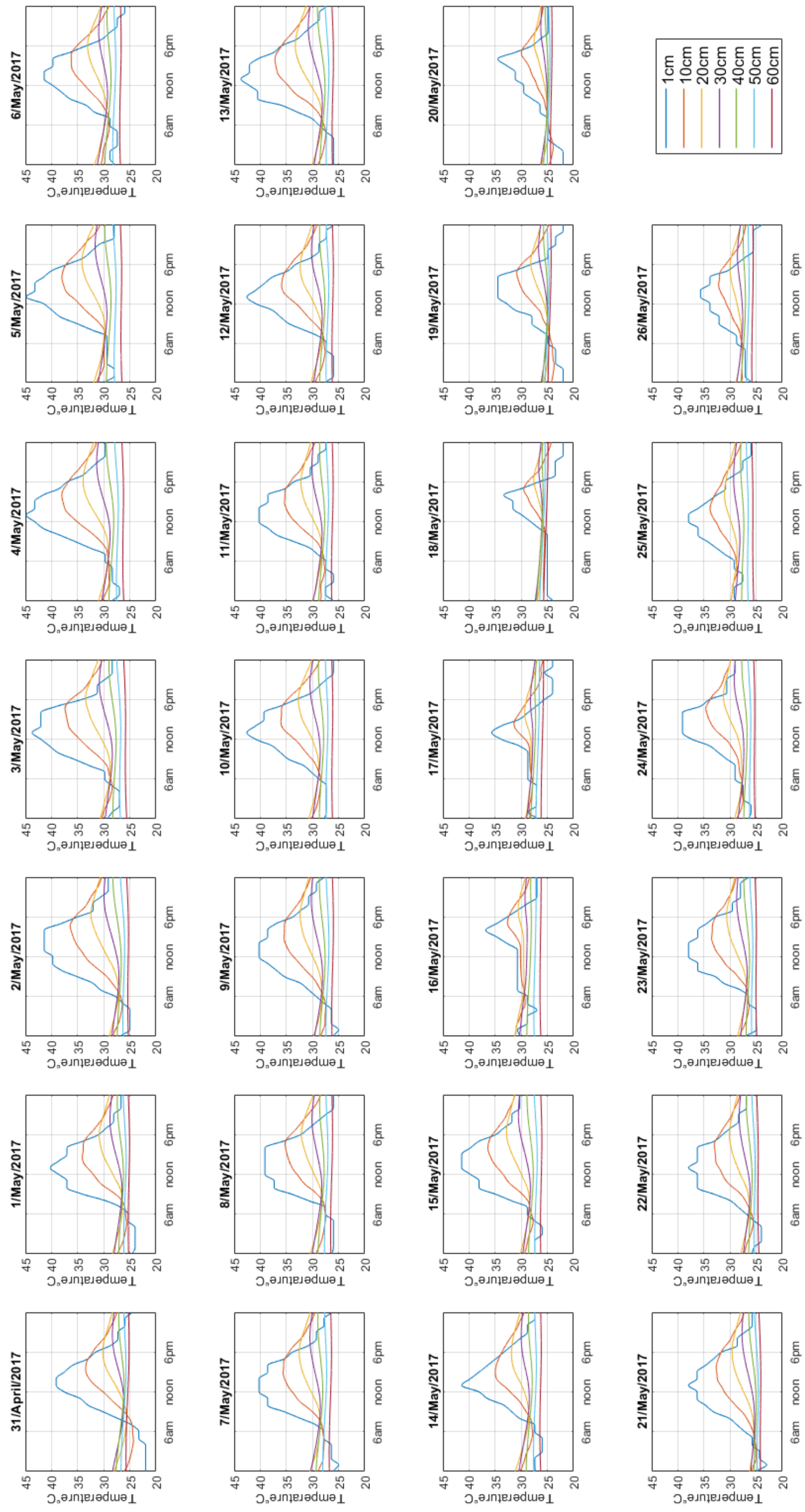

Figure S10: Daily temperature fluctuation predicted by the spatial model in May 2017 in the Nakhon Phanom province in Thailand. Each curve corresponds to a particular depth.

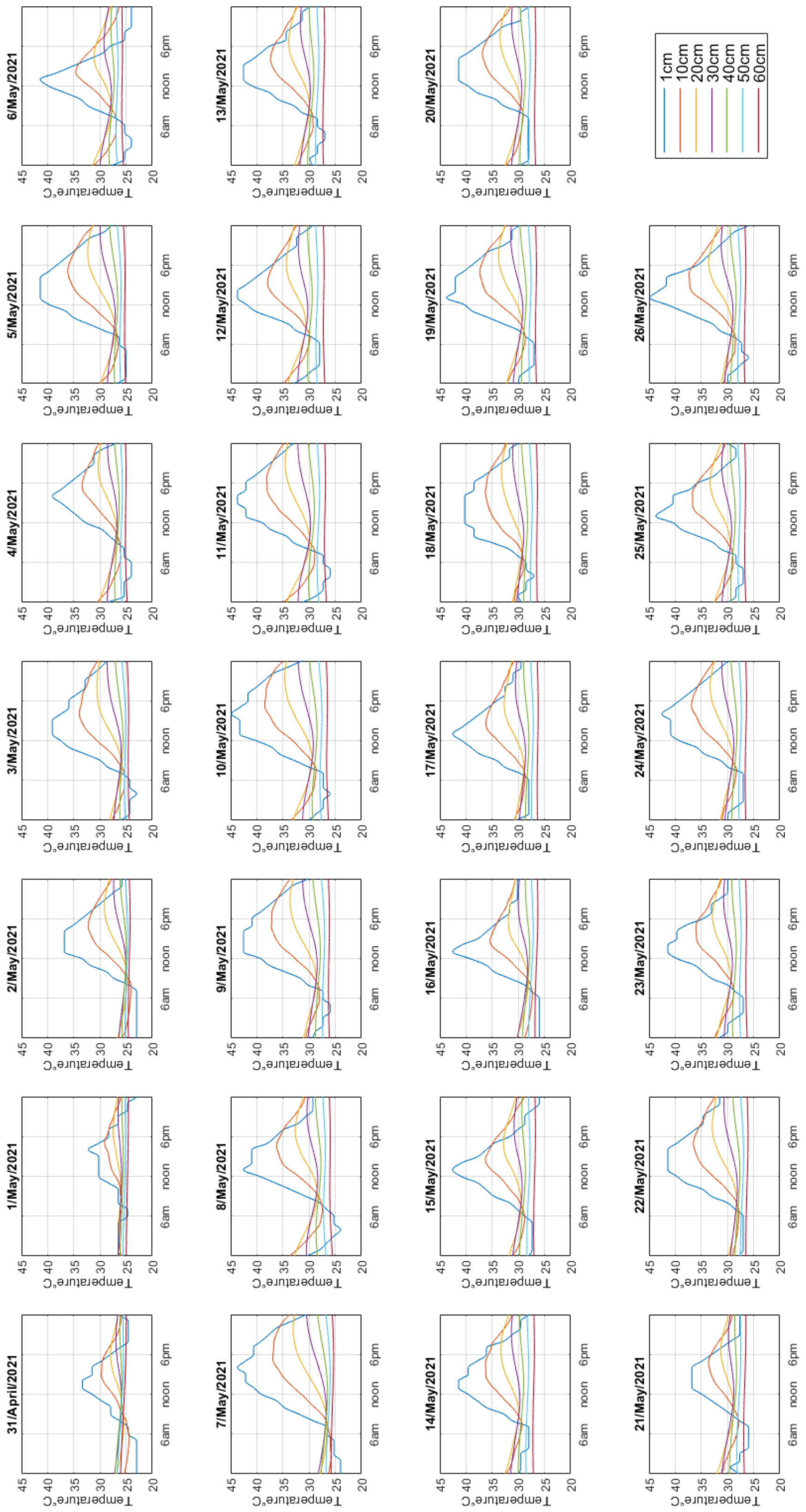

Figure S11: Daily temperature fluctuation predicted by the spatial model in May 2021 in the Nakhon Phanom province in Thailand. Each curve corresponds to a particular depth.

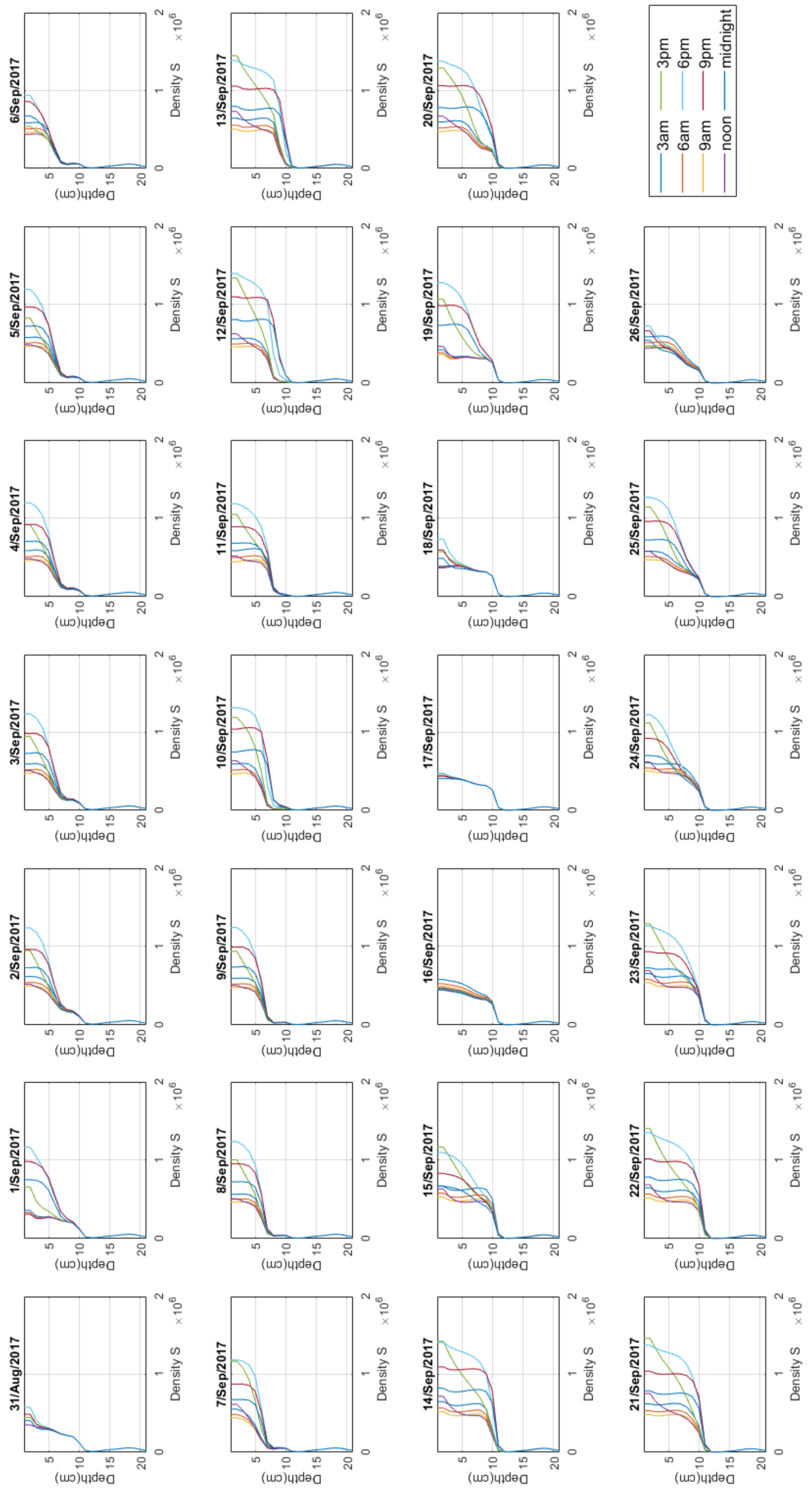

Figure S12: Dynamics of vertical distribution of susceptible bacteria S in soil (up to 20cm) predicted by the spatial model in September 2017 in the Nakhon Phanom province in Thailand. Bacterial density is measured in cell/ml.

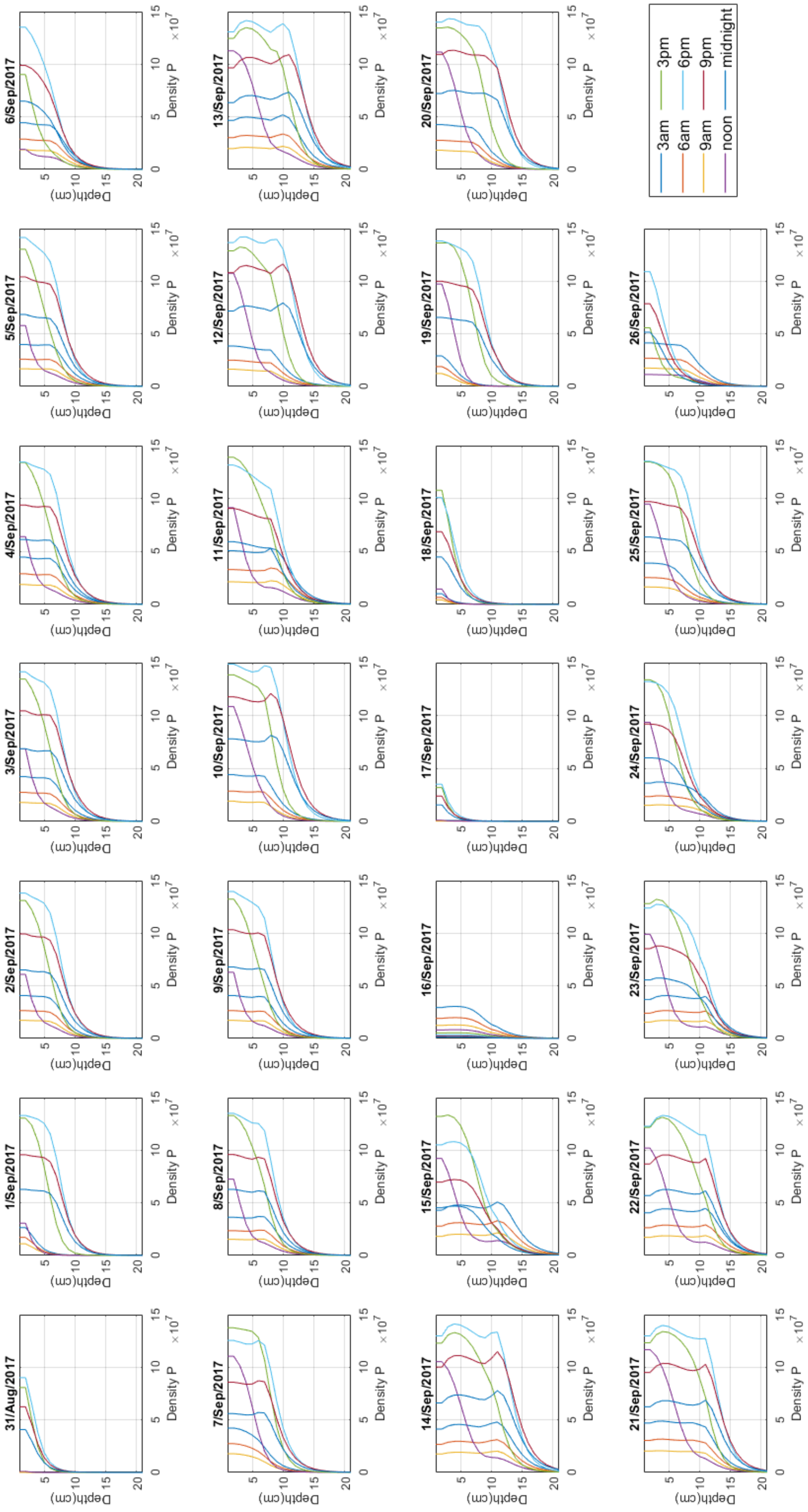

Figure S13: Dynamics of vertical distribution of free phage P in soil (up to 20cm) predicted by the spatial model in September 2017 in the Nakhon Phanom province in Thailand. Phage density is measured in pfu/ml.

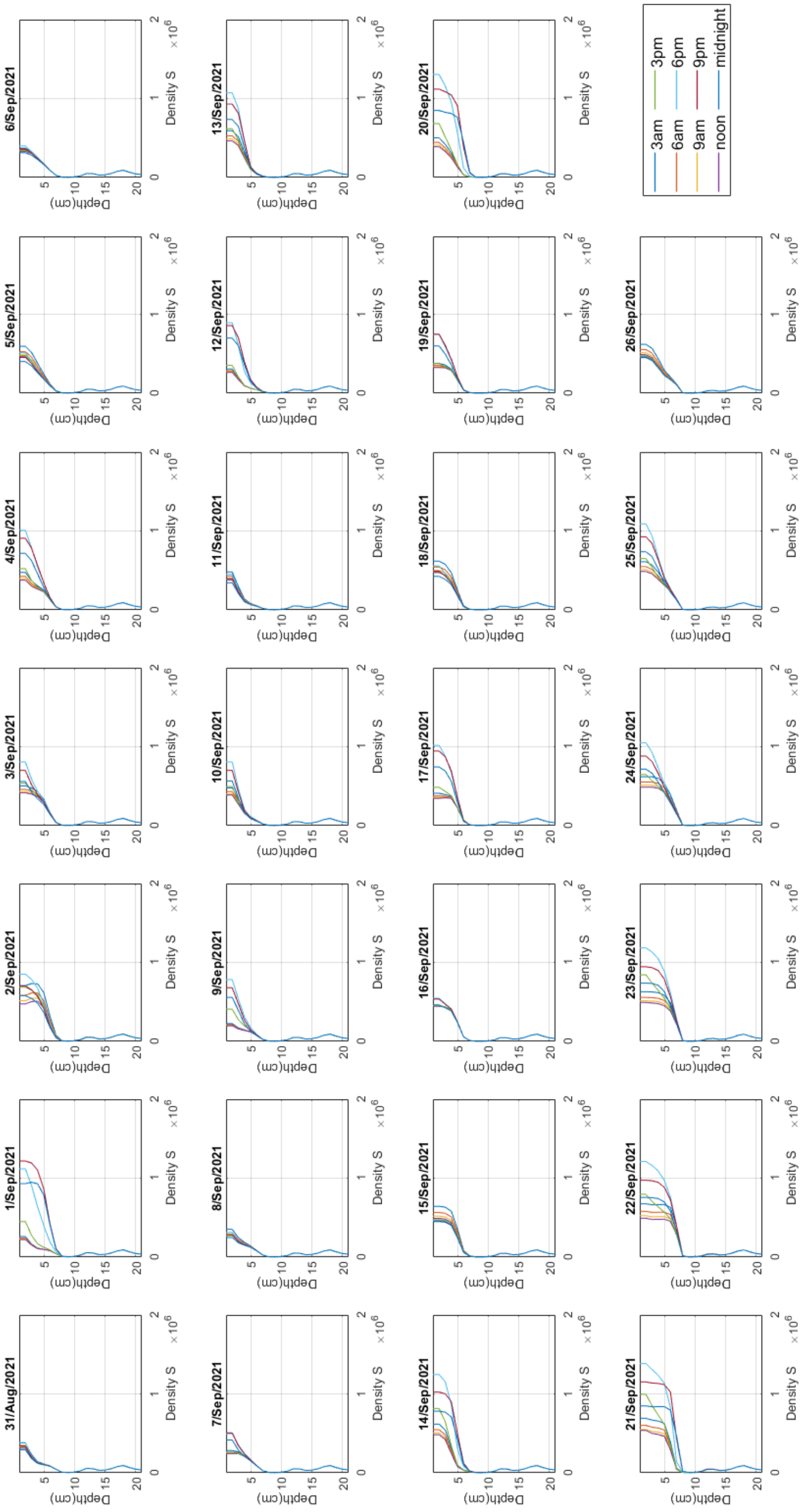

Figure S14: Dynamics of vertical distribution of susceptible bacteria S in soil (up to 20cm) predicted by the spatial model in September 2021 in the Nakhon Phanom province in Thailand. Bacterial density is measured in cell/ml.

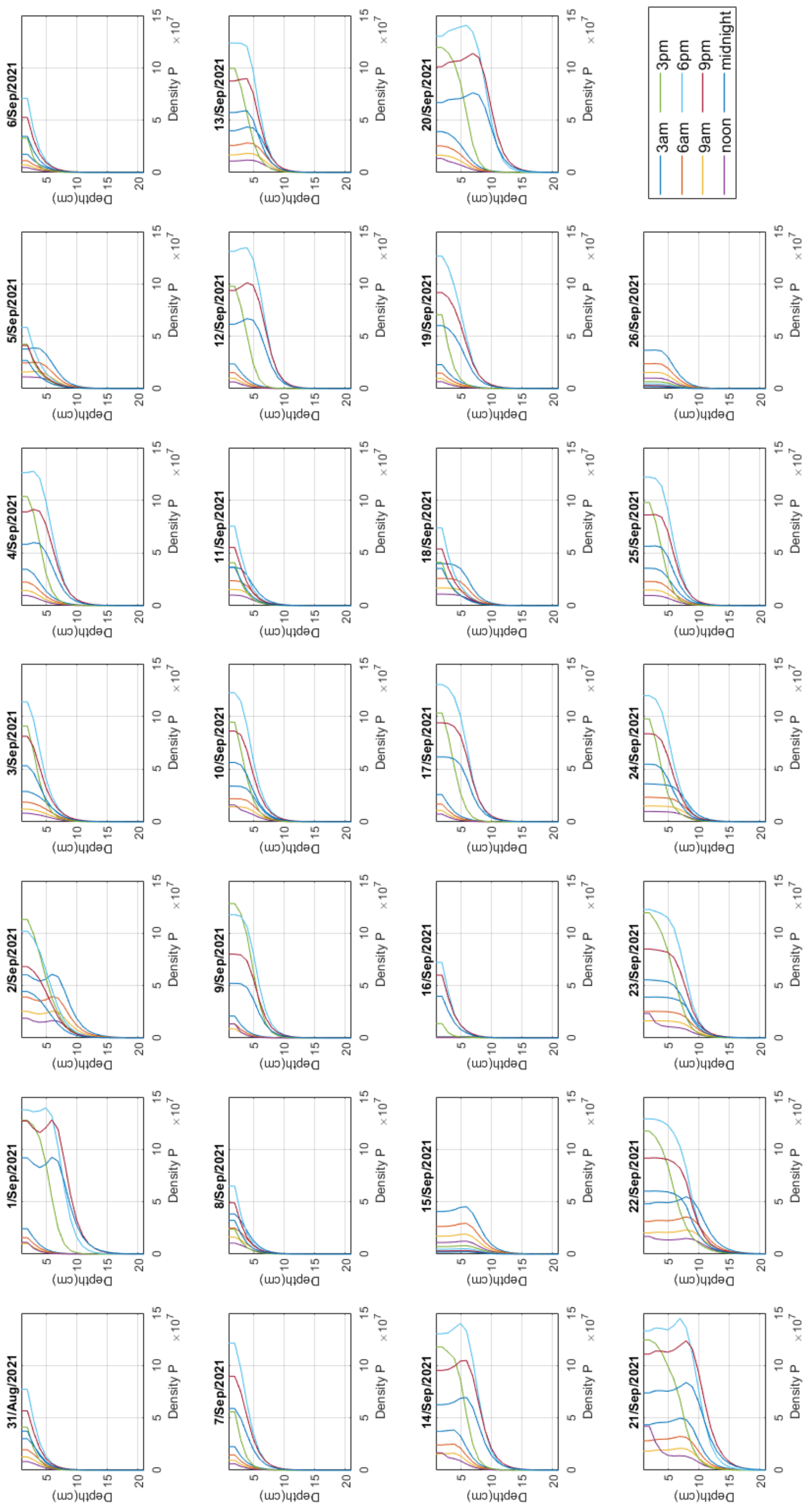

Figure S15: Dynamics of vertical distribution of free phage P in soil (up to 20cm) predicted by the spatial model in September 2021 in the Nakhon Phanom province in Thailand. Phage density is measured in pfu/ml.

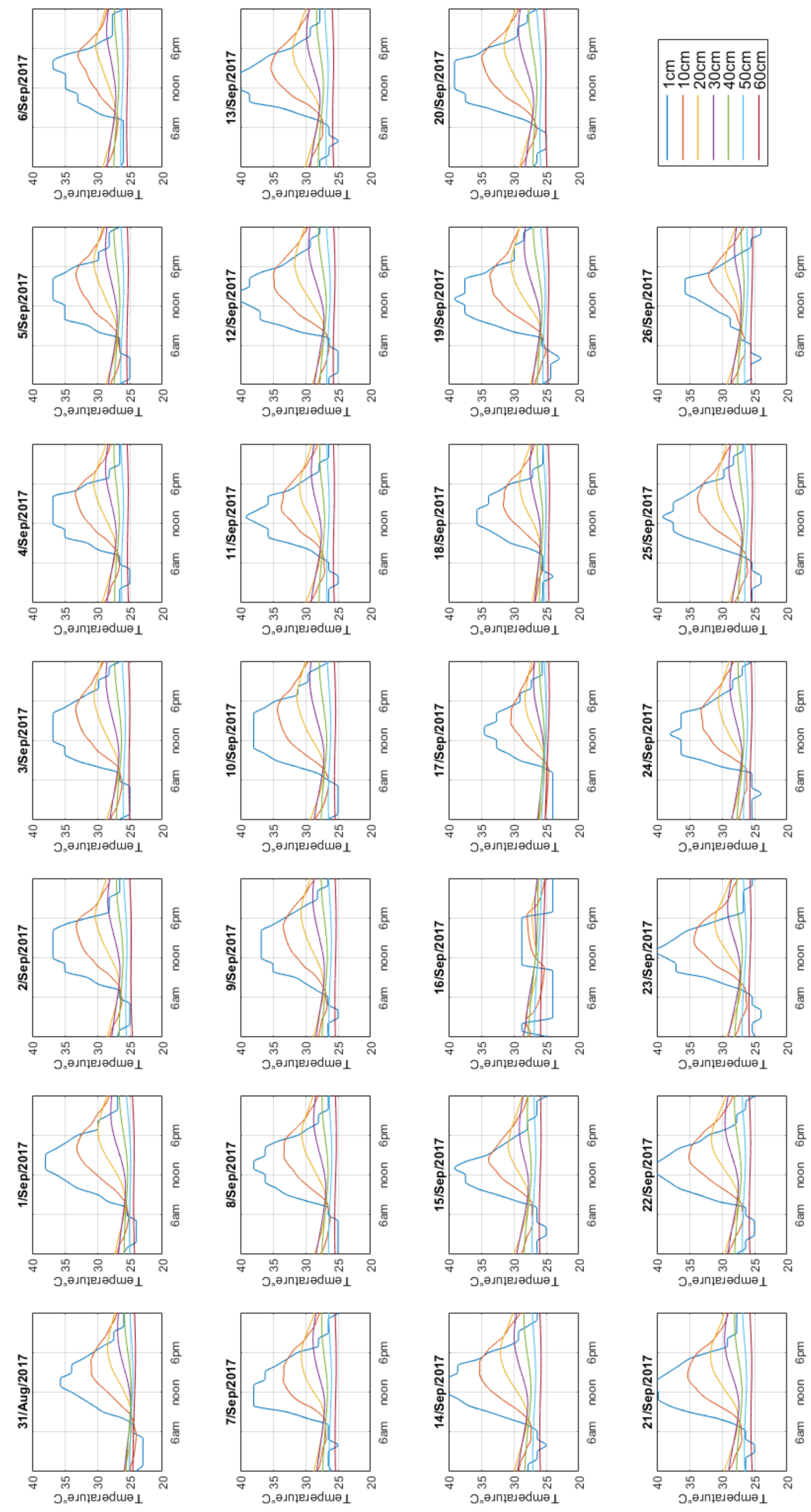

Figure S16: Daily temperature fluctuation predicted by the spatial model in September 2017 in the Nakhon Phanom province in Thailand. Each curve corresponds to a particular depth.

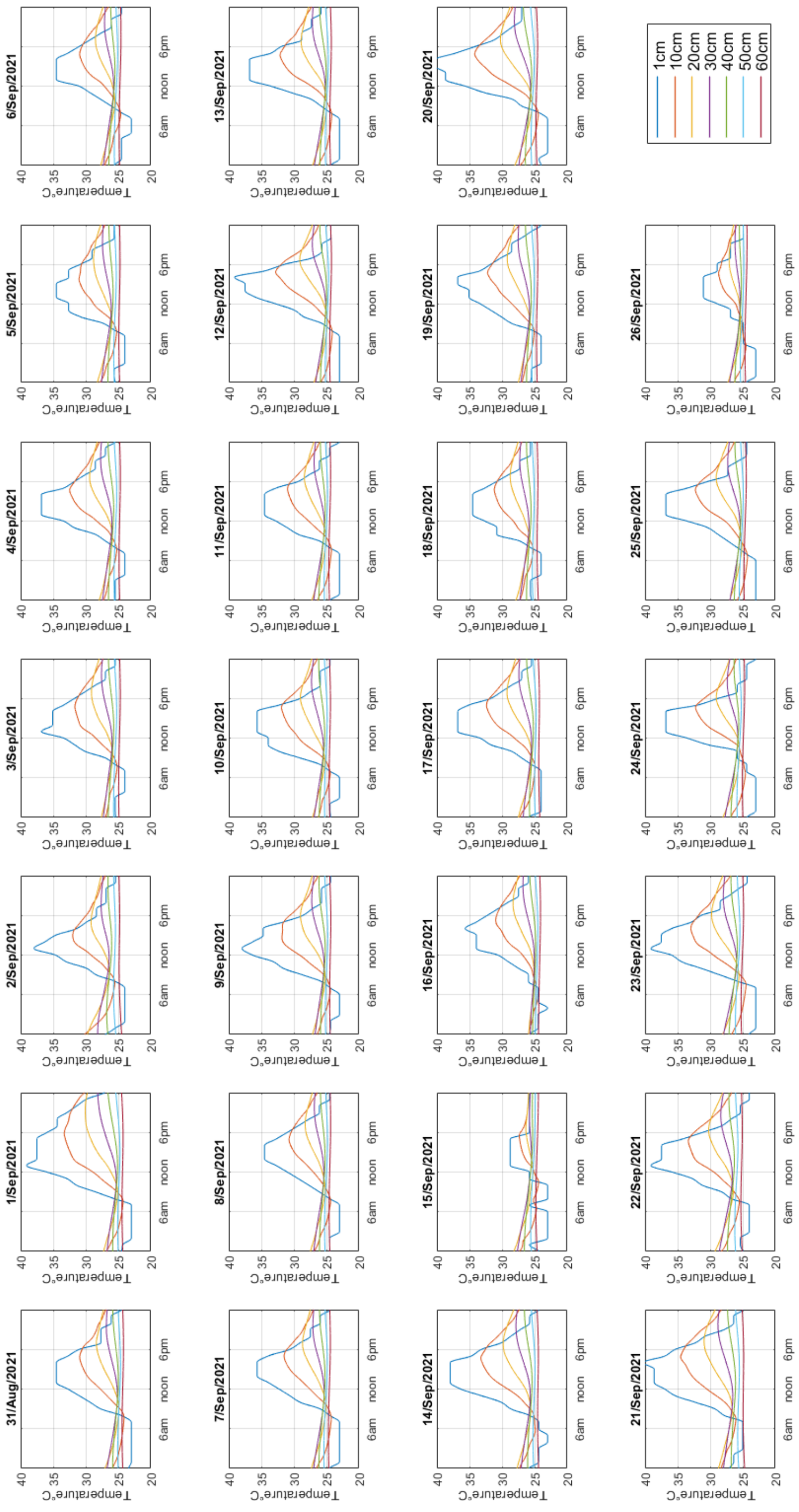

Figure S17: Daily temperature fluctuation predicted by the spatial model in September 2021 in the Nakhon Phanom province in Thailand. Each curve corresponds to a particular depth.

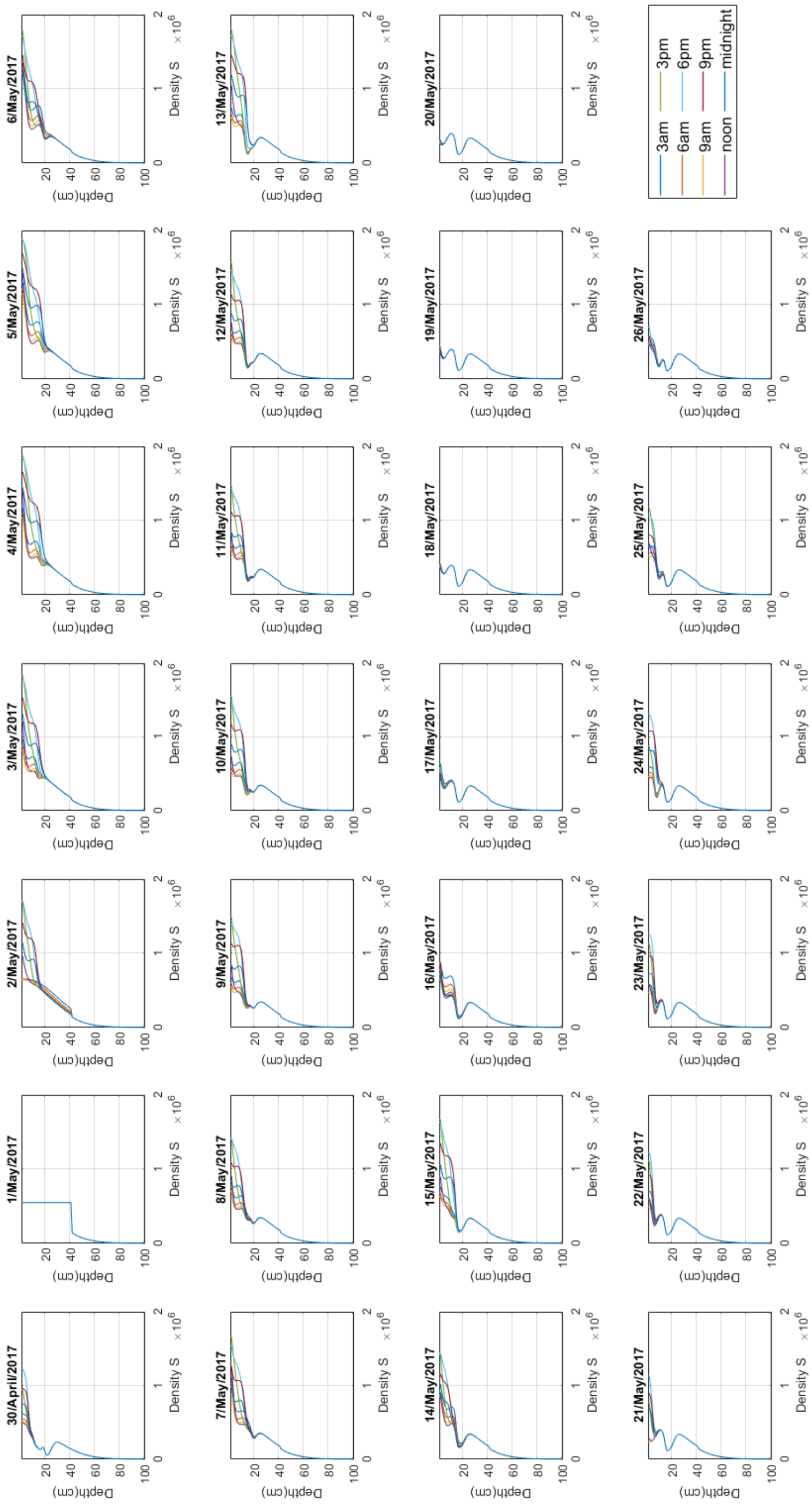

Figure S18: Dynamics of vertical distributions of susceptible bacteria S in soil after mixing the upper soil layer (up to 40cm). Soil mixing occurred on May 1st, 2017 (Nakhon Phanom province, Thailand). Bacterial density is measured in cell/ml.

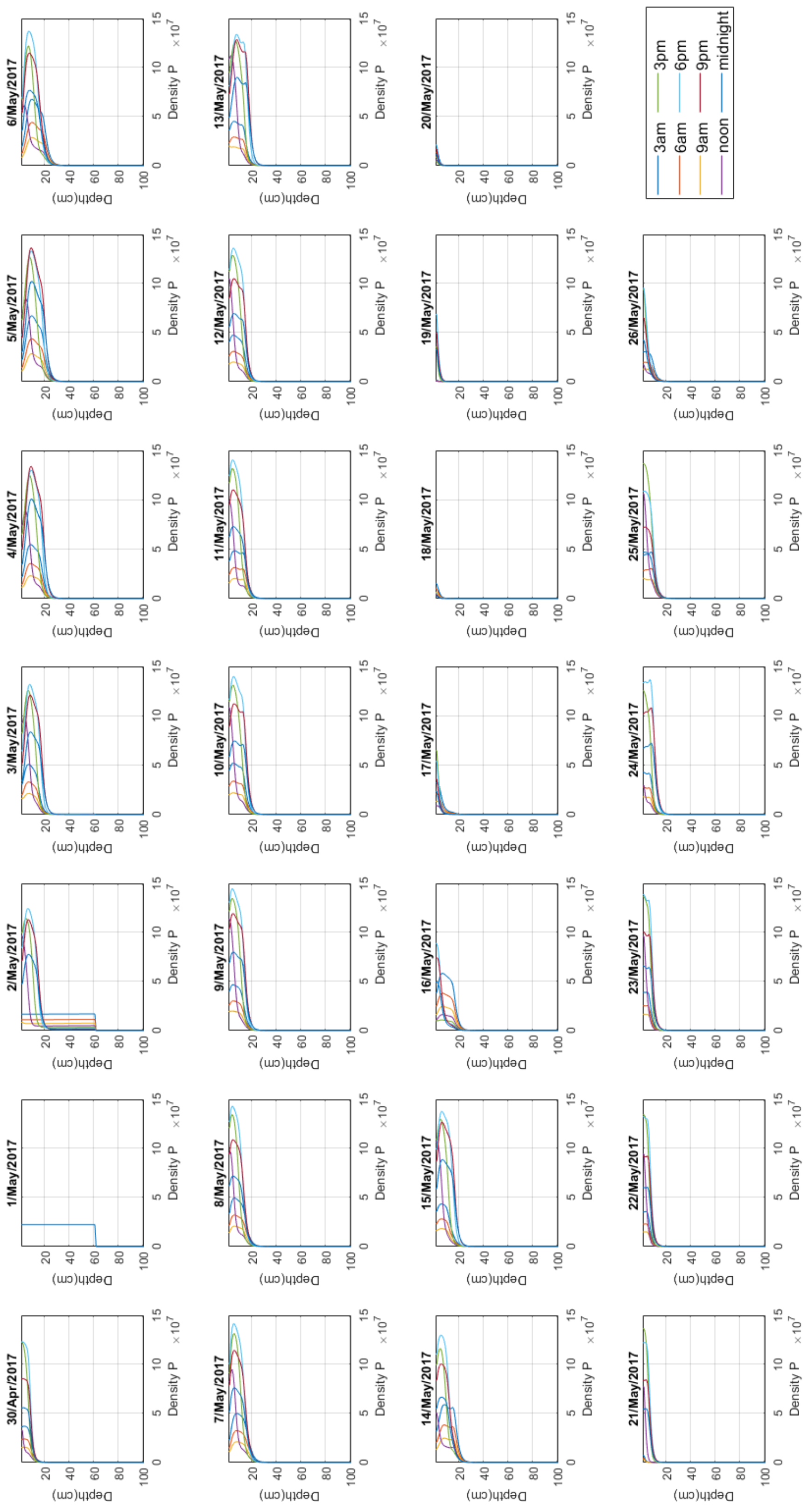

Figure S19: Dynamics of vertical distributions of the free P in soil after mixing the upper soil layer (up to 40cm). Soil mixing occurred on May 1st, 2017 (Nakhon Phanom province, Thailand). Phage density is measured in pfu/ml.

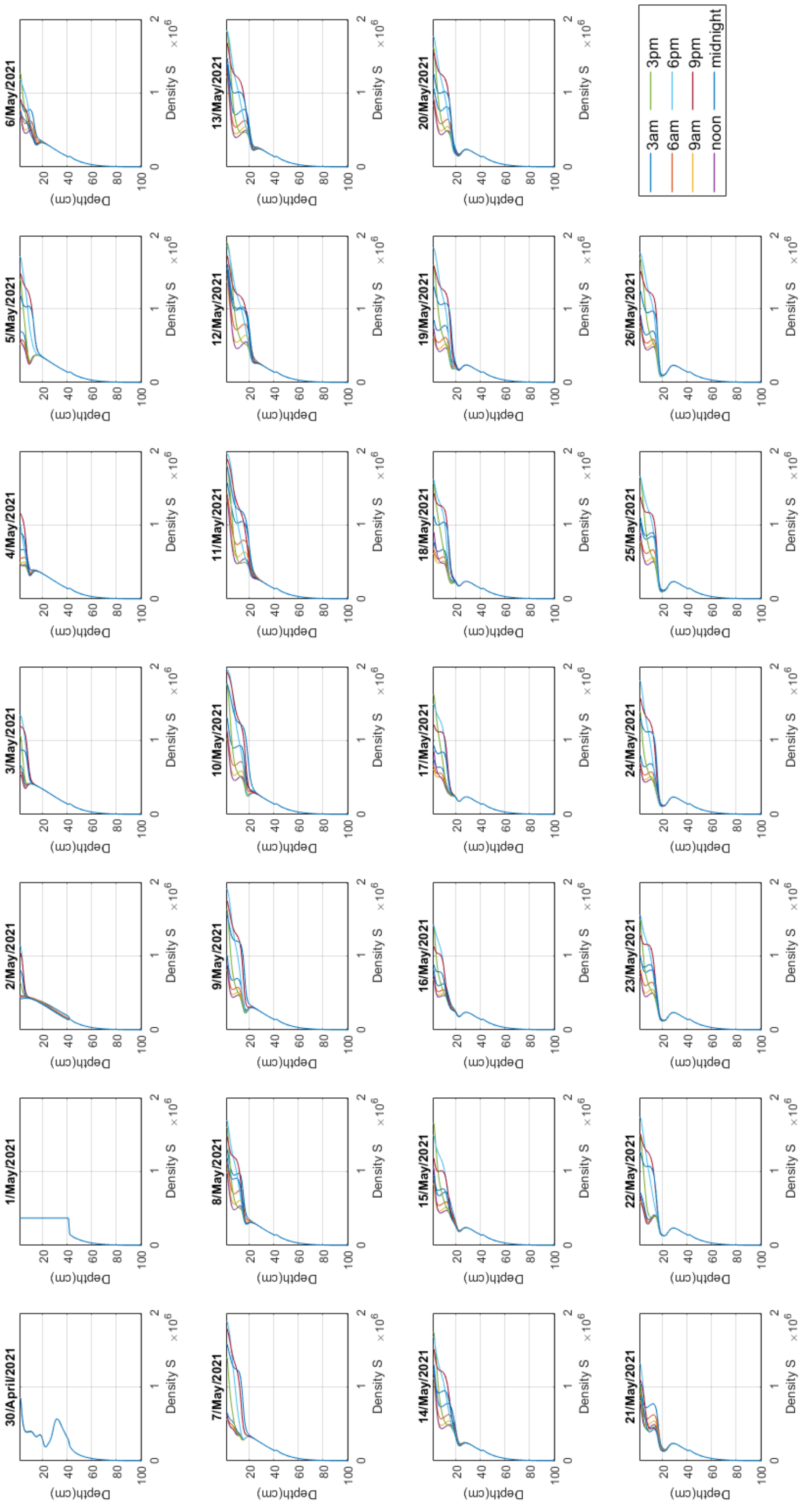

Figure S20: Dynamics of vertical distributions of susceptible bacteria S in soil after mixing the upper soil layer (up to 40cm). Soil mixing occurred on May 1st, 2021 (Nakhon Phanom province, Thailand). Bacterial density is measured in cell/ml.

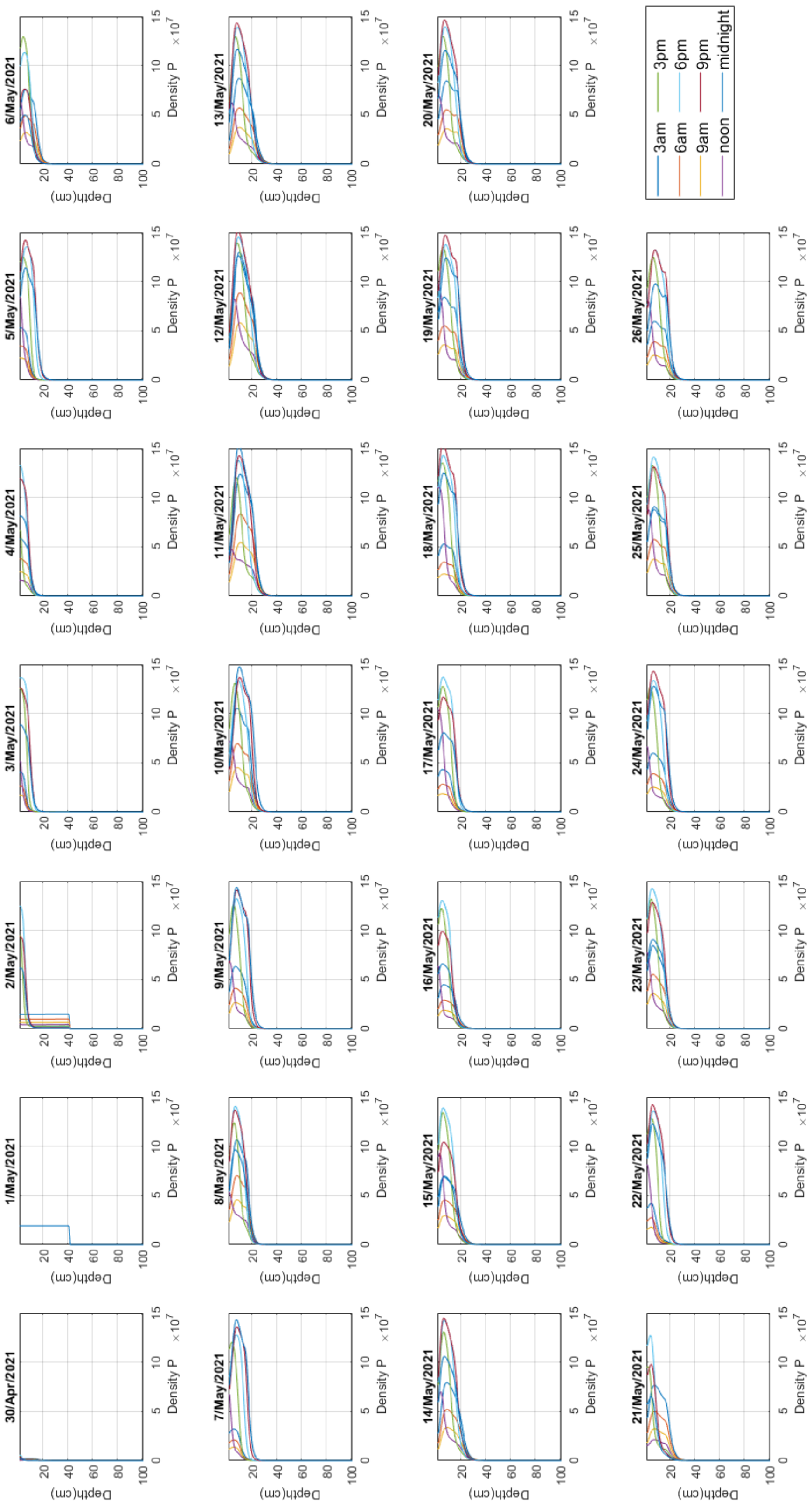

Figure S21: Dynamics of vertical distributions of the free P in soil after mixing the upper soil layer (up to 40cm). Soil mixing occurred on May 1st, 2021 (Nakhon Phanom province, Thailand). Phage density is measured in pfu/ml.

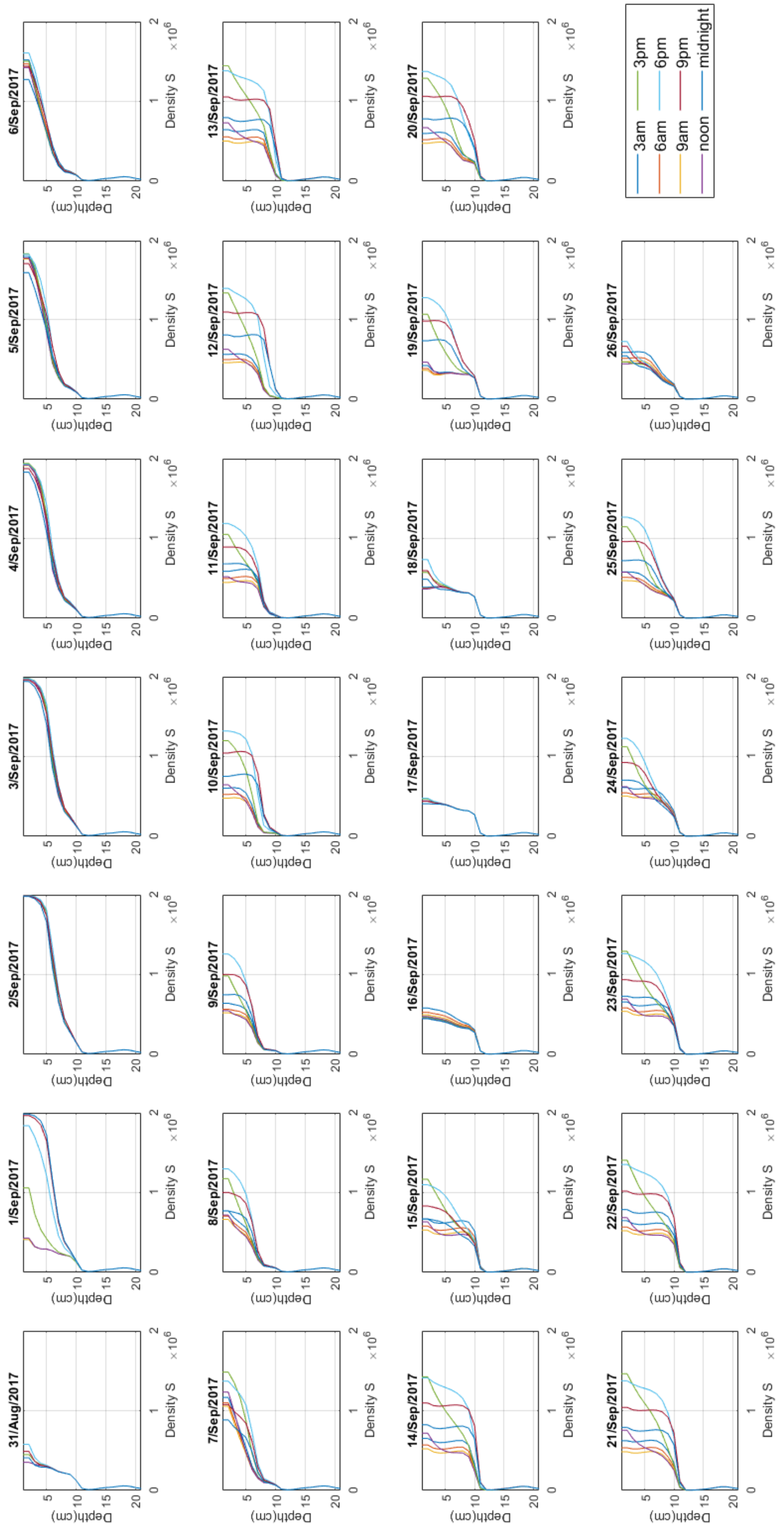

Figure S22: Dynamics of vertical distributions of susceptible bacteria S in soil (up to 20cm) after removing the free phage within the top 5cm depth. Phage removal occurred on September 1st, 2017 (Nakhon Phanom province, Thailand). Bacterial density is measured in cell/ml.

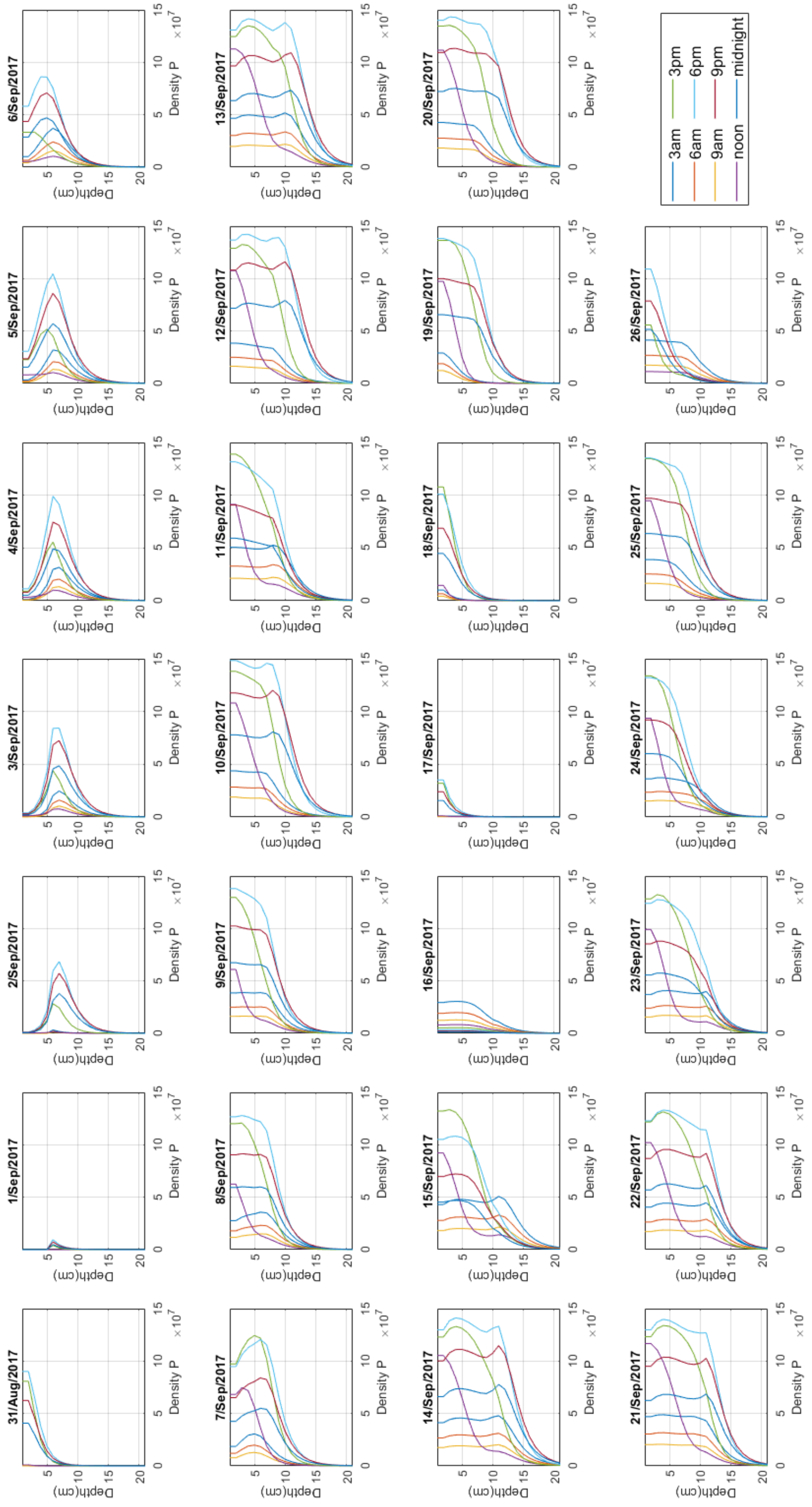

Figure S23: Dynamics of vertical distributions of free phage P in soil (up to 20cm) after removing the free phage within the top 5cm depth. Phage removal occurred on September 1st, 2017 (Nakhon Phanom province, Thailand). Phage density is measured in pfu/ml.

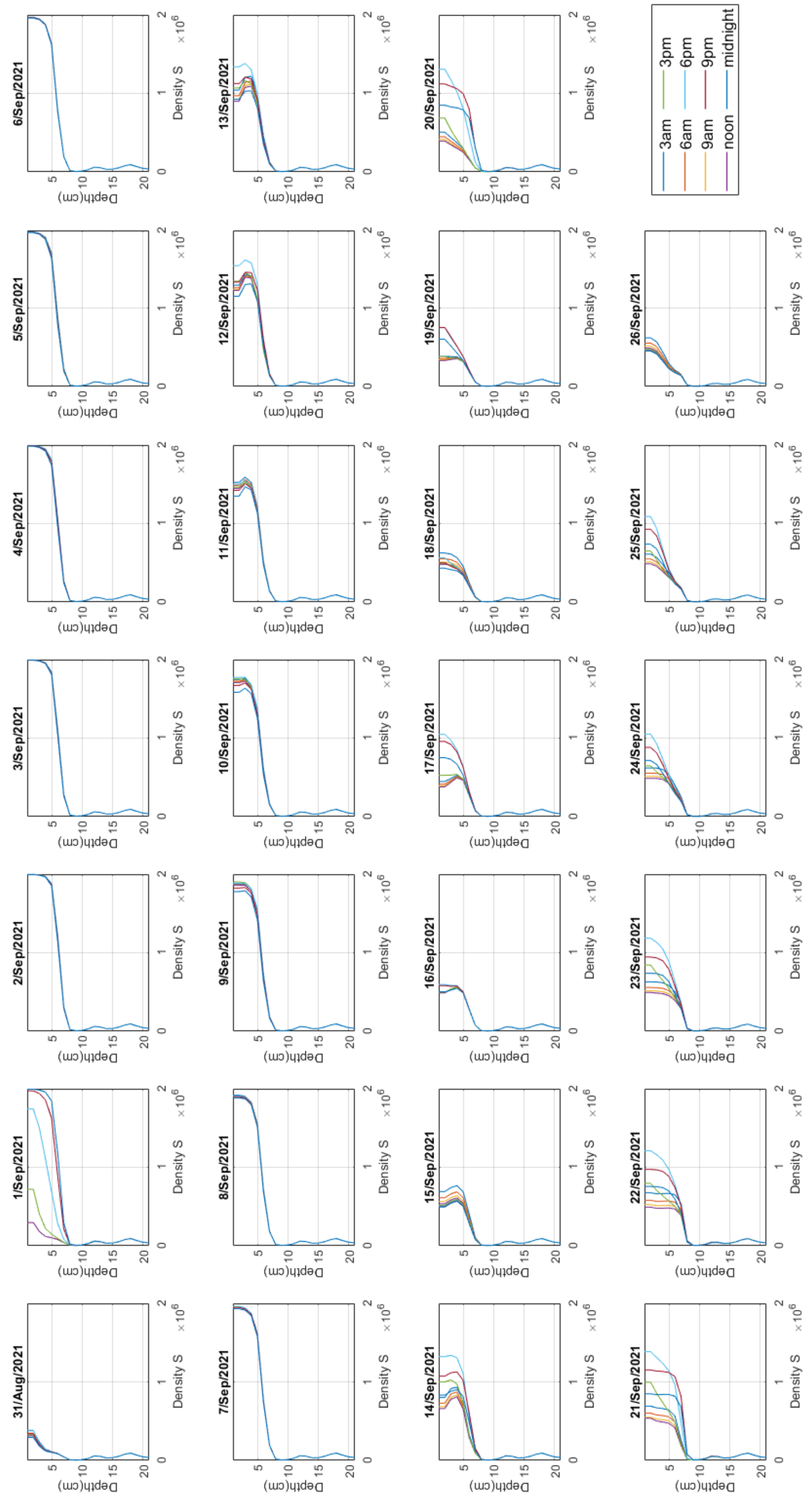

Figure S24: Dynamics of vertical distributions of susceptible bacteria S in soil (up to 20cm) after removing the free phage within the top 5cm depth. Phage removal occurred on September 1st, 2021 (Nakhon Phanom province, Thailand). Bacterial density is measured in cell/ml.

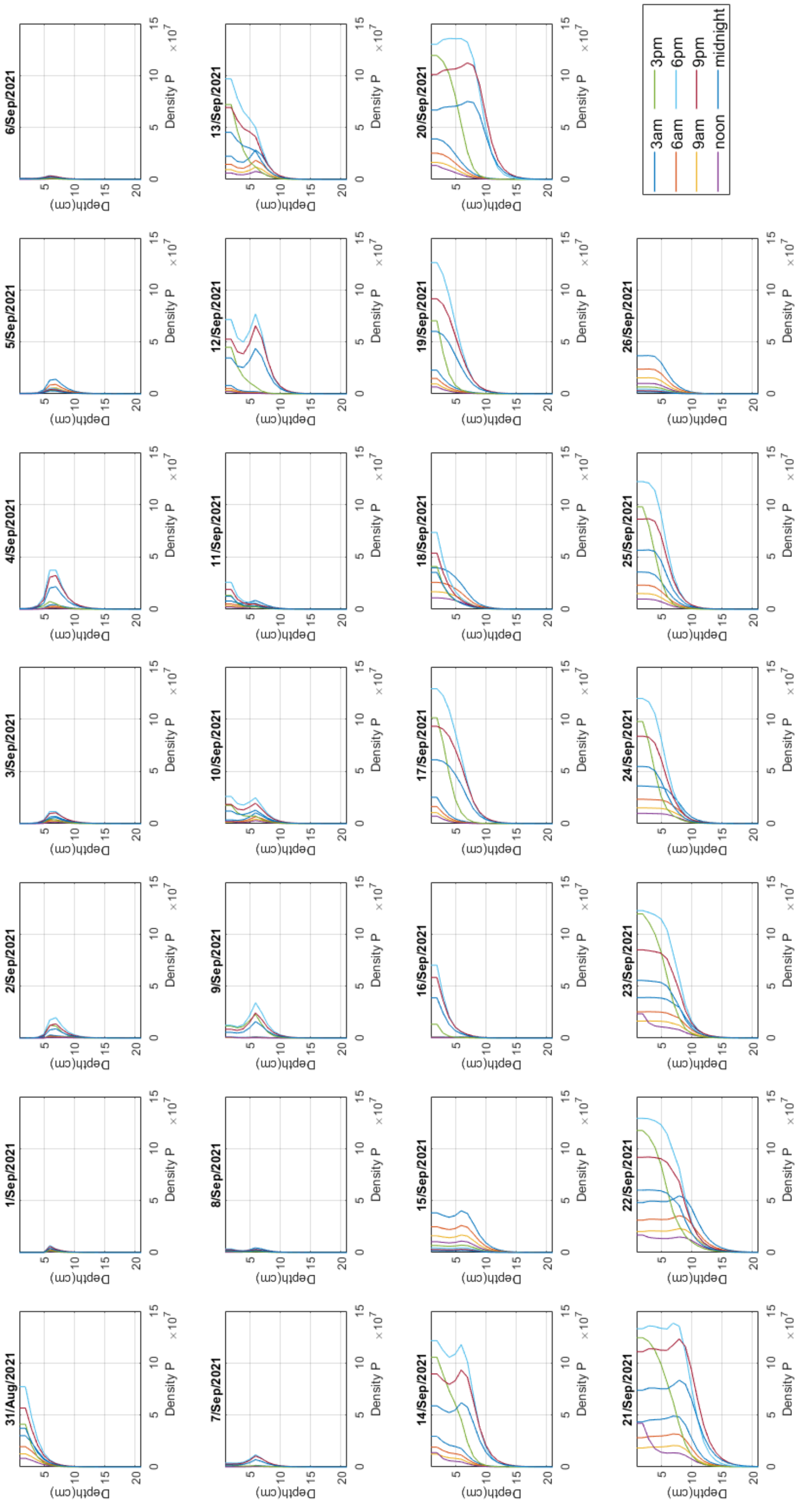

Figure S25: Dynamics of vertical distributions of free phage P in soil (up to 20cm) after removing the free phage within the top 5cm depth. Phage removal occurred on September 1st, 2021 (Nakhon Phanom province, Thailand). Phage density is measured in pfu/ml.

References

Brett, P., Deshazer, D., & Woods, D. (1997). Characterization of burkholderia pseudomallei and burkholderia pseudomallei-like strains. *Epidemiology & Infection*, *118*(2), 137–148.

Chen, Y., Chen, S., Kao, C., & Chen, Y. (2003). Effects of soil ph, temperature and water content on the growth ofburkholderia pseudomallei. *Folia microbiologica*, *48*(2), 253–256.

Egilmez, H. I., Morozov, A. Y., Clokie, M. R., Shan, J., Letarov, A., & Galyov, E. E. (2018). Temperature-dependent virus lifecycle choices may reveal and predict facets of the biology of opportunistic pathogenic bacteria. *Scientific Reports*, *8*(1), 9642.

Egilmez, H. I., Morozov, A. Y., & Galyov, E. E. (2021). Modelling the spatiotemporal complexity of interactions between pathogenic bacteria and a phage with a temperature-dependent life cycle switch. *Scientific reports*, *11*(1), 4382.

Gatedee, J., Kritsiriwuthinan, K., Galyov, E. E., Shan, J., Dubinina, E., Intarak, N., … Korbsrisate, S. (2011). Isolation and characterization of a novel podovirus which infects burkholderia pseudomallei. *Virology Journal*, *8*(1), 366.

Letarov, A., Letarova, M., Adler, N. L., Kulikov, E., Clokie, M., Morozov, A. Y., & Galyov, E. (2022). Effect of chemical factors on natural biocontrol of the melioidosis agent by amp1-like bacteriophages in agricultural ecosystems. *Microbiology*, *91*(2), 192–198.

Shan, J., Korbsrisate, S., Withatanung, P., Adler, N. L., Clokie, M. R., & Galyov, E. E. (2014). Temperature dependent bacteriophages of a tropical bacterial pathogen. *Frontiers in Microbiology*, *5*, 599.
